# Pseudo-Additive Tsallis Entropy and Non-Factorizing Joint Statistics in Product Sheffer Stroke Basic Algebras

**DOI:** 10.3390/e28080940

**Published:** 2026-08-21

**Authors:** Ibrahim Senturk, Metin Bilge, Tahsin Oner

**Affiliations:** 1 Department of Mathematics, Faculty of Science, Ege University, İzmir 35100, Türkiye; ibrahim.senturk@ege.edu.tr (I.S.); tahsin.oner@ege.edu.tr (T.O.); 2 Department of Physics, Faculty of Science, Ege University, İzmir 35100, Türkiye

**Keywords:** Sheffer stroke basic algebra, Tsallis entropy, Riečan state, non-factorizing joint statistics, non-distributive algebraic logic, 03G12, 03G25, 94A17, 68W30, 08A99

## Abstract

This paper addresses the problem of formulating generalized, non-extensive information-theoretic measures on finite non-distributive algebraic structures equipped with Riečan states, with particular emphasis on product Sheffer stroke basic algebras. Our approach formalizes finite summations, admissible partitions, refinement relations, and Sheffer stroke joint refinement candidates by using the primitive Sheffer stroke operation, with partition and marginalization properties imposed under the stated product and admissibility assumptions. By leveraging the state-theoretic properties of Riečan states, we construct baseline Shannon and logical entropies alongside algorithmic procedures for their computational evaluation. As the main result, we introduce and analytically characterize a parametric Tsallis entropy functional over these basic algebras. We prove its fundamental properties, including bounding inequalities, state concavity, monotonicity under refinement, subadditivity (for α>1), conditional chain-type identities under the relevant joint refinement marginalization assumptions, and exact analytical convergence to the classical Shannon limit as the entropic index α→1. Furthermore, under a state-dependent statistical independence condition, we show that the joint Tsallis entropy satisfies a pseudo-additive relation. By defining the Tsallis mutual information and the associated pseudo-additive residual, we isolate the deviation of a joint Sheffer stroke refinement from the factorized model determined by its marginal Riečan-state distributions. This residual is intended as a state-dependent algebraic indicator of deviations from the factorized Tsallis pseudo-additive model; it is not claimed to be an operational contextuality witness, a contextuality inequality, an entanglement measure, or a physical implementation criterion.

## 1. Introduction

Constructing mathematical models with maximal theoretical efficiency and a minimal set of primitive operations is a central pursuit in universal algebra. This structural minimalism eliminates redundancies, streamlining both theoretical analysis and computational implementations by relying strictly on equivalent, minimal statements. A paradigmatic example of this reduction is Tarski’s [1] axiomatization of abelian groups, which efficiently captured the structure using a minimal set of axioms based exclusively on a single divisor operator. Similarly, Sheffer [2] fundamentally altered structural logic by demonstrating that the entirety of Boolean functions can be generated solely through the Sheffer stroke (NAND) operation, establishing a foundational framework for subsequent generalized algebraic formulations [3].

In the realm of algebraic logic, the probabilistic interpretation of non-classical structures through measures and state operators has been extensively investigated. For MV-algebras, Mundici [4] introduced the concept of states as an algebraic analogue to classical probability measures, capturing the averaging of truth values within Łukasiewicz logic. This probabilistic framework was systematically expanded to BCK-algebras by Dvurečenskij [5,6] and further advanced in collaboration with Pulmannová and Dvurečenskij [7]. Ciungu and Dvurečenskij [8] extended state theory to pseudo-BCK-algebras by embedding quotient pseudo-BCK-algebras into the negative cone of abelian Archimedean groups. Concurrently, the theory of pseudo-valuations was developed across various non-classical logics, including residuated lattices [9], BCK-algebras [10,11], and BE-algebras [12], with commutative pseudo-valuations specifically introduced by Doh and Kang [13] for BCK-algebras.

The quantification of uncertainty within such spaces fundamentally relies on information theory, originally pioneered by Shannon [14] and systematically detailed by Gray [15]. For a classical experiment with *m* mutually exclusive outcomes having probabilities $q_{1},q_{2},\ldots ,q_{m}$, the Shannon entropy is defined as

(1) $$E=\sum_{j=1}^{m}g\left(q_{j}\right),$$

where g:[0,1]→[0,∞) is the Shannon entropy function, expressed as

(2) g(y)=−ylogy,fory∈[0,1](with0log0=0byconvention).

The scope of Shannon entropy was subsequently extended to dynamical systems by Kolmogorov [16] and Sinai [17] as an invariant metric to distinguish between isomorphic measure-preserving transformations, successfully demonstrating the existence of non-isomorphic Bernoulli shifts. The success of the Kolmogorov–Sinai entropy precipitated the investigation of alternative entropic measures. Markechová et al. [18] introduced the logical entropy L(S) for dynamical systems. By replacing the Shannon function with the logical entropy function l:[0,1]→[0,∞), defined as

(3) $$l\left(z\right)=z-z^{2},\;\mathrm{for}\;z\in \left[0,1\right],$$

results analogous to the classical Kolmogorov–Sinai theory were obtained, providing an alternative invariant tool for systemic distinction.

To generalize classical statistical mechanics, Tsallis [19] introduced a parametric entropy functional, structurally equivalent to the β-entropy developed earlier by Havrda and Charvát [20]. For a probability distribution $Q=\{q_{1},q_{2},\ldots ,q_{m}\}$ and an entropic index β∈(0,1)∪(1,∞), the Tsallis entropy is defined as

(4) $$T_{\beta }\left(Q\right)=\frac{1}{\beta -1}\left(1-\sum_{j=1}^{m}q_{j}^{\beta }\right).$$

By defining the core generalized function $h_{\beta }\left(y\right)=\frac{1}{\beta -1}\left(y-y^{\beta }\right)$, this can be rewritten in summation form as $T_{\beta }\left(Q\right)=\sum_{j=1}^{m}h_{\beta }\left(q_{j}\right)$. Notably, evaluating this formulation at β=2 exactly recovers the logical entropy of the probability distribution, as established by Ellerman [21]:

(5) $$T_{2}\left(Q\right)=\sum_{j=1}^{m}\left(q_{j}-q_{j}^{2}\right)=1-\sum_{j=1}^{m}q_{j}^{2}.$$

Tsallis statistics are widely studied in non-extensive statistical mechanics and in models involving long-range correlations, memory effects, or fractal-type structures. They have also motivated applications and theoretical developments across several areas, including physics, geophysics, chemistry, economics, and communication systems [22,23,24,25,26,27,28,29].

While the aforementioned studies operate within the classical framework of Kolmogorov probability theory, Zadeh [30] introduced fuzzy set theory to model uncertainty through subjective evaluations rather than objective measurements. The subsequent formalization of the probability of fuzzy events [31] led to extensive research on the entropy of fuzzy dynamical systems and related algebraic structures [32,33,34,35]. Tsallis entropy has also been successfully extended to product MV-algebras by Markechová and Riečan [36] and, more recently, directly in MV-algebras by Barbieri and Lenzi [37], complementing earlier extensions of Shannon entropy to product MV-algebras [38].

The present work is not intended to replace these MV-algebraic constructions, but to develop a parallel entropy theory in a different algebraic language. In the MV-algebraic setting, the treatment of decompositions and common refinements is naturally governed by the Riesz decomposition/refinement machinery; see, for instance, Mundici [39]. In product MV-algebras, the joint experiment is instead defined by using an additional binary product operation in the algebraic signature. By contrast, in the present product Sheffer stroke basic algebra framework, joint components are constructed natively from the single primitive operation by

(6) $$\zeta_{ij}=\left(\xi_{i}|\upsilon_{j}\right)|\left(\xi_{i}|\upsilon_{j}\right).$$

Thus, the point is not that MV-algebraic joint refinements are unavailable, but that our construction avoids relying on the MV-algebraic Riesz refinement mechanism or on an additional product operation. The corresponding restriction is that one no longer assumes an associative product operation or a Riesz refinement-based joint construction independent of recursive choices. Consequently, finite Sheffer sums are defined recursively and evaluated with a fixed convention.

This comparison also separates the analogue results from the Sheffer stroke-specific contributions of the paper. The definitions of marginal Tsallis entropy, the limiting transition to Shannon entropy as α→1, the entropy bounds, monotonicity under refinement, state concavity, conditional entropy, subadditivity for α>1, and pseudo-additivity under state factorization are structural analogues of the known MV-algebraic results [36,37]. However, the present framework is not formulated in terms of Riesz refinements, and the corresponding properties are proven without invoking the MV-algebraic Riesz decomposition machinery or an additional product operation. Instead, the proofs are specific to the present Sheffer stroke framework because they rely on orthogonal Sheffer sums, stroke-native joint refinement components satisfying the stated admissibility conditions, orthogonal distributivity, and Riečan-state additivity over orthogonal components.

The genuinely new aspects are therefore the single-operation formulation of partitions and admissible joint refinement components, the algorithmic implementation of entropy calculations through the stroke operation, and the pseudo-additive residual, which records deviations from state factorization at the level of the Sheffer stroke joint state values determined by the marginal Riečan-state distributions.

To clarify the relation with the existing MV-algebraic literature explicitly, Table 1 summarizes the technical distinctions between these frameworks.

The general notions concerning Sheffer stroke basic algebras and state-based algebraic structures recalled in this paper are included only to provide the necessary background for the reader. The original contribution of the present work begins with the systematic formulation of entropy functionals on product Sheffer stroke basic algebras. More precisely, we introduce Shannon, logical, and Tsallis entropies in this setting and establish their main structural properties, including subadditivity, concavity, conditional forms, and related information-theoretic constructions.

Extending entropy calculus beyond classical probability theory to generalized non-distributive algebraic structures provides a perspective on uncertainty measurement in abstract algebraic settings. Possible connections with quantum-logical, computational, and uncertainty-modeling frameworks are treated here only at a structural or prospective level.

The remainder of this manuscript is organized as follows. Section 2 establishes the fundamental algebraic concepts, partial order properties, and the formalization of Riečan state operators utilized throughout the study. Section 3 introduces the foundational information-theoretic constructs within a product Sheffer stroke basic algebra B=(B;∣). Here, we define recursive finite summations and partitions, subsequently formulating the classical Shannon and logical entropies alongside explicit algorithmic evaluations and proofs of subadditivity. Finally, Section 4 introduces a parametric generalization via the Tsallis entropy functional. We derive its analytical properties, including bounding inequalities, state concavity, and conditional chain-type identities under the relevant joint refinement assumptions. Most notably, we demonstrate its smooth convergence to Shannon entropy as the entropic parameter trends to one, establish its pseudo-additive relation under state factorization, and formulate the Tsallis mutual information together with a pseudo-additive residual as a state-dependent algebraic indicator of non-factorizing joint statistics within the present product Sheffer stroke framework.

## 2. Preliminaries

The fundamental algebraic and information-theoretic concepts utilized throughout this paper are established in this section.

**Definition** **1** ([40])**.** *An algebra B=(B;⊕,¬,0) is called a basic algebra if it satisfies the following axioms for all ξ,υ,θ∈B:*

(*BA*1) *ξ⊕0=ξ,*
(*BA*2) *¬¬ξ=ξ,*
(*BA*3) *¬(¬ξ⊕υ)⊕υ=¬(¬υ⊕ξ)⊕ξ,*
(*BA*4) *$\neg \left(\neg (\neg (\neg \xi ⊕\upsilon )⊕\upsilon )⊕\theta \right)⊕\left(\xi ⊕\theta \right)=\neg 0$.*

**Definition** **2** ([41])**.** *] Let B=(B;∣) be a groupoid. The operation ∣:B×B→B is called a Sheffer stroke operation if the following conditions are satisfied for all ξ,υ,θ∈B:*

(*S*1) *ξ∣υ=υ∣ξ,*
(*S*2) *(ξ∣ξ)∣(ξ∣υ)=ξ,*
(*S*3) *$\xi |\left((\upsilon |\theta )|(\upsilon |\theta )\right)=\left((\xi |\upsilon )|(\xi |\upsilon )\right)|\theta$,*
(*S*4) *$\left(\xi |\left((\xi |\xi )|(\upsilon |\upsilon )\right)\right)|\left(\xi |\left((\xi |\xi )|(\upsilon |\upsilon )\right)\right)=\xi$.*

*If, additionally, the identity*

(*S*5) $\upsilon |\left(\xi |(\xi |\xi )\right)=\upsilon |\upsilon$

*is satisfied, the operation is called an ortho-Sheffer stroke operation.*

**Lemma** **1** ([41])**.** *Assume that* ∣ *is a Sheffer stroke operation on B and ≤ is the induced partial order of B=(B;∣). Then for all ξ,υ,θ,ω∈B:*

(*i*) *ξ≤υ⇔υ∣υ≤ξ∣ξ,*
(*ii*) *$\xi |\left(\upsilon |(\xi |\xi )\right)=\xi |\xi$,*
(*iii*) *ξ≤υ⇒υ∣θ≤ξ∣θ,*
(*iv*) *ω≤ξ and ω≤υ⇒ξ∣υ≤ω∣ω.*

**Definition** **3** ([42])**.** *An algebra (B;∣) of type 〈2〉 is called a Sheffer stroke basic algebra if it satisfies the following identities for all ξ,υ,θ∈B:*

(*SH*1) *$\left(\xi |(\xi |\xi )\right)|\left(\xi |\xi \right)=\xi$,*
(*SH*2) *$\left(\xi |(\upsilon |\upsilon )\right)|\left(\upsilon |\upsilon \right)=\left(\upsilon |(\xi |\xi )\right)|\left(\xi |\xi \right)$,*
(*SH*3) *$\left(\left((\xi |(\upsilon |\upsilon ))|(\upsilon |\upsilon )\right)|\left(\theta |\theta \right)\right)|\left(\left(\xi |(\theta |\theta )\right)|\left(\xi |(\theta |\theta )\right)\right)=\xi |\left(\xi |\xi \right)$.*

Sheffer stroke basic algebras exhibit foundational algebraic properties, including the existence of a constant upper bound 1, analogous to implication basic algebras [40].

**Lemma** **2** ([42])**.** *Assume that (B;∣) is a Sheffer stroke basic algebra. Then the element 1 is a constant in B, and the structure (B;∣) satisfies the following identities for all ξ,υ∈B:*

(*i*) *ξ∣(ξ∣ξ)=1,*
(*ii*) *ξ∣(1∣1)=1,*
(*iii*) *1∣(ξ∣ξ)=ξ,*
(*iv*) *$\left((\xi |(\upsilon |\upsilon ))|(\upsilon |\upsilon )\right)|\left(\upsilon |\upsilon \right)=\xi |\left(\upsilon |\upsilon \right)$,*
(*v*) *$\left(\upsilon |\left(\xi |(\upsilon |\upsilon )\right)\right)|\left(\xi |(\upsilon |\upsilon )\right)=1$.*

To elucidate the algebraic structure of Sheffer stroke basic algebras, an induced partial order is defined on *B*.

**Lemma** **3** ([42])**.**  *Assume that (B;∣) is a Sheffer stroke basic algebra. A binary relation ≤ is defined on B as follows:*

ξ≤υ⇔ξ∣(υ∣υ)=1.

*Then the binary relation ≤ is a partial order on B such that ξ≤1 for all ξ∈B. Moreover, for all ξ,υ,θ∈B:*

$$\theta \leq \left(\xi |(\theta |\theta )\right)\;\mathrm{and}\;\xi \leq \upsilon \Rightarrow \upsilon |\left(\theta |\theta \right)\leq \xi |\left(\theta |\theta \right).$$

The partial order ≤ formalized in Lemma 3 establishes a structural bridge between Sheffer stroke basic algebras and lattice structures, as detailed in [42].

**Corollary** **1** ( [42])**.** *Let (B;∣) be a Sheffer stroke basic algebra with the least element 0 and the greatest element 1. Then (B;∨,∧,¬,0,1) is a lattice with an antitone involution.*

**Definition** **4** ([43])**.** *A mapping $\tau^{R}:B\to \left[0,1\right]$ is called a Riečan state on B=(B;∣) if it verifies the following conditions for all ξ,υ∈B:*

$\tau_{R}^{SH}$1 *      $\tau^{R}\left(1\right)=1$,*
$\tau_{R}^{SH}$2 *      $\tau^{R}\left((\xi |\xi )|(\upsilon |\upsilon )\right)=\tau^{R}\left(\xi \right)+\tau^{R}\left(\upsilon \right)$, provided that ξ∣υ=1.*

**Lemma** **4** ([43])**.** *Let $\tau^{R}:B\to \left[0,1\right]$ be a Riečan state on B=(B;∣). Then, the following properties are satisfied for all ξ,υ∈B:*

(*i*) *$\tau^{R}\left(0\right)=0$,*
(*ii*) *$\tau^{R}\left(\xi |\xi \right)=1-\tau^{R}\left(\xi \right)$,*
(*iii*) *If ξ∣(υ∣υ)=1, then $\tau^{R}\left(\xi \right)\leq \tau^{R}\left(\upsilon \right)$,*
(*iv*) *$\tau^{R}\left((\xi |\upsilon )|\upsilon \right)=\tau^{R}\left((\xi |\upsilon )|(\xi |\upsilon )\right)+\tau^{R}\left(\upsilon |\upsilon \right)$.*

## 3. Partitions and Foundational Entropy Measures in Product Sheffer Stroke Basic Algebras

In this section, we establish the foundational algebraic and information-theoretic constructs within a product Sheffer stroke basic algebra B=(B;∣), setting the groundwork for the generalized Tsallis entropy framework presented in the subsequent section. We begin by defining recursive finite summations, valid partitions, and joint partition refinements exclusively through the fundamental Sheffer stroke operation. Leveraging the metric properties of Riečan states, we introduce the classical Shannon entropy and the logical entropy of these partitions, formulating their conditional extensions and providing explicit algorithmic evaluations. By proving the core subadditivity properties of these baseline measures, we construct the necessary structural comparative baseline. This non-distributive, logic-based foundation establishes the requisite framework to subsequently introduce the non-extensive Tsallis entropy, enabling a formal analysis of how generalized parameterized entropies degenerate into these classical limits.

**Definition** **5.** *Let B=(B;∣) be a Sheffer stroke basic algebra. We first define the derived binary operation*

(7) x⊕y:=(x∣x)∣(y∣y),x,y∈B.

*For any n∈N and any finite sequence $\xi_{1},\xi_{2},\ldots ,\xi_{n}\in B$, we define the iterated finite operation ${⨁}_{i=1}^{n}\xi_{i}$ recursively by*

(8) $$\overset{1}{\underset{i=1}{⨁}}\xi_{i}:=\xi_{1},$$

*and, for n>1,*

(9) $$\overset{n}{\underset{i=1}{⨁}}\xi_{i}:=\left(\overset{n-1}{\underset{i=1}{⨁}}\xi_{i}\right)⊕\xi_{n}.$$

*Equivalently,*

(10) $$\overset{n}{\underset{i=1}{⨁}}\xi_{i}=\left[\left(\overset{n-1}{\underset{i=1}{⨁}}\xi_{i}\right)|\left(\overset{n-1}{\underset{i=1}{⨁}}\xi_{i}\right)\right]|\left(\xi_{n}|\xi_{n}\right).$$

*Thus, ⨁ denotes the left-associated finite iteration of the derived binary operation ⊕.*

**Remark** **1.** *The primitive Sheffer stroke operation ∣ is not assumed to be associative in a general Sheffer stroke basic algebra. The derived operation*

(11) x⊕y=(x∣x)∣(y∣y)

*may be associative in particular algebras, but no global associativity of ⊕ is assumed in the present framework. Hence, differently bracketed expressions such as*

(12) (x⊕y)⊕zandx⊕(y⊕z)

*are not identified unless associativity is separately verified. For this reason, all finite Sheffer sums in this paper are evaluated according to the fixed left-recursive convention in Definition 5. Thus, the order and bracketing of a finite sum are part of the formal construction. The partition, refinement, and joint refinement statements proved below are therefore statements about these recursively evaluated expressions and are not assertions of invariance under arbitrary reassociation or reordering.*

**Definition** **6.** *A Sheffer stroke basic algebra B=(B;∣) is said to be σ-complete if every countable subset of B has both a supremum and an infimum with respect to its induced partial order ≤, where the relation ξ≤υ is natively defined by ξ∣(υ∣υ)=1.*

**Definition** **7.** *Let (B;∣) be a Sheffer stroke basic algebra with least element 0 and greatest element 1 with respect to its induced partial order. Assume that the recursive Sheffer sum ⨁ is defined as in Definition 5.*

*Two elements ξ,υ∈B are called orthogonal if ξ∣υ=1. A finite family $\upsilon_{1},\ldots ,\upsilon_{n}\in B$ is called mutually orthogonal if $\upsilon_{i}|\upsilon_{j}=1$ for all i≠j.*

*A product Sheffer stroke basic algebra is an algebraic structure B=(B;∣) satisfying the following conditions for all ξ,υ,θ∈B:*

(*i*) *(ξ∣1)∣(ξ∣1)=ξ.*
(*ii*) *(ξ∣0)∣(ξ∣0)=0.*
(*iii*)
$$\begin{array}{cc} & (\xi |\left((\upsilon |\upsilon )|(\theta |\theta )\right))|\left(\xi |\left((\upsilon |\upsilon )|(\theta |\theta )\right)\right)\\ & =(((\xi |\upsilon )|(\xi |\upsilon ))|((\xi |\upsilon )|(\xi |\upsilon )))|(\left((\xi |\theta )|(\xi |\theta )\right)|\left((\xi |\theta )|(\xi |\theta )\right)). \end{array}$$
(*iv*) *Orthogonal distributivity: For every finite sequence of mutually orthogonal elements $\upsilon_{1},\ldots ,\upsilon_{n}\in B$, the following Sheffer stroke distributive identity holds:*
$$\left(\xi |\overset{n}{\underset{j=1}{⨁}}\upsilon_{j}\right)|\left(\xi |\overset{n}{\underset{j=1}{⨁}}\upsilon_{j}\right)=\overset{n}{\underset{j=1}{⨁}}\left(\left(\xi |\upsilon_{j}\right)|\left(\xi |\upsilon_{j}\right)\right).$$
(*v*) *For all ξ,υ∈B,*
((ξ∣υ)∣(ξ∣υ))≤ξand((ξ∣υ)∣(ξ∣υ))≤υ.

**Remark** **2.** *Conditions (iii) and (iv) in Definition 7 encode the distributive behavior required for the Sheffer stroke-derived joint components used later in the paper. Informally, the term*

(13) (x∣y)∣(x∣y)

*plays the role of a derived joint component term associated with x and y. With this notation in mind, condition (iii) says that this derived term distributes over the binary derived sum*

(14) υ⊕θ=(υ∣υ)∣(θ∣θ).

*Equivalently, it is a Sheffer stroke formulation of a binary distributivity rule for the derived joint component. Condition (iv) is the finite orthogonal version of the same principle: when $\upsilon_{1},\ldots ,\upsilon_{n}$ are mutually orthogonal, the derived joint term of ξ with their recursive Sheffer sum decomposes into the recursive Sheffer sum of the individual derived joint components. Finally, condition (v) ensures that each component*

(15) (ξ∣υ)∣(ξ∣υ)

*lies below both ξ and υ in the induced order. This order-theoretic compatibility is one of the assumptions allowing these derived terms to be used as admissible joint refinement components in the subsequent entropy constructions.*

**Lemma** **5.** *Let B=(B;∣) be a product Sheffer stroke basic algebra. If a≤b, c≤d, and b∣d=1, then a∣c=1.*

**Proof.**  Since the reduct (B;∣) is a Sheffer stroke basic algebra with a least element 0 and a greatest element 1, Corollary 1 implies that its induced ordered structure is a lattice with an antitone involution. We use the standard notation $x^{*}:=x|x$ for this involution. By Lemma 3, for all x,y∈B,

(16) $$x|y=1\;⟺\;x\leq y^{*}.$$

Indeed, by the definition of the induced order, $x\leq y^{*}$ is equivalent to $x|(y^{*}|y^{*})=1$, and since ^*^ is involutive, $y^{*}|y^{*}=y$. Now, from b∣d=1 we obtain $b\leq d^{*}$. Since a≤b, it follows that $a\leq d^{*}$. Moreover, c≤d and the antitonicity of ^*^ imply $d^{*}\leq c^{*}$. Hence, $a\leq c^{*}$, and therefore a∣c=1.    □

**Lemma** **6.** *Let B=(B;∣) be a product Sheffer stroke basic algebra. If x∣x=1, then x=0.*

**Proof.**  Since the reduct (B;∣) is a Sheffer stroke basic algebra with a least element 0 and a greatest element 1, Corollary 1 implies that the induced ordered structure is a lattice with an antitone involution given by $x^{*}=x|x$. Since 0 is the least element and 1 is the greatest element, we have $0^{*}=1$. If x∣x=1, then $x^{*}=1=0^{*}$. Applying the involution to both sides gives x=0.    □

**Lemma** **7.** *Let B=(B;∣) be a product Sheffer stroke basic algebra. Deleting zero components from any finite recursively summed family does not change the value of the recursive Sheffer sum.*

**Proof.**  This follows from the neutrality of 0 for the derived operation ⊕, namely x⊕0=0⊕x=x, applied recursively to the finite Sheffer sum defined in Definition 5.    □

**Definition** **8.** *Let B=(B;∣) be a product Sheffer stroke basic algebra. A partition in B is a finite indexed family*

$$\Xi =(\xi_{1},\ldots ,\xi_{k})$$

*of pairwise distinct nonzero elements of B such that:*

(*i*) *Pairwise orthogonality: $\xi_{i}|\xi_{j}=1\;\mathrm{for}\;\mathrm{all}\;i,j\in \left\{1,\ldots ,k\right\}\;\mathrm{with}\;i\neq j;$*
(*ii*) *Completeness: ${⨁}_{i=1}^{k}\xi_{i}=1.$*

*In other words, a partition consists of mutually orthogonal nonzero components whose iterated derived sum equals the unit element.*

**Remark** **3.** *The conditions in Definition 8 are essential for obtaining well-defined entropy values on admissible partitions. In particular, the pairwise distinctness requirement prevents redundant representations of the same partition component. A finite family such as (a,b,b) is therefore not an admissible partition, because the same nonzero component would be counted more than once. Such repetitions could artificially alter the entropy values whenever, for example, $0<\tau^{R}\left(b\right)<1$. Hence, throughout the paper, a partition is required to consist of nonzero, pairwise distinct, and mutually orthogonal components whose recursive Sheffer sum is the greatest element 1.*

**Proposition** **1.** *Let B=(B;∣) be a product Sheffer stroke basic algebra. Suppose that $M=\{\xi_{1},\ldots ,\xi_{k}\}$ is a finite, mutually orthogonal family of nonzero elements in B. If M is maximal, then M constitutes a partition in the sense of Definition 8. Equivalently, its recursive Sheffer sum evaluates strictly to the greatest element, ${⨁}_{i=1}^{k}\xi_{i}=1$.*

**Proof.**  Let $S={⨁}_{i=1}^{k}\xi_{i}$ denote the recursive Sheffer sum of the family *M*, and let $x^{*}:=x|x$ represent the antitone involution established in Corollary 1.

We first demonstrate that $\xi_{i}\leq S$ for all i∈{1,…,k}. By the orthogonal distributivity axiom (Definition 7(iv)), the self-stroke of the joint term expands as

(17) $$\left(\xi_{i}|S\right)|\left(\xi_{i}|S\right)=\overset{k}{\underset{j=1}{⨁}}\left(\left(\xi_{i}|\xi_{j}\right)|\left(\xi_{i}|\xi_{j}\right)\right).$$

Due to the mutual orthogonality of *M*, $\xi_{i}|\xi_{j}=1$ for all j≠i, which yields $\left(\xi_{i}|\xi_{j}\right)|\left(\xi_{i}|\xi_{j}\right)=1|1=0$. For the diagonal term j=i, the involution property gives $\left(\xi_{i}|\xi_{i}\right)|\left(\xi_{i}|\xi_{i}\right)=\xi_{i}$. Consequently, the recursive sum in Equation (17) collapses to $0⊕\cdots ⊕\xi_{i}⊕\cdots ⊕0$. Since deleting zero terms preserves the recursive Sheffer sum (Lemma 7), this strictly reduces to $\left(\xi_{i}|S\right)|\left(\xi_{i}|S\right)=\xi_{i}$. Applying Definition 7(v), we obtain $(\left(\xi_{i}|S\right)|\left(\xi_{i}|S\right))\leq S$, thereby confirming that $\xi_{i}\leq S$.

Next, we define the orthocomplement-like element $Z:=S|S=S^{*}$, and assert that *Z* is mutually orthogonal to every member of *M*. Since $\xi_{i}\leq S$, Lemma 3 dictates that $\xi_{i}|\left(S|S\right)=1$, yielding $\xi_{i}|Z=1$. Conversely, utilizing the antitonicity of the involution (Corollary 1), $\xi_{i}\leq S$ implies $S^{*}\leq \xi_{i}^{*}$, which translates to $Z\leq \xi_{i}|\xi_{i}$. Reapplying Lemma 3, we obtain $Z|\left(\left(\xi_{i}|\xi_{i}\right)|\left(\xi_{i}|\xi_{i}\right)\right)=1$. Because the inner term evaluates back to $\xi_{i}$, it logically follows that $Z|\xi_{i}=1$. Thus, *Z* is orthogonal to every $\xi_{i}\in M$ in both directions.

We now invoke the maximality of *M*. Suppose, by contradiction, that Z≠0. If $Z=\xi_{i}$ for some index *i*, the established orthogonality $Z|\xi_{i}=1$ would imply $\xi_{i}|\xi_{i}=1$, forcing $\xi_{i}=0$ via Lemma 6 and contradicting the premise that *M* strictly comprises nonzero elements. Hence, Z∉M. However, this implies *Z* is an external nonzero element orthogonal to the entire family *M*, allowing the formation of a strictly larger mutually orthogonal family M∪{Z}. This directly violates the assumed maximality of *M*.

Therefore, we must conclude that Z=0. Substituting this back into our definition, we find $S^{*}=0$. Applying the involution once more yields $S={\left(S^{*}\right)}^{*}=0^{*}=1$, proving that ${⨁}_{i=1}^{k}\xi_{i}=1$.

Finally, the elements of *M* are inherently pairwise distinct; if $\xi_{i}=\xi_{j}$ for any i≠j, their mutual orthogonality $\xi_{i}|\xi_{j}=1$ would again necessitate $\xi_{i}|\xi_{i}=1$, leading to the contradiction $\xi_{i}=0$. As a finite, mutually orthogonal family of pairwise distinct nonzero elements summing to the greatest element 1, *M* rigorously satisfies all criteria of a partition as stipulated in Definition 8.    □

**Definition** **9.** *Let $\Xi =(\xi_{1},\ldots ,\xi_{p})$ and $\Upsilon =(\upsilon_{1},\ldots ,\upsilon_{q})$ be partitions in B=(B;∣). We say that Υ is a refinement of Ξ if there exists a family $\left(J_{1},\ldots ,J_{p}\right)$ of pairwise disjoint nonempty subsets of {1,…,q} such that*

(18) $$\overset{p}{\underset{i=1}{⋃}}J_{i}=\left\{1,\ldots ,q\right\},$$

*and, for each i∈{1,…,p},*

(19) $$\xi_{i}=\underset{j\in J_{i}}{⨁}\upsilon_{j},$$

*where the elements $\upsilon_{j}$ are summed in increasing order of their indices according to the recursive operation defined in Definition 5. In this case, we write Ξ≤Υ.*

**Proposition** **2.** *Let $\Xi =(\xi_{1},\ldots ,\xi_{p})$ and $\Upsilon =(\upsilon_{1},\ldots ,\upsilon_{q})$ be partitions in the sense of Definition 8. If*

(20) Ξ≤ΥandΥ≤Ξ,

*then p=q and there exists a permutation π of {1,…,p} such that*

$$\xi_{i}=\upsilon_{\pi (i)}\;\mathrm{for}\;\mathrm{all}\;i=1,\ldots ,p.$$

*Hence, mutually refining admissible partitions coincide up to a permutation.*

**Proof.**  Assume first that Ξ≤Υ. By Definition 9, there exists a family $\left(J_{1},\ldots ,J_{p}\right)$ of pairwise disjoint nonempty subsets of {1,…,q} such that

(21) $$\overset{p}{\underset{i=1}{⋃}}J_{i}=\left\{1,\ldots ,q\right\}$$

and

(22) $$\xi_{i}=\underset{j\in J_{i}}{⨁}\upsilon_{j}\;\mathrm{for}\;\mathrm{each}\;i=1,\ldots ,p.$$

Since the sets $J_{1},\ldots ,J_{p}$ are nonempty and pairwise disjoint, we must have q≥p.

Similarly, from Υ≤Ξ, there exists a family $\left(K_{1},\ldots ,K_{q}\right)$ of pairwise disjoint nonempty subsets of {1,…,p} such that

(23) $$\overset{q}{\underset{j=1}{⋃}}K_{j}=\left\{1,\ldots ,p\right\}$$

and

(24) $$\upsilon_{j}=\underset{i\in K_{j}}{⨁}\xi_{i}\;\mathrm{for}\;\mathrm{each}\;j=1,\ldots ,q.$$

Hence, p≥q.

Therefore, p=q. Since {1,…,q} is partitioned into exactly *q* nonempty pairwise disjoint subsets, each $J_{i}$ must be a singleton. Thus, there exists a permutation π of {1,…,p} such that

(25) $$J_{i}=\left\{\pi \left(i\right)\right\}\;\mathrm{and}\;\mathrm{hence}\;\xi_{i}=\upsilon_{\pi (i)}$$

for every i=1,…,p. This proves the claim.    □

**Definition** **10.** *Let $\Xi =(\xi_{1},\ldots ,\xi_{k})$ and $\Upsilon =(\upsilon_{1},\ldots ,\upsilon_{l})$ be two partitions in a product Sheffer stroke basic algebra B=(B;∣). The raw joint family of Ξ and Υ is an r-tuple (where r=kl) whose elements are defined as follows:*

(26) $$\zeta_{ij}=\left(\xi_{i}|\upsilon_{j}\right)|\left(\xi_{i}|\upsilon_{j}\right),\;i=1,\ldots ,k,\;j=1,\ldots ,l.$$

*The nonzero elements of this raw family form the associated Sheffer stroke joint family. This family is used as an associated joint partition only when the product Sheffer stroke assumptions, the fixed recursive Sheffer sum convention, and the required orthogonality and marginalization properties ensure that the nonzero components form a partition in the sense of Definition 8.*

*For notational convenience, whenever these admissibility requirements are satisfied, we denote the associated joint partition by*

(27) (Ξ∣Υ)∣(Ξ∣Υ).

*This notation denotes the componentwise Sheffer stroke joint construction and should not be confused with an unrestricted lattice join of arbitrary partitions.*

**Lemma** **8.** *Let B=(B;∣) be a product Sheffer stroke basic algebra. For all ξ,υ∈B, define*

(28) ζ=(ξ∣υ)∣(ξ∣υ).

*Then*

(29) ζ≤ξandζ≤υ.

**Proof.**  This follows directly from Definition 7(v).    □

**Proposition** **3.** *Let $\Xi =(\xi_{1},\ldots ,\xi_{k})$ and $\Upsilon =(\upsilon_{1},\ldots ,\upsilon_{l})$ be partitions in a product Sheffer stroke basic algebra B=(B;∣). Under the product assumptions of Definition 7 and the fixed recursive Sheffer sum convention, the associated Sheffer stroke joint family obtained from the nonzero components of*

(30) $$\zeta_{ij}=\left(\xi_{i}|\upsilon_{j}\right)|\left(\xi_{i}|\upsilon_{j}\right)$$

*is a partition in the sense of Definition 8. Its components are algebraic joint refinement components associated with Ξ and Υ; no operational joint-measurement interpretation is assumed.*

**Proof.**  For each i=1,…,k and j=1,…,l, put

(31) $$\zeta_{ij}=\left(\xi_{i}|\upsilon_{j}\right)|\left(\xi_{i}|\upsilon_{j}\right).$$

All finite sums below are evaluated according to the fixed recursive convention of Definition 5. We collect all nonzero elements from the family $\left\{\zeta_{ij}\right\}$. We show that these elements are mutually orthogonal, pairwise distinct, and have recursive Sheffer sum equal to 1.

Let $\left(i,j\right)\neq \left(i^{\prime },j^{\prime }\right)$. If $i\neq i^{\prime }$, then the orthogonality of Ξ gives $\xi_{i}|\xi_{i^{\prime }}=1$. By Lemma 8,

(32) $$\zeta_{ij}\leq \xi_{i}\;\mathrm{and}\;\zeta_{i^{\prime }j^{\prime }}\leq \xi_{i^{\prime }}.$$

Hence, Lemma 5 yields

(33) $$\zeta_{ij}|\zeta_{i^{\prime }j^{\prime }}=1.$$

If $j\neq j^{\prime }$, then the orthogonality of Υ gives $\upsilon_{j}|\upsilon_{j^{\prime }}=1$. Again by Lemma 8,

(34) $$\zeta_{ij}\leq \upsilon_{j}\;\mathrm{and}\;\zeta_{i^{\prime }j^{\prime }}\leq \upsilon_{j^{\prime }}.$$

Thus Lemma 5 gives

(35) $$\zeta_{ij}|\zeta_{i^{\prime }j^{\prime }}=1.$$

Therefore, the nonzero joint components are mutually orthogonal.

Next, suppose that $\zeta_{ij}=\zeta_{i^{\prime }j^{\prime }}=x$ for two distinct index pairs. Since the corresponding components are mutually orthogonal, x∣x=1. By Lemma 6, this implies x=0. Hence, no two nonzero joint components can coincide.

It remains to prove completeness. By the orthogonal distributivity in Definition 7(iv), for each fixed *i*,

(36) $$\overset{l}{\underset{j=1}{⨁}}\zeta_{ij}=\overset{l}{\underset{j=1}{⨁}}\left(\left(\xi_{i}|\upsilon_{j}\right)|\left(\xi_{i}|\upsilon_{j}\right)\right)=\left(\xi_{i}|\overset{l}{\underset{j=1}{⨁}}\upsilon_{j}\right)|\left(\xi_{i}|\overset{l}{\underset{j=1}{⨁}}\upsilon_{j}\right).$$

Since Υ is a partition, ${⨁}_{j=1}^{l}\upsilon_{j}=1$. Hence, by Definition 7(i),

(37) $$\overset{l}{\underset{j=1}{⨁}}\zeta_{ij}=\left(\xi_{i}|1\right)|\left(\xi_{i}|1\right)=\xi_{i}.$$

Taking the recursive sum over i=1,…,k, we obtain

(38) $$\overset{k}{\underset{i=1}{⨁}}\overset{l}{\underset{j=1}{⨁}}\zeta_{ij}=\overset{k}{\underset{i=1}{⨁}}\xi_{i}=1.$$

Finally, by Lemma 7, deleting zero components does not change the value of the recursive Sheffer sum. Therefore, the associated nonzero joint family has recursive Sheffer sum equal to 1.

Consequently, the associated joint family consists of pairwise distinct, mutually orthogonal, nonzero elements whose recursive Sheffer sum is 1. Hence, it is a partition in the sense of Definition 8.    □

**Definition** **11.** *Let $\Xi =(\xi_{1},\ldots ,\xi_{k})$ be a partition in a product Sheffer stroke basic algebra B=(B;∣), and let $\tau^{R}:B\to \left[0,1\right]$ be a Riečan state on B. The entropy of Ξ with respect to $\tau^{R}$ is defined by*

(39) $$H_{\tau^{R}}\left(\Xi \right)=-\sum_{i=1}^{k}\tau^{R}\left(\xi_{i}\right)\log\tau^{R}\left(\xi_{i}\right).$$

*If $\Xi =(\xi_{1},\ldots ,\xi_{k})$ and $\Upsilon =(\upsilon_{1},\ldots ,\upsilon_{l})$ are two partitions in B=(B;∣), the conditional entropy of Ξ given an element $\upsilon_{j}\in \Upsilon$ is defined by*

(40) $$H_{\tau^{R}}\left(\Xi /\upsilon_{j}\right)=-\sum_{i=1}^{k}\tau^{R}\left(\xi_{i}/\upsilon_{j}\right)\log\tau^{R}\left(\xi_{i}/\upsilon_{j}\right),$$

*where $\tau^{R}\left(\xi_{i}/\upsilon_{j}\right)$ is given by*

(41) $$\tau^{R}\left(\xi_{i}/\upsilon_{j}\right)=\left\{\begin{array}{cc} \frac{\tau^{R}\left(\left(\xi_{i}|\upsilon_{j}\right)|\left(\xi_{i}|\upsilon_{j}\right)\right)}{\tau^{R}\left(\upsilon_{j}\right)}, & \mathrm{if}\;\tau^{R}\left(\upsilon_{j}\right)>0;\\ 0, & \mathrm{if}\;\tau^{R}\left(\upsilon_{j}\right)=0. \end{array}\right.$$

*Furthermore, the conditional entropy of Ξ given the partition Υ is defined as the $\tau^{R}$-weighted sum of the individual conditional entropies:*

(42) $$\begin{array}{cc} H_{\tau^{R}}\left(\Xi /\Upsilon \right) & =\sum_{j=1}^{l}\tau^{R}\left(\upsilon_{j}\right)H_{\tau^{R}}\left(\Xi /\upsilon_{j}\right)\\ \; & =-\sum_{i=1}^{k}\sum_{j=1}^{l}\tau^{R}\left(\left(\xi_{i}|\upsilon_{j}\right)|\left(\xi_{i}|\upsilon_{j}\right)\right)\log\left(\frac{\tau^{R}\left(\left(\xi_{i}|\upsilon_{j}\right)|\left(\xi_{i}|\upsilon_{j}\right)\right)}{\tau^{R}\left(\upsilon_{j}\right)}\right). \end{array}$$

**Remark** **4.** *In Definition 11, we adopt the standard information-theoretic convention that 0log0=0. Furthermore, if $\tau^{R}\left(\upsilon_{j}\right)=0$, it implies that $\tau^{R}\left(\left(\xi_{i}|\upsilon_{j}\right)|\left(\xi_{i}|\upsilon_{j}\right)\right)=0$ (due to monotonicity, since $\left(\xi_{i}|\upsilon_{j}\right)|\left(\xi_{i}|\upsilon_{j}\right)\leq \upsilon_{j}$); in such cases, the corresponding summand is taken to be 0. Note that to prevent any notational ambiguity with the Sheffer stroke operation (∣), we deliberately use the slash symbol (/) to denote conditional states and conditional entropy (e.g., $H_{\tau^{R}}\left(\Xi /\Upsilon \right)$ instead of the traditional $H_{\tau^{R}}\left(\Xi |\Upsilon \right)$). Finally, the base of the logarithm can be any real number a>1. Typically, logarithms to the base 2 are used, measuring the entropy in bits, whereas natural logarithms express the entropy in nats.*

To handle this definition algorithmically, we construct the following pseudocode:

Algorithm 1 outlines the computational procedure required to evaluate the entropy and conditional entropy of partitions within a product Sheffer stroke basic algebra. The algorithm accepts the algebraic structure B=(B;∣), two finite partitions Ξ and Υ, and a Riečan state operator $\tau^{R}:B\to \left[0,1\right]$ as inputs. It returns the scalar values of the entropy $H_{\tau^{R}}\left(\Xi \right)$ and the conditional entropy $H_{\tau^{R}}\left(\Xi /\Upsilon \right)$.
**Algorithm 1:** Calculation of Sheffer Stroke Entropy and Conditional Entropy**Input:** Product Sheffer stroke basic algebra B=(B;∣), partition $\Xi =(\xi_{1},\ldots ,\xi_{k})$, partition $\Upsilon =(\upsilon_{1},\ldots ,\upsilon_{l})$, Riečan state operator $\tau^{R}:B\to \left[0,1\right]$**Output**: Entropy of Ξ, $H_{\tau^{R}}\left(\Xi \right)$; Conditional entropy of Ξ given Υ, $H_{\tau^{R}}\left(\Xi /\Upsilon \right)$// Computational convention: terms of the form 0log0 are evaluated as 0.(// Therefore, logarithmic contributions are accumulated only for strictly positive state values.
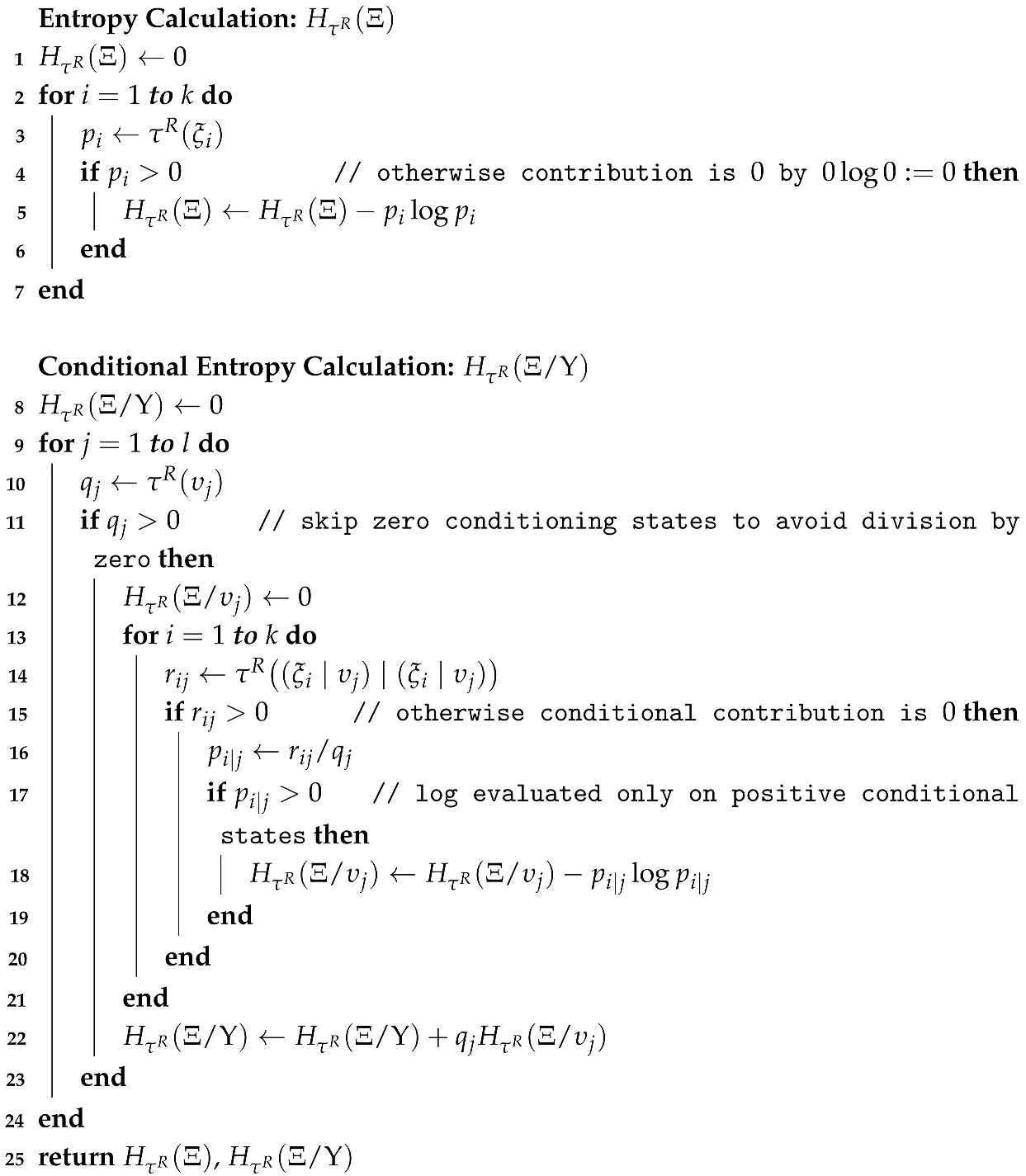


    The pseudocode explicitly implements the computational convention 0log0:=0. For this reason, logarithmic contributions are accumulated only for strictly positive state values. Zero-state terms are skipped since their entropy contribution is defined to be zero. In the conditional part, conditioning elements with $\tau^{R}\left(\upsilon_{j}\right)=0$ are also skipped to avoid division by zero; this is consistent with the convention used in the definition of conditional states.

The computational process is structured into two sequential phases. The first phase evaluates the marginal entropy of the primary partition Ξ. After initializing the global entropy accumulator to zero, the algorithm iterates through each element $\xi_{i}\in \Xi$ and stores its state value as $p_{i}=\tau^{R}\left(\xi_{i}\right)$. If $p_{i}>0$, the term $-p_{i}\log p_{i}$ is added to the entropy accumulator; otherwise, the contribution is omitted in accordance with 0log0:=0.

The second phase calculates the conditional entropy of Ξ given Υ. For each conditioning element $\upsilon_{j}$, the algorithm first computes $q_{j}=\tau^{R}\left(\upsilon_{j}\right)$. If $q_{j}=0$, the corresponding conditional branch is skipped. If $q_{j}>0$, the algorithm evaluates the joint state values

$$r_{ij}=\tau^{R}\left(\left(\xi_{i}|\upsilon_{j}\right)|\left(\xi_{i}|\upsilon_{j}\right)\right)$$

and forms the conditional ratios $p_{i|j}=r_{ij}/q_{j}$ only when $r_{ij}>0$. The local conditional entropy $H_{\tau^{R}}\left(\Xi /\upsilon_{j}\right)$ is then accumulated from the positive contributions $-p_{i|j}\log p_{i|j}$ and weighted by $q_{j}$ in the final sum. This implementation is numerically stable and directly mirrors the theoretical convention used in the entropy definitions.

**Proposition** **4.** *Let $\Xi =(\xi_{1},\ldots ,\xi_{k})$ and $\Upsilon =(\upsilon_{1},\ldots ,\upsilon_{l})$ be partitions in a product Sheffer stroke basic algebra B=(B;∣), and let $\tau^{R}:B\to \left[0,1\right]$ be a Riečan state. Then, for the associated admissible Sheffer stroke joint partition satisfying the marginalization identities induced by Proposition 6, the entropy satisfies the subadditivity property:*

(43) $$H_{\tau^{R}}\left((\Xi |\Upsilon )|(\Xi |\Upsilon )\right)\leq H_{\tau^{R}}\left(\Xi \right)+H_{\tau^{R}}\left(\Upsilon \right).$$

**Proof.**  The proof applies Jensen’s inequality to the convex function u(x)=xlogx after expressing each marginal state value $\tau^{R}\left(\xi_{i}\right)$ as a sum of joint refinement state values over Υ. The resulting inequality is then rewritten using the marginalization identities of the Sheffer stroke joint refinement to obtain the desired subadditivity estimate.

Define the continuous function u:[0,∞)→R by

(44) u(x)=xlogx,

with the standard convention u(0)=0. Assuming the logarithm is taken with base a>1, its second derivative is

(45) $$u^{\prime \prime }\left(x\right)=\frac{1}{x\ln a}>0\;\mathrm{for}\;\mathrm{all}\;x>0.$$

Hence, *u* is strictly convex on (0,∞) and, by continuity, convex on [0,∞).

Fix i∈{1,…,k}, and for each index *j* satisfying $\tau^{R}\left(\upsilon_{j}\right)>0$, define

(46) $$a_{j}=\tau^{R}\left(\upsilon_{j}\right),\;x_{j}=\frac{\tau^{R}\left(\left(\xi_{i}|\upsilon_{j}\right)|\left(\xi_{i}|\upsilon_{j}\right)\right)}{\tau^{R}\left(\upsilon_{j}\right)}.$$

Since Υ is a partition, we have ${⨁}_{j=1}^{l}\upsilon_{j}=1$, and therefore

(47) $$\sum_{j=1}^{l}\tau^{R}\left(\upsilon_{j}\right)=1.$$

Moreover, for all indices with $\tau^{R}\left(\upsilon_{j}\right)>0$, we have $a_{j}>0$, and the zero-probability terms contribute nothing. Hence,

(48) $$\sum_{a_{j}>0}a_{j}=1.$$

Thus, the coefficients $a_{j}$ form a normalized system of Jensen weights.

Utilizing the distributivity of the derived product over the Sheffer sum and the additivity of the Riečan state over orthogonal elements, the marginal state $\tau^{R}\left(\xi_{i}\right)$ can be expanded as

(49) $$\tau^{R}\left(\xi_{i}\right)=\tau^{R}\left((\xi_{i}|\overset{l}{\underset{j=1}{⨁}}\upsilon_{j})|(\xi_{i}|\overset{l}{\underset{j=1}{⨁}}\upsilon_{j})\right)=\sum_{j=1}^{l}\tau^{R}\left(\left(\xi_{i}|\upsilon_{j}\right)|\left(\xi_{i}|\upsilon_{j}\right)\right).$$

Due to monotonicity, if $\tau^{R}\left(\upsilon_{j}\right)=0$, then

(50) $$\tau^{R}\left(\left(\xi_{i}|\upsilon_{j}\right)|\left(\xi_{i}|\upsilon_{j}\right)\right)=0.$$

Therefore, restricting the sum to indices with $a_{j}>0$ does not change its value. Applying Jensen’s inequality to the convex function *u*, we obtain

(51) $$\begin{array}{cc} u(\tau^{R}\left(\xi_{i}\right))\; & =u\left(\sum_{a_{j}>0}\tau^{R}\left(\left(\xi_{i}|\upsilon_{j}\right)|\left(\xi_{i}|\upsilon_{j}\right)\right)\right)\\ \; & =u\left(\sum_{a_{j}>0}\tau^{R}\left(\upsilon_{j}\right)\frac{\tau^{R}\left(\left(\xi_{i}|\upsilon_{j}\right)|\left(\xi_{i}|\upsilon_{j}\right)\right)}{\tau^{R}\left(\upsilon_{j}\right)}\right)\\ \; & =u\left(\sum_{a_{j}>0}a_{j}x_{j}\right)\\ \; & \leq \sum_{a_{j}>0}a_{j}u\left(x_{j}\right)\\ \; & =\sum_{a_{j}>0}\tau^{R}\left(\upsilon_{j}\right)\;u\left(\frac{\tau^{R}\left(\left(\xi_{i}|\upsilon_{j}\right)|\left(\xi_{i}|\upsilon_{j}\right)\right)}{\tau^{R}\left(\upsilon_{j}\right)}\right). \end{array}$$

Reintroducing the zero-probability terms allows us to sum over all j=1,…,l. Using the explicit form of *u*, we get

(52) $$a_{j}u\left(x_{j}\right)=\tau^{R}\left(\left(\xi_{i}|\upsilon_{j}\right)|\left(\xi_{i}|\upsilon_{j}\right)\right)\log\left(\frac{\tau^{R}\left(\left(\xi_{i}|\upsilon_{j}\right)|\left(\xi_{i}|\upsilon_{j}\right)\right)}{\tau^{R}\left(\upsilon_{j}\right)}\right).$$

Summing over all i=1,…,k and multiplying by −1, we obtain

(53) $$\begin{array}{cc} H_{\tau^{R}}\left(\Xi \right)\; & =-\sum_{i=1}^{k}u\left(\tau^{R}\left(\xi_{i}\right)\right)\\ \; & \geq -\sum_{i=1}^{k}\sum_{j=1}^{l}\tau^{R}\left(\upsilon_{j}\right)\;u\left(\frac{\tau^{R}\left(\left(\xi_{i}|\upsilon_{j}\right)|\left(\xi_{i}|\upsilon_{j}\right)\right)}{\tau^{R}\left(\upsilon_{j}\right)}\right)\\ \; & =-\sum_{i=1}^{k}\sum_{j=1}^{l}\tau^{R}\left(\left(\xi_{i}|\upsilon_{j}\right)|\left(\xi_{i}|\upsilon_{j}\right)\right)\log\left(\frac{\tau^{R}\left(\left(\xi_{i}|\upsilon_{j}\right)|\left(\xi_{i}|\upsilon_{j}\right)\right)}{\tau^{R}\left(\upsilon_{j}\right)}\right)\\ \; & =-\sum_{i=1}^{k}\sum_{j=1}^{l}\tau^{R}\left(\left(\xi_{i}|\upsilon_{j}\right)|\left(\xi_{i}|\upsilon_{j}\right)\right)\left[\log\tau^{R}\left(\left(\xi_{i}|\upsilon_{j}\right)|\left(\xi_{i}|\upsilon_{j}\right)\right)-\log\tau^{R}\left(\upsilon_{j}\right)\right]\\ \; & =-\sum_{i=1}^{k}\sum_{j=1}^{l}\tau^{R}\left(\left(\xi_{i}|\upsilon_{j}\right)|\left(\xi_{i}|\upsilon_{j}\right)\right)\log\tau^{R}\left(\left(\xi_{i}|\upsilon_{j}\right)|\left(\xi_{i}|\upsilon_{j}\right)\right)\\ \; & \;-\left(-\sum_{j=1}^{l}\left(\sum_{i=1}^{k}\tau^{R}\left(\left(\xi_{i}|\upsilon_{j}\right)|\left(\xi_{i}|\upsilon_{j}\right)\right)\right)\log\tau^{R}\left(\upsilon_{j}\right)\right). \end{array}$$

Recognizing that

(54) $$\sum_{i=1}^{k}\tau^{R}\left(\left(\xi_{i}|\upsilon_{j}\right)|\left(\xi_{i}|\upsilon_{j}\right)\right)=\tau^{R}\left(\upsilon_{j}\right),$$

the first term on the right-hand side is exactly the joint entropy $H_{\tau^{R}}\left((\Xi |\Upsilon )|(\Xi |\Upsilon )\right)$, and the second term is $H_{\tau^{R}}\left(\Upsilon \right)$. Therefore,

(55) $$H_{\tau^{R}}\left(\Xi \right)\geq H_{\tau^{R}}\left((\Xi |\Upsilon )|(\Xi |\Upsilon )\right)-H_{\tau^{R}}\left(\Upsilon \right).$$

Rearranging the terms yields

(56) $$H_{\tau^{R}}\left((\Xi |\Upsilon )|(\Xi |\Upsilon )\right)\leq H_{\tau^{R}}\left(\Xi \right)+H_{\tau^{R}}\left(\Upsilon \right).$$

   □

**Definition** **12.** *Let $\Xi =(\xi_{1},\ldots ,\xi_{k})$ be a partition in a product Sheffer stroke basic algebra B=(B;∣), and let $\tau^{R}:B\to \left[0,1\right]$ be a Riečan state on B. The logical entropy of Ξ with respect to $\tau^{R}$ is defined by*

(57) $$H_{\tau^{R}}^{l}\left(\Xi \right)=\sum_{i=1}^{k}l\left(\tau^{R}\left(\xi_{i}\right)\right),$$

*where l:[0,1]→[0,∞) is the logical entropy function defined natively as $l\left(x\right)=x-x^{2}$.*

*If $\Xi =(\xi_{1},\ldots ,\xi_{k})$ and $\Upsilon =(\upsilon_{1},\ldots ,\upsilon_{l})$ are two partitions in B=(B;∣), the conditional logical entropy of Ξ given the partition Υ is defined by*

(58) $$H_{\tau^{R}}^{l}\left(\Xi /\Upsilon \right)=\sum_{j=1}^{l}\tau^{R}{\left(\upsilon_{j}\right)}^{2}-\sum_{i=1}^{k}\sum_{j=1}^{l}\tau^{R}{\left(\left(\xi_{i}|\upsilon_{j}\right)|\left(\xi_{i}|\upsilon_{j}\right)\right)}^{2}.$$

To translate the theoretical framework established in Definition 12 into a computational model, we construct the following algorithmic procedure:

Algorithm 2 delineates the computational methodology required to evaluate both the marginal and conditional logical entropies of partitions within a product Sheffer stroke basic algebra. The algorithm accepts the algebraic structure B=(B;∣), two finite partitions Ξ and Υ, a Riečan state operator $\tau^{R}$, and the logical entropy function l(x) as its primary inputs. It yields the scalar values for the logical entropy $H_{\tau^{R}}^{l}\left(\Xi \right)$ and the conditional logical entropy $H_{\tau^{R}}^{l}\left(\Xi /\Upsilon \right)$.   
**Algorithm 2:** Calculation of Logical Entropy and Conditional Logical Entropy**Input**: Product Sheffer stroke basic algebra B=(B;∣), partition $\Xi =(\xi_{1},\ldots ,\xi_{k})$, partition $\Upsilon =(\upsilon_{1},\ldots ,\upsilon_{l})$, Riečan state operator $\tau^{R}:B\to \left[0,1\right]$, logical entropy function l:[0,1]→[0,∞)**Output**: Logical entropy of Ξ, $H_{\tau^{R}}^{l}\left(\Xi \right)$; Conditional logical entropy of Ξ given Υ, $H_{\tau^{R}}^{l}\left(\Xi /\Upsilon \right)$// No logarithmic term occurs in logical entropy; hence the convention 0log0:=0 is not invoked here. Zero-state values are evaluated directly through l(0)=0 and squared-state terms.(
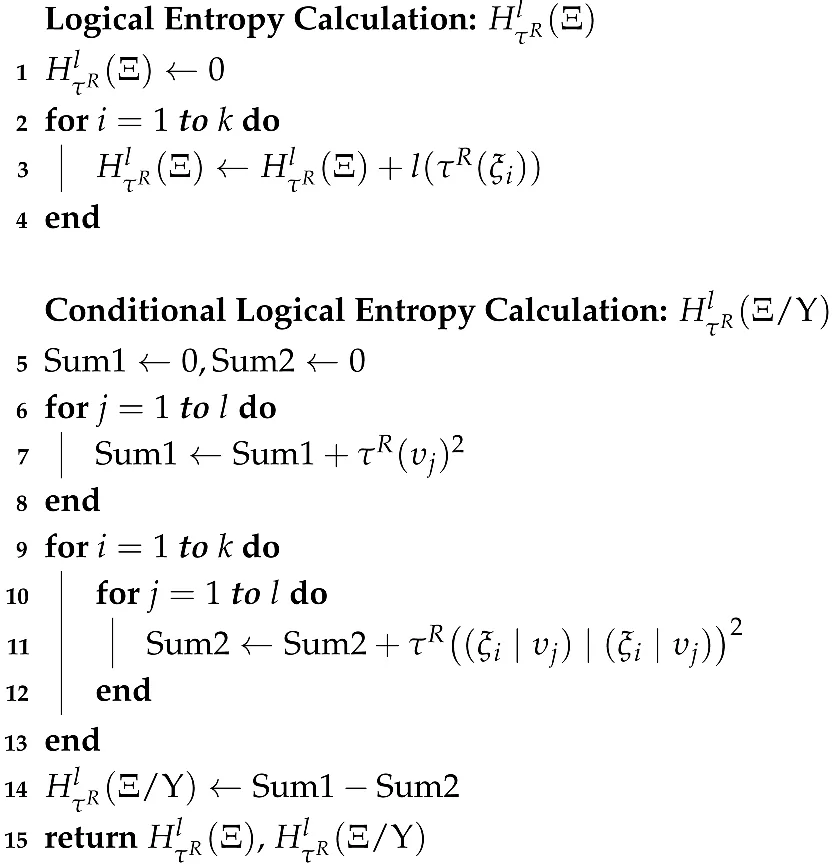


    Unlike Algorithm 1, Algorithm 2 contains no logarithmic evaluation. Therefore, the convention 0log0:=0 is not computationally invoked in the logical entropy calculation. Zero-state values are handled directly through the polynomial expression $l\left(x\right)=x-x^{2}$, for which l(0)=0, and through squared-state terms in the conditional logical entropy formula.

The computational procedure is structurally divided into two phases. The first phase calculates the marginal logical entropy of the primary partition Ξ. After initializing the principal accumulator to zero, the algorithm iterates through each element $\xi_{i}\in \Xi$. For each element, the evaluated state probability $\tau^{R}\left(\xi_{i}\right)$ is mapped through the logical entropy function l(·). The resulting value is aggregated into the total sum, mirroring the theoretical summation formula.

The second phase computes the conditional logical entropy $H_{\tau^{R}}^{l}\left(\Xi /\Upsilon \right)$ by employing two separate accumulators, which streamlines the computational complexity. The first accumulator, denoted as Sum1, independently aggregates the squared state probabilities of the conditioning partition Υ. Subsequently, the second accumulator, Sum2, utilizes a nested iteration to compute the squared joint state probabilities $\tau^{R}{\left(\left(\xi_{i}|\upsilon_{j}\right)|\left(\xi_{i}|\upsilon_{j}\right)\right)}^{2}$ across all combinations of the elements from both partitions. Finally, the conditional logical entropy is derived by subtracting the joint aggregate Sum2 from the marginal conditioning aggregate Sum1. This algorithm returns precise theoretical measurements for further algebraic analysis.

**Proposition** **5.** *Let $\Xi =(\xi_{1},\ldots ,\xi_{k})$ and $\Upsilon =(\upsilon_{1},\ldots ,\upsilon_{l})$ be partitions in a product Sheffer stroke basic algebra B=(B;∣), and let $\tau^{R}:B\to \left[0,1\right]$ be a Riečan state. Then, for the associated admissible Sheffer stroke joint partition satisfying the corresponding marginalization identities, the logical entropy satisfies the subadditivity property:*

(59) $$H_{\tau^{R}}^{l}\left((\Xi |\Upsilon )|(\Xi |\Upsilon )\right)\leq H_{\tau^{R}}^{l}\left(\Xi \right)+H_{\tau^{R}}^{l}\left(\Upsilon \right).$$

**Proof.**  The proof rewrites the logical entropy of each partition in the normalized form $H_{\tau^{R}}^{l}\left(\Xi \right)=1-\sum_{i}\tau^{R}{\left(\xi_{i}\right)}^{2}$. We then compare the sum of the two marginal logical entropies with the logical entropy of the Sheffer stroke joint refinement and show that the resulting difference is non-negative by using the marginalization identities of the joint refinement.

By Definition 12, the logical entropy function is given by $l\left(x\right)=x-x^{2}$. Since Ξ is a partition, we have ${⨁}_{i=1}^{k}\xi_{i}=1$, which implies $\sum_{i=1}^{k}\tau^{R}\left(\xi_{i}\right)=1$. Consequently, the logical entropy of Ξ can be rewritten as

(60) $$\begin{array}{cc} H_{\tau^{R}}^{l}\left(\Xi \right) & =\sum_{i=1}^{k}\left(\tau^{R}\left(\xi_{i}\right)-\tau^{R}{\left(\xi_{i}\right)}^{2}\right)\\ \; & =\sum_{i=1}^{k}\tau^{R}\left(\xi_{i}\right)-\sum_{i=1}^{k}\tau^{R}{\left(\xi_{i}\right)}^{2}\\ \; & =1-\sum_{i=1}^{k}\tau^{R}{\left(\xi_{i}\right)}^{2}. \end{array}$$

Similarly, for the partition Υ and the joint partition, we have

(61) $$H_{\tau^{R}}^{l}\left(\Upsilon \right)=1-\sum_{j=1}^{l}\tau^{R}{\left(\upsilon_{j}\right)}^{2}\;\mathrm{and}\;H_{\tau^{R}}^{l}\left((\Xi |\Upsilon )|(\Xi |\Upsilon )\right)=1-\sum_{i=1}^{k}\sum_{j=1}^{l}\tau^{R}{\left(\left(\xi_{i}|\upsilon_{j}\right)|\left(\xi_{i}|\upsilon_{j}\right)\right)}^{2}.$$

Consider the difference between the sum of the marginal entropies and the joint entropy:

(62) $$\Delta =H_{\tau^{R}}^{l}\left(\Xi \right)+H_{\tau^{R}}^{l}\left(\Upsilon \right)-H_{\tau^{R}}^{l}\left((\Xi |\Upsilon )|(\Xi |\Upsilon )\right).$$

Substituting the algebraic forms into this difference, we obtain

(63) $$\begin{array}{cc} \Delta & =\left(1-\sum_{i=1}^{k}\tau^{R}{\left(\xi_{i}\right)}^{2}\right)+\left(1-\sum_{j=1}^{l}\tau^{R}{\left(\upsilon_{j}\right)}^{2}\right)-\left(1-\sum_{i=1}^{k}\sum_{j=1}^{l}\tau^{R}{\left(\left(\xi_{i}|\upsilon_{j}\right)|\left(\xi_{i}|\upsilon_{j}\right)\right)}^{2}\right)\\ \; & =1-\sum_{i=1}^{k}\tau^{R}{\left(\xi_{i}\right)}^{2}-\sum_{j=1}^{l}\tau^{R}{\left(\upsilon_{j}\right)}^{2}+\sum_{i=1}^{k}\sum_{j=1}^{l}\tau^{R}{\left(\left(\xi_{i}|\upsilon_{j}\right)|\left(\xi_{i}|\upsilon_{j}\right)\right)}^{2}. \end{array}$$

Utilizing the distributivity of the derived product over the Sheffer sum and the additivity of the Riečan state, we express the marginal states in terms of the joint states:

$$\tau^{R}\left(\xi_{i}\right)=\sum_{j=1}^{l}\tau^{R}\left(\left(\xi_{i}|\upsilon_{j}\right)|\left(\xi_{i}|\upsilon_{j}\right)\right)\;\mathrm{and}\;\tau^{R}\left(\upsilon_{j}\right)=\sum_{i=1}^{k}\tau^{R}\left(\left(\xi_{i}|\upsilon_{j}\right)|\left(\xi_{i}|\upsilon_{j}\right)\right).$$

Furthermore, the sum of all joint states evaluates to 1. We rewrite the squared marginal terms by distributing them over the joint states:

(64) $$\begin{array}{cc} \sum_{i=1}^{k}\tau^{R}{\left(\xi_{i}\right)}^{2} & =\sum_{i=1}^{k}\tau^{R}\left(\xi_{i}\right)\left(\sum_{j=1}^{l}\tau^{R}\left(\left(\xi_{i}|\upsilon_{j}\right)|\left(\xi_{i}|\upsilon_{j}\right)\right)\right)=\sum_{i=1}^{k}\sum_{j=1}^{l}\tau^{R}\left(\left(\xi_{i}|\upsilon_{j}\right)|\left(\xi_{i}|\upsilon_{j}\right)\right)\tau^{R}\left(\xi_{i}\right), \end{array}$$

and

(65) $$\begin{array}{cc} \sum_{j=1}^{l}\tau^{R}{\left(\upsilon_{j}\right)}^{2} & =\sum_{j=1}^{l}\tau^{R}\left(\upsilon_{j}\right)\left(\sum_{i=1}^{k}\tau^{R}\left(\left(\xi_{i}|\upsilon_{j}\right)|\left(\xi_{i}|\upsilon_{j}\right)\right)\right)=\sum_{i=1}^{k}\sum_{j=1}^{l}\tau^{R}\left(\left(\xi_{i}|\upsilon_{j}\right)|\left(\xi_{i}|\upsilon_{j}\right)\right)\tau^{R}\left(\upsilon_{j}\right). \end{array}$$

Substituting these expansions into the equation for Δ and factoring out the common term yields

(66) $$\Delta =\sum_{i=1}^{k}\sum_{j=1}^{l}\tau^{R}\left(\left(\xi_{i}|\upsilon_{j}\right)|\left(\xi_{i}|\upsilon_{j}\right)\right)\left[1-\tau^{R}\left(\xi_{i}\right)-\tau^{R}\left(\upsilon_{j}\right)+\tau^{R}\left(\left(\xi_{i}|\upsilon_{j}\right)|\left(\xi_{i}|\upsilon_{j}\right)\right)\right].$$

Analyzing the expression within the brackets, we expand $\tau^{R}\left(\xi_{i}\right)+\tau^{R}\left(\upsilon_{j}\right)-\tau^{R}\left(\left(\xi_{i}|\upsilon_{j}\right)|\left(\xi_{i}|\upsilon_{j}\right)\right)$ as follows:

(67) $$\begin{array}{cc} \tau^{R}\left(\xi_{i}\right) & +\tau^{R}\left(\upsilon_{j}\right)-\tau^{R}\left(\left(\xi_{i}|\upsilon_{j}\right)|\left(\xi_{i}|\upsilon_{j}\right)\right)\\ \; & =\left(\sum_{m=1}^{l}\tau^{R}\left(\left(\xi_{i}|\upsilon_{m}\right)|\left(\xi_{i}|\upsilon_{m}\right)\right)\right)+\left(\sum_{n=1}^{k}\tau^{R}\left(\left(\xi_{n}|\upsilon_{j}\right)|\left(\xi_{n}|\upsilon_{j}\right)\right)\right)\\ \; & \;-\tau^{R}\left(\left(\xi_{i}|\upsilon_{j}\right)|\left(\xi_{i}|\upsilon_{j}\right)\right)\\ \; & =\tau^{R}\left(\left(\xi_{i}|\upsilon_{j}\right)|\left(\xi_{i}|\upsilon_{j}\right)\right)+\sum_{m\neq j}\tau^{R}\left(\left(\xi_{i}|\upsilon_{m}\right)|\left(\xi_{i}|\upsilon_{m}\right)\right)\\ \; & \;+\sum_{n\neq i}\tau^{R}\left(\left(\xi_{n}|\upsilon_{j}\right)|\left(\xi_{n}|\upsilon_{j}\right)\right). \end{array}$$

This sum represents the probability of the union of the *i*-th row and the *j*-th column in the joint partition. Since the sum of all elements in the joint partition is 1, and $\tau^{R}\left(x\right)\geq 0$ for all x∈B, it follows that

(68) $$\tau^{R}\left(\xi_{i}\right)+\tau^{R}\left(\upsilon_{j}\right)-\tau^{R}\left(\left(\xi_{i}|\upsilon_{j}\right)|\left(\xi_{i}|\upsilon_{j}\right)\right)\leq 1.$$

Therefore, the term inside the brackets in Equation (66) is non-negative:

(69) $$1-\left(\tau^{R}\left(\xi_{i}\right)+\tau^{R}\left(\upsilon_{j}\right)-\tau^{R}\left(\left(\xi_{i}|\upsilon_{j}\right)|\left(\xi_{i}|\upsilon_{j}\right)\right)\right)\geq 0.$$

Since $\tau^{R}\left(\left(\xi_{i}|\upsilon_{j}\right)|\left(\xi_{i}|\upsilon_{j}\right)\right)\geq 0$, every individual summand in Δ is the product of two non-negative real numbers. Consequently, Δ≥0.

Since $\Delta =H_{\tau^{R}}^{l}\left(\Xi \right)+H_{\tau^{R}}^{l}\left(\Upsilon \right)-H_{\tau^{R}}^{l}\left((\Xi |\Upsilon )|(\Xi |\Upsilon )\right)\geq 0$, rearranging the inequality yields

(70) $$H_{\tau^{R}}^{l}\left((\Xi |\Upsilon )|(\Xi |\Upsilon )\right)\leq H_{\tau^{R}}^{l}\left(\Xi \right)+H_{\tau^{R}}^{l}\left(\Upsilon \right).$$

   □

## 4. The Tsallis Entropy of Partitions in a Product Sheffer Stroke Basic Algebra

Having established the classical Shannon and logical entropy measures within the non-distributive framework of product Sheffer stroke basic algebras, we now introduce a parametric generalization via the Tsallis entropy functional. In this section, we define the unconditional and conditional Tsallis entropies of finite partitions natively over the basic algebra, leveraging the mathematical properties of the Riečan state operator. We formally derive the analytical properties of this framework, including bounding inequalities, state concavity, monotonicity, and conditional chain-type identities under the relevant joint refinement marginalization assumptions. Most notably, we establish the generalized subadditivity and prove that under statistical independence, the Tsallis entropy inherently exhibits a pseudo-additive relation characterized by the entropic index α. Through explicit limits, we demonstrate the smooth convergence of this parametric structure to classical Shannon mechanics. Finally, we formulate the Tsallis mutual information and discuss its pseudo-additive residual as a state-dependent algebraic indicator of non-factorizing joint statistics within the present product Sheffer stroke framework, with possible quantum-logical connections treated only at an interpretive level.

**Definition** **13.** *Let $\Xi =(\xi_{1},\ldots ,\xi_{k})$ be a partition in a product Sheffer stroke basic algebra B=(B;∣). Then, we define the Tsallis entropy of order α, where α∈(0,1)∪(1,∞), of the partition Ξ with respect to the Riečan state $\tau^{R}:B\to \left[0,1\right]$ by*

(71) $$T_{\tau^{R}}^{\alpha }\left(\Xi \right)=\frac{1}{\alpha -1}\left(1-\sum_{i=1}^{k}\tau^{R}{\left(\xi_{i}\right)}^{\alpha }\right).$$

**Remark** **5.** *Consider the real-valued function $\ell_{\alpha }:\left[0,1\right]\to \left[0,\infty \right)$ defined by $\ell_{\alpha }\left(x\right)=\frac{1}{\alpha -1}\left(x-x^{\alpha }\right)$. By Definition 8, the elements of the partition satisfy ${⨁}_{i=1}^{k}\xi_{i}=1$, which implies $\sum_{i=1}^{k}\tau^{R}\left(\xi_{i}\right)=1$ due to the additivity of the Riečan state over orthogonal elements. Consequently, the formula provided in Definition 13 can be expressed in the following summation form:*

(72) $$T_{\tau^{R}}^{\alpha }\left(\Xi \right)=\sum_{i=1}^{k}\ell_{\alpha }\left(\tau^{R}\left(\xi_{i}\right)\right).$$

*If we set α=2, we recover the logical entropy $H_{\tau^{R}}^{l}\left(\Xi \right)$ established in Definition 12. Furthermore, the function $\ell_{\alpha }$ is non-negative for every α∈(0,1)∪(1,∞). Specifically, if α∈(0,1), then $x^{\alpha }\geq x$ for every x∈[0,1], yielding $\ell_{\alpha }\left(x\right)=\frac{1}{\alpha -1}\left(x-x^{\alpha }\right)\geq 0$. Conversely, for α>1, we have $x^{\alpha }\leq x$ for every x∈[0,1], which yields $\ell_{\alpha }\left(x\right)\geq 0$ for every x∈[0,1].*

To algorithmically implement the Tsallis entropy formulation presented in Definition 13, we construct the following computational procedure:

Algorithm 3 outlines the computational sequence required to evaluate the generalized Tsallis entropy of a partition within a product Sheffer stroke basic algebra. The algorithm accepts the algebraic structure B=(B;∣), a finite partition Ξ, a Riečan state operator $\tau^{R}$, and the entropic index α as inputs. The execution initializes an accumulator, denoted as ${\mathrm{Sum}}_{\alpha }$, to aggregate the α-powered state probabilities. The procedure iterates through each orthogonal element $\xi_{i}\in \Xi$, computing the exponential term $\tau^{R}{\left(\xi_{i}\right)}^{\alpha }$ and adding it to the accumulator.    
**Algorithm 3:** Calculation of Tsallis Entropy of a Partition**Input**: Product Sheffer stroke basic algebra B=(B;∣), partition $\Xi =(\xi_{1},\ldots ,\xi_{k})$, Riečan state operator $\tau^{R}:B\to \left[0,1\right]$, entropic index α∈(0,1)∪(1,∞)**Output**: Tsallis entropy of Ξ, $T_{\tau^{R}}^{\alpha }\left(\Xi \right)$
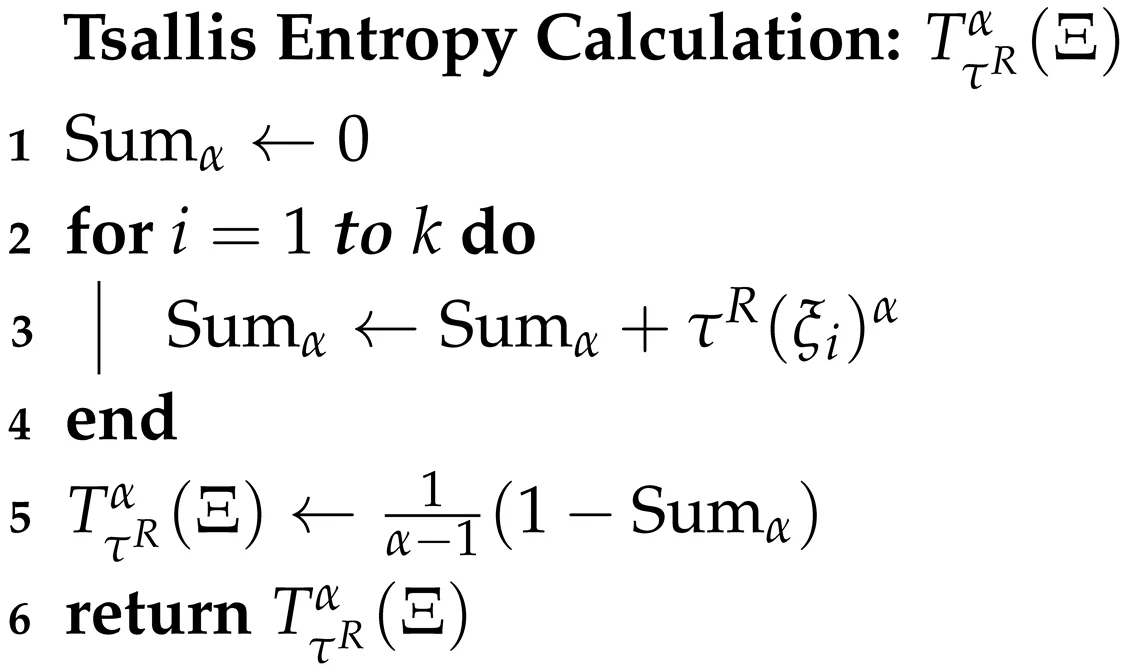


After the iterative loop, the algorithm applies the scaling factor $\frac{1}{\alpha -1}$ to the complement of the aggregated sum, mirroring the mathematical formulation established in Definition 13. Equivalently, this process reflects the summation of the non-negative $\ell_{\alpha }\left(\tau^{R}\left(\xi_{i}\right)\right)$ functions detailed in Remark 5. By parameterized adjustments of the index α, the algorithm remains stable and respects normalization boundaries, returning the exact scalar value of the Tsallis entropy.

**Corollary** **2.** *Under the assumptions of Proposition 2, the Shannon entropy, logical entropy, and Tsallis entropy of Ξ and Υ coincide. More precisely, for every Riečan state $\tau^{R}$ and every α∈(0,1)∪(1,∞),*

(73) $$H_{\tau^{R}}\left(\Xi \right)=H_{\tau^{R}}\left(\Upsilon \right),\;H_{\tau^{R}}^{l}\left(\Xi \right)=H_{\tau^{R}}^{l}\left(\Upsilon \right),\;T_{\tau^{R}}^{\alpha }\left(\Xi \right)=T_{\tau^{R}}^{\alpha }\left(\Upsilon \right).$$

**Proof.**  By Proposition 2, the partitions Ξ and Υ differ only by a permutation of their entries. The entropy functionals introduced in Definitions 11–13 depend only on the corresponding state values of the partition elements and are invariant under permutations. Therefore, the entropy values are equal.    □

**Theorem** **1.** *Let $\Xi =(\xi_{1},\ldots ,\xi_{k})$ be a partition in a product Sheffer stroke basic algebra B=(B;∣), and let $\tau^{R}:B\to \left[0,1\right]$ be a Riečan state. For any entropic index α∈(0,1)∪(1,∞), the Tsallis entropy of Ξ is bounded by*

(74) $$0\leq T_{\tau^{R}}^{\alpha }\left(\Xi \right)\leq \frac{1}{\alpha -1}\left(1-k^{1-\alpha }\right).$$

*Furthermore, the maximum entropy bound $T_{\tau^{R}}^{\alpha }\left(\Xi \right)=\frac{1}{\alpha -1}\left(1-k^{1-\alpha }\right)$ is achieved if and only if the Riečan state is uniformly distributed over the partition, meaning $\tau^{R}\left(\xi_{i}\right)=\frac{1}{k}$ for all i∈{1,…,k}.*

**Proof.**  The lower bound follows from the pointwise non-negativity of the Tsallis core function $\ell_{\alpha }\left(x\right)=\frac{1}{\alpha -1}\left(x-x^{\alpha }\right)$ on [0,1]. For the upper bound, the proof applies Jensen’s inequality to the strictly concave function $\ell_{\alpha }$ with equal weights, and the equality case follows from the strict concavity condition, which forces all partition state values to be equal.

The lower bound, $T_{\tau^{R}}^{\alpha }\left(\Xi \right)\geq 0$, follows from the non-negativity of the function $\ell_{\alpha }\left(x\right)=\frac{1}{\alpha -1}\left(x-x^{\alpha }\right)$ over the interval [0,1], as verified in Remark 5. The summation of these non-negative terms ensures $T_{\tau^{R}}^{\alpha }\left(\Xi \right)\geq 0$.

To establish the upper bound, consider the function

(75) $$\ell_{\alpha }\left(x\right)=\frac{1}{\alpha -1}\left(x-x^{\alpha }\right),\;x\in \left[0,1\right].$$

For every α∈(0,1)∪(1,∞), its second derivative is given by

(76) $$\ell_{\alpha }^{\prime \prime }\left(x\right)=-\alpha x^{\alpha -2}<0\;\mathrm{for}\;\mathrm{all}\;x\in \left(0,1\right].$$

Hence, $\ell_{\alpha }$ is strictly concave on (0,1], and by continuity, concave on [0,1].

By Definition 8, Ξ is a partition, implying ${⨁}_{i=1}^{k}\xi_{i}=1$, which maps to $\sum_{i=1}^{k}\tau^{R}\left(\xi_{i}\right)=1$ under the Riečan state.

Moreover, the partition probabilities satisfy $\tau^{R}\left(\xi_{i}\right)\in \left[0,1\right]$ for all i∈{1,…,k}, and the equal coefficients satisfy the normalization condition

(77) $$\sum_{i=1}^{k}\frac{1}{k}=1.$$

Thus, Jensen’s inequality applies to the concave function $\ell_{\alpha }$ with equal weights $\frac{1}{k}$.

Therefore,

(78) $$\ell_{\alpha }\left(\sum_{i=1}^{k}\frac{1}{k}\tau^{R}\left(\xi_{i}\right)\right)\geq \sum_{i=1}^{k}\frac{1}{k}\ell_{\alpha }\left(\tau^{R}\left(\xi_{i}\right)\right).$$

Substituting the normalization condition $\sum_{i=1}^{k}\tau^{R}\left(\xi_{i}\right)=1$ into the left-hand side and factoring out the constant $\frac{1}{k}$ on the right-hand side yields

(79) $$\ell_{\alpha }\left(\frac{1}{k}\right)\geq \frac{1}{k}\sum_{i=1}^{k}\ell_{\alpha }\left(\tau^{R}\left(\xi_{i}\right)\right).$$

Since the summation term on the right side corresponds to the Tsallis entropy $T_{\tau^{R}}^{\alpha }\left(\Xi \right)$ (Remark 5), multiplying both sides by *k* gives

(80) $$T_{\tau^{R}}^{\alpha }\left(\Xi \right)\leq k\;\ell_{\alpha }\left(\frac{1}{k}\right).$$

Expanding $\ell_{\alpha }\left(\frac{1}{k}\right)$ according to its definition, we obtain

(81) $$T_{\tau^{R}}^{\alpha }\left(\Xi \right)\leq k\left(\frac{1}{\alpha -1}\left(\frac{1}{k}-{\left(\frac{1}{k}\right)}^{\alpha }\right)\right)=\frac{1}{\alpha -1}\left(1-k^{1-\alpha }\right).$$

Finally, since $\ell_{\alpha }$ is strictly concave, the equality condition in Jensen’s inequality holds if and only if all input arguments are equal. Therefore, the upper bound is attained if and only if $\tau^{R}\left(\xi_{1}\right)=\tau^{R}\left(\xi_{2}\right)=\ldots =\tau^{R}\left(\xi_{k}\right)$. Because $\sum_{i=1}^{k}\tau^{R}\left(\xi_{i}\right)=1$, this is equivalent to $\tau^{R}\left(\xi_{i}\right)=\frac{1}{k}$ for every i∈{1,…,k}.    □

To demonstrate the theoretical boundaries formulated in Theorem 1, we construct a numerical example, providing the algebraic structure, the operational table, and the corresponding state evaluation.

**Figure 1:**
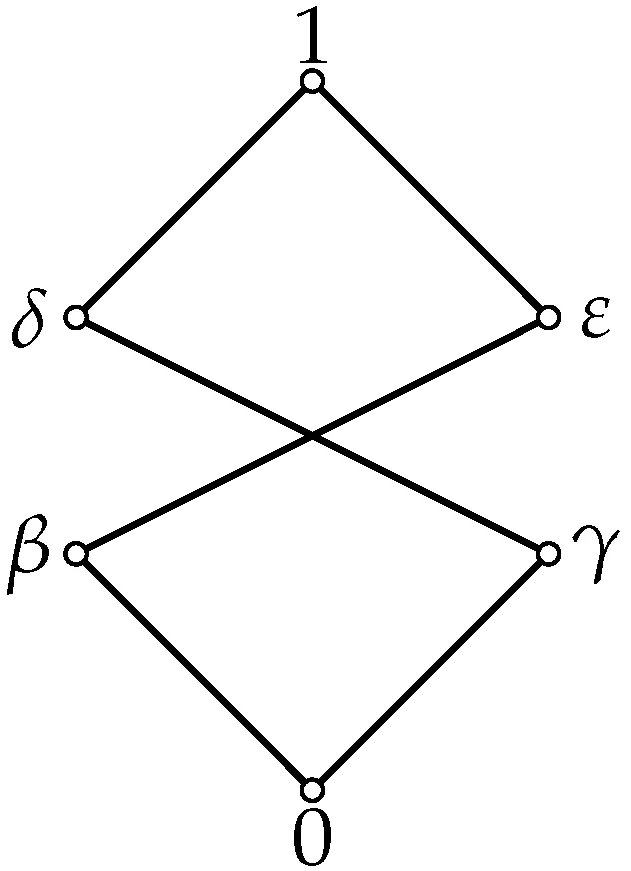
Hasse Diagram of *M*.

**Example** **1.** *Let M={0,β,γ,δ,ε,1} be a partially ordered set where 0<β<ε<1 and 0<γ<δ<1. The elements β and γ,δ are incomparable, and γ is incomparable with β,ε. The Hasse diagram of M and the Cayley table defining the Sheffer stroke operation* ∣ *on M are presented in Figure 1 and Table 2.*

*The structure M=(M;∣) forms a finite product Sheffer stroke basic algebra in the sense of Definition 7. Define a Riečan state operator $\tau^{R}:M\to \left[0,1\right]$ such that $\tau^{R}\left(0\right)=0$, $\tau^{R}\left(1\right)=1$, $\tau^{R}\left(\beta \right)=\tau^{R}\left(\varepsilon \right)=3/4$, and $\tau^{R}\left(\gamma \right)=\tau^{R}\left(\delta \right)=1/4$.*

*Consider the subset Ξ=(β,γ) of size k=2. To qualify as a partition, the Sheffer sum of its elements must equal 1. Using the operational matrix in Table 2, we verify the condition (β∣β)∣(γ∣γ)=1. The table yields*

β∣β=δandγ∣γ=ε.

*Substituting these into the condition yields*

δ∣ε=1.

*Since the condition is satisfied, Ξ=(β,γ) is a valid partition in M. The additivity of the state holds, as $\tau^{R}\left(\beta \right)+\tau^{R}\left(\gamma \right)=\frac{3}{4}+\frac{1}{4}=1$.*

*We compute the Tsallis entropy of this partition for the entropic index α=2:*

$$\begin{array}{cc} T_{\tau^{R}}^{2}\left(\Xi \right) & =\frac{1}{2-1}\left(1-\left[\tau^{R}{\left(\beta \right)}^{2}+\tau^{R}{\left(\gamma \right)}^{2}\right]\right)\\ \; & =1-\left[{\left(\frac{3}{4}\right)}^{2}+{\left(\frac{1}{4}\right)}^{2}\right]\\ \; & =1-\left(\frac{9}{16}+\frac{1}{16}\right)=1-\frac{10}{16}\\ \; & =\frac{6}{16}=0.375. \end{array}$$

*The theoretical upper bound for a partition of length k=2 and entropic index α=2 is*

$$T_{max}=\frac{1}{\alpha -1}\left(1-k^{1-\alpha }\right)=\frac{1}{2-1}\left(1-2^{1-2}\right)=1-\frac{1}{2}=0.5.$$

*Comparing the calculation with the theoretical bound, we observe that*

0≤0.375≤0.5.

*This inequality satisfies the boundaries established in Theorem 1. The computed entropy (0.375) is strictly below the theoretical maximum (0.5). As stated in the theorem, $T_{max}$ is achieved if and only if the Riečan state is uniformly distributed ($\tau^{R}\left(\xi_{i}\right)=\frac{1}{k}=0.5$). Since the partition elements hold disparate probabilities ($\tau^{R}\left(\beta \right)=0.75$ and $\tau^{R}\left(\gamma \right)=0.25$), the equality condition is not met.*

To analyze the hierarchical relationship between different partitions and their Sheffer stroke joint refinement constructions within the algebraic structure, we establish the following properties of the refinement relation.

**Lemma** **9.** *Let Ξ,Υ,Θ, and Φ be partitions in a product Sheffer stroke basic algebra B=(B;∣). For the associated admissible Sheffer stroke joint partitions, the refinement relation ≤ satisfies the following properties whenever the required recursive sum and orthogonal distributivity conditions used in the corresponding joint constructions are satisfied:*

(*i*) *Ξ≤(Ξ∣Υ)∣(Ξ∣Υ);*
(*ii*) *If Ξ≤Υ, then (Ξ∣Θ)∣(Ξ∣Θ)≤(Υ∣Θ)∣(Υ∣Θ);*
(*iii*) *If Ξ≤Υ and Θ≤Φ, then (Ξ∣Θ)∣(Ξ∣Θ)≤(Υ∣Φ)∣(Υ∣Φ);*
(*iv*) *If Ξ≤Υ and Υ≤Θ, then Ξ≤Θ.*

**Proof.**  Let the elements of the given partitions be denoted as $\Xi =(\xi_{1},\ldots ,\xi_{k})$, $\Upsilon =(\upsilon_{1},\ldots ,\upsilon_{l})$, $\Theta =(\theta_{1},\ldots ,\theta_{m})$, and $\Phi =(\phi_{1},\ldots ,\phi_{n})$.

(*i*) The elements of the raw joint family are $\zeta_{ij}=\left(\xi_{i}|\upsilon_{j}\right)|\left(\xi_{i}|\upsilon_{j}\right)$. For any fixed element $\xi_{i}\in \Xi$, applying the distributivity of the derived product over the Sheffer sum ⨁ yields

(82) $$\overset{l}{\underset{j=1}{⨁}}\zeta_{ij}=\left(\xi_{i}|\overset{l}{\underset{j=1}{⨁}}\upsilon_{j}\right)|\left(\xi_{i}|\overset{l}{\underset{j=1}{⨁}}\upsilon_{j}\right).$$

Since Υ is a valid partition, ${⨁}_{j=1}^{l}\upsilon_{j}=1$. Utilizing the identity property $\left(\xi_{i}|1\right)|\left(\xi_{i}|1\right)=\xi_{i}$, we obtain $\xi_{i}={⨁}_{j=1}^{l}\zeta_{ij}$. In the associated joint partition, zero elements are removed. By Lemma 7, omitting the zero components preserves the corresponding recursive Sheffer sum. Let $J_{i}=\left\{j|\zeta_{ij}\neq 0\right\}$ be the index subset for the nonzero elements. Then $\xi_{i}={⨁}_{j\in J_{i}}\zeta_{ij}$. Since nonzero joint elements are pairwise distinct, the sets $J_{i}$ disjointly partition the index space of the joint partition. Therefore, Ξ≤(Ξ∣Υ)∣(Ξ∣Υ).

(*ii*) Assume Ξ≤Υ. By Definition 9, there exists an index partition $\left(J_{1},\ldots ,J_{k}\right)$ such that $\xi_{i}={⨁}_{j\in J_{i}}\upsilon_{j}$. Substituting this into the joint elements formed with Θ gives

(83) $$\left(\xi_{i}|\theta_{r}\right)|\left(\xi_{i}|\theta_{r}\right)=\left(\left(\underset{j\in J_{i}}{⨁}\upsilon_{j}\right)|\theta_{r}\right)|\left(\left(\underset{j\in J_{i}}{⨁}\upsilon_{j}\right)|\theta_{r}\right).$$

By commutativity of the Sheffer stroke, the expression may be rewritten so that the fixed argument appears in the first coordinate of the derived joint term. Applying the orthogonal distributivity condition in Definition 7(iv) to the resulting expression, with all sums evaluated under the fixed recursive convention, gives the required distributive form:

(84) $$\left(\xi_{i}|\theta_{r}\right)|\left(\xi_{i}|\theta_{r}\right)=\underset{j\in J_{i}}{⨁}\left(\left(\upsilon_{j}|\theta_{r}\right)|\left(\upsilon_{j}|\theta_{r}\right)\right).$$

Filtering out any zero elements from both sides preserves the equality. The nonzero elements on the right side belong strictly to the joint partition (Υ∣Θ)∣(Υ∣Θ). The restricted index mapping maintains the disjoint partitioning condition. Thus, (Ξ∣Θ)∣(Ξ∣Θ)≤(Υ∣Θ)∣(Υ∣Θ).

(*iii*) Assume Ξ≤Υ and Θ≤Φ. There exist index partitions $J_{i}$ and $K_{r}$ such that $\xi_{i}={⨁}_{j\in J_{i}}\upsilon_{j}$ and $\theta_{r}={⨁}_{s\in K_{r}}\phi_{s}$. Analyzing an arbitrary joint element:

(85) $$\left(\xi_{i}|\theta_{r}\right)|\left(\xi_{i}|\theta_{r}\right)=\left((\underset{j\in J_{i}}{⨁}\upsilon_{j})|(\underset{s\in K_{r}}{⨁}\phi_{s})\right)|\left((\underset{j\in J_{i}}{⨁}\upsilon_{j})|(\underset{s\in K_{r}}{⨁}\phi_{s})\right).$$

Applying the orthogonal distributivity condition in Definition 7(iv) successively and using the commutativity of the Sheffer stroke to obtain the required right-hand form gives

(86) $$\left(\xi_{i}|\theta_{r}\right)|\left(\xi_{i}|\theta_{r}\right)=\underset{j\in J_{i}}{⨁}\underset{s\in K_{r}}{⨁}\left(\left(\upsilon_{j}|\phi_{s}\right)|\left(\upsilon_{j}|\phi_{s}\right)\right).$$

Defining the composite block $L_{i,r}=\left\{\left(j,s\right)|j\in J_{i},s\in K_{r}\;\mathrm{and}\;\left(\upsilon_{j}|\phi_{s}\right)|\left(\upsilon_{j}|\phi_{s}\right)\neq 0\right\}$, and omitting zero terms, the equation translates directly to the refinement requirement. Hence, (Ξ∣Θ)∣(Ξ∣Θ)≤(Υ∣Φ)∣(Υ∣Φ).

(*iv*) Assume that Ξ≤Υ and Υ≤Θ. There exist index partitions $J_{i}$ and $M_{j}$ such that $\xi_{i}={⨁}_{j\in J_{i}}\upsilon_{j}$ and $\upsilon_{j}={⨁}_{t\in M_{j}}\theta_{t}$. Substitution yields

(87) $$\xi_{i}=\underset{j\in J_{i}}{⨁}\left(\underset{t\in M_{j}}{⨁}\theta_{t}\right).$$

Because the relevant subfamilies consist of mutually orthogonal partition components, the nested recursive sums represent the same orthogonal supremum determined by the union of the corresponding sub-indices. Thus, under the admissibility convention for finite orthogonal sums used throughout the paper, the nested refinement blocks may be replaced by the single recursively ordered block over the union of the indices. Let $\Gamma_{i}={⋃}_{j\in J_{i}}M_{j}$. Since the sets $J_{i}$ and $M_{j}$ are disjoint partition blocks, $\Gamma_{i}$ constitutes a valid index partition of Θ. Thus, $\xi_{i}={⨁}_{t\in \Gamma_{i}}\theta_{t}$, establishing Ξ≤Θ.    □

To illustrate the algebraic mechanics of the refinement relation established in Lemma 9, we apply property (i) to the structural framework and operational matrix defined in Example 1.

**Example** **2.** *Recall the product Sheffer stroke basic algebra M=(M;∣) from Example 1. The operation* ∣ *is given in Table 2.*

*Let Ξ=(β,γ) and Υ=(δ,ε) be two subsets of M. We verify that both subsets constitute partitions. First, checking the pairwise orthogonality, Table 2 shows that β∣γ=1, and checking completeness, we obtain*

(β∣β)∣(γ∣γ)=δ∣ε=1,

*confirming that Ξ is a valid partition. Similarly, for Υ, the elements are orthogonal (δ∣ε=1) and satisfy completeness*

(δ∣δ)∣(ε∣ε)=β∣γ=1,

*so Υ is also a partition in M.*

*All finite Sheffer sums appearing in this example are evaluated according to the fixed left-recursive convention of Definition 5. In particular, no associativity, order independence, or unrestricted lattice-join interpretation is used in the verification below.*

*We now illustrate property (i) of Lemma 9 for the fixed ordered pair (Ξ,Υ). First, we compute the raw joint family*

$$J\left(\Xi ,\Upsilon \right)=\left(\zeta_{11},\zeta_{12},\zeta_{21},\zeta_{22}\right),$$

*where*

$$\zeta_{ij}=\left(\xi_{i}|\upsilon_{j}\right)|\left(\xi_{i}|\upsilon_{j}\right).$$

*Using Table 2, we obtain*

$$\begin{array}{cc} \zeta_{11} & =(\beta |\delta )|(\beta |\delta )=1|1=0,\\ \zeta_{12} & =(\beta |\varepsilon )|(\beta |\varepsilon )=\delta |\delta =\beta ,\\ \zeta_{21} & =(\gamma |\delta )|(\gamma |\delta )=\varepsilon |\varepsilon =\gamma ,\\ \zeta_{22} & =(\gamma |\varepsilon )|(\gamma |\varepsilon )=1|1=0. \end{array}$$

*Thus,*

J(Ξ,Υ)=(0,β,γ,0).

*After deleting the zero entries, the associated admissible joint partition is*

$${\left(\Xi |\Upsilon \right)}_{\mathrm{adm}}=\left(\beta ,\gamma \right),$$

*which coincides with Ξ.*

*Here ${\left(\Xi |\Upsilon \right)}_{\mathrm{adm}}$ denotes only the nonzero part of the associated admissible Sheffer stroke joint family obtained from the displayed fixed componentwise construction. It is not an unrestricted lattice join of Ξ and Υ.*

*To verify the refinement relation*

$$\Xi \leq {\left(\Xi |\Upsilon \right)}_{\mathrm{adm}},$$

*we take the index blocks*

$$J_{1}=\left\{1\right\},\;J_{2}=\left\{2\right\}.$$

*Then*

$$\xi_{1}=\beta =\underset{j\in J_{1}}{⨁}{\left(\Xi |\Upsilon \right)}_{\mathrm{adm},j},\;\xi_{2}=\gamma =\underset{j\in J_{2}}{⨁}{\left(\Xi |\Upsilon \right)}_{\mathrm{adm},j}.$$

*Hence, $\Xi \leq {\left(\Xi |\Upsilon \right)}_{\mathrm{adm}}$, illustrating property (i) of Lemma 9.*

*This verification is an oriented admissible-refinement calculation for the ordered construction J(Ξ,Υ). It does not assert that an arbitrary raw joint family, or a construction obtained by interchanging the ordered pair without separately checking the corresponding admissibility and marginalization conditions, automatically yields the same refinement relation.*

**Proposition** **6.** *Let $\Xi =(\xi_{1},\ldots ,\xi_{k})$ be a partition in a product Sheffer stroke basic algebra B=(B;∣), and let $\tau^{R}:B\to \left[0,1\right]$ be a Riečan state. For every υ∈B, the following properties are satisfied:*

(*i*) *$\sum_{i=1}^{k}\tau^{R}\left(\left(\xi_{i}|\upsilon \right)|\left(\xi_{i}|\upsilon \right)\right)=\tau^{R}\left(\upsilon \right)$;*
(*ii*) *$\sum_{i=1}^{k}\tau^{R}\left(\xi_{i}/\upsilon \right)=1$, provided that $\tau^{R}\left(\upsilon \right)>0$.*

**Proof.**  (i) By the definition of a partition, the elements $\xi_{i}$ are mutually orthogonal, and their recursive Sheffer sum yields the greatest element: ${⨁}_{i=1}^{k}\xi_{i}=1$. For any element υ∈B, the identity property yields

(1∣υ)∣(1∣υ)=υ.

Substituting the partition condition $1={⨁}_{i=1}^{k}\xi_{i}$ into this identity, we obtain

(88) $$\left((\overset{k}{\underset{i=1}{⨁}}\xi_{i})|\upsilon \right)|\left((\overset{k}{\underset{i=1}{⨁}}\xi_{i})|\upsilon \right)=\upsilon .$$

Using commutativity of the Sheffer stroke together with the orthogonal distributivity condition in Definition 7(iv), the derived product distributes over the recursive sum ⨁ in the required form. This evaluates to the generalized Sheffer sum of the joint elements:

(89) $$\overset{k}{\underset{i=1}{⨁}}\left(\left(\xi_{i}|\upsilon \right)|\left(\xi_{i}|\upsilon \right)\right)=\upsilon .$$

Applying the Riečan state operator $\tau^{R}$ to both sides and utilizing the additivity property of the Riečan state over orthogonal components (Definition 4), we translate the algebraic operation into a summation:

(90) $$\sum_{i=1}^{k}\tau^{R}\left(\left(\xi_{i}|\upsilon \right)|\left(\xi_{i}|\upsilon \right)\right)=\tau^{R}\left(\upsilon \right).$$

This proves (i).

(*ii*) Let $\tau^{R}\left(\upsilon \right)>0$. By the definition of the conditional state, we substitute $\tau^{R}\left(\xi_{i}/\upsilon \right)=\frac{\tau^{R}\left(\left(\xi_{i}|\upsilon \right)|\left(\xi_{i}|\upsilon \right)\right)}{\tau^{R}\left(\upsilon \right)}$ into the arithmetic sum. Summing over all indices i∈{1,…,k} yields

(91) $$\sum_{i=1}^{k}\tau^{R}\left(\xi_{i}/\upsilon \right)=\sum_{i=1}^{k}\frac{\tau^{R}\left(\left(\xi_{i}|\upsilon \right)|\left(\xi_{i}|\upsilon \right)\right)}{\tau^{R}\left(\upsilon \right)}.$$

Since the denominator $\tau^{R}\left(\upsilon \right)$ acts as a constant with respect to the summation index *i*, factoring it out of the sum yields

(92) $$\sum_{i=1}^{k}\tau^{R}\left(\xi_{i}/\upsilon \right)=\frac{1}{\tau^{R}\left(\upsilon \right)}\sum_{i=1}^{k}\tau^{R}\left(\left(\xi_{i}|\upsilon \right)|\left(\xi_{i}|\upsilon \right)\right).$$

Substituting the equality established in part (i) into the remaining summation provides:

(93) $$\sum_{i=1}^{k}\tau^{R}\left(\xi_{i}/\upsilon \right)=\frac{1}{\tau^{R}\left(\upsilon \right)}\tau^{R}\left(\upsilon \right)=1.$$

This proves the normalization property of the conditional state.    □

**Lemma** **10.** *Let $\Xi =(\xi_{1},\ldots ,\xi_{k})$ be a partition in a Sheffer stroke basic algebra B=(B;∣), and let $\tau^{R}:B\to \left[0,1\right]$ be a faithful Riečan state. For any element υ∈B, the equality*

(94) $$\sum_{i=1}^{k}\tau^{R}\left((\left(\left(\upsilon |\upsilon \right)|\xi_{i}\right)|\xi_{i})|\left(\left(\left(\upsilon |\upsilon \right)|\xi_{i}\right)|\xi_{i}\right)\right)=\tau^{R}\left(\upsilon \right)$$

*holds if and only if B satisfies the distributive lattice property for υ over the partition Ξ, defined as*

(95) $$\overset{k}{\underset{i=1}{⨁}}\left((\left(\left(\upsilon |\upsilon \right)|\xi_{i}\right)|\xi_{i})|\left(\left(\left(\upsilon |\upsilon \right)|\xi_{i}\right)|\xi_{i}\right)\right)=\upsilon .$$

**Proof.**  (⇐) Assume that B satisfies the distributive property for υ over Ξ. By Definition 8, ${⨁}_{i=1}^{k}\xi_{i}=1$. Consequently, the infimum evaluates to 1∧υ=υ. Applying the distributive hypothesis, the Sheffer sum of the individual infimums, represented as $\left(\left(\left(\upsilon |\upsilon \right)|\xi_{i}\right)|\xi_{i}\right)|\left(\left(\left(\upsilon |\upsilon \right)|\xi_{i}\right)|\xi_{i}\right)$, equals υ. Since $\xi_{i}\wedge \upsilon \leq \xi_{i}$, the resulting elements preserve the mutual orthogonality of the partition Ξ. Applying the additivity of the Riečan state $\tau^{R}$ over these orthogonal elements yields the arithmetic sum:

(96) $$\sum_{i=1}^{k}\tau^{R}\left((\left(\left(\upsilon |\upsilon \right)|\xi_{i}\right)|\xi_{i})|\left(\left(\left(\upsilon |\upsilon \right)|\xi_{i}\right)|\xi_{i}\right)\right)=\tau^{R}\left(\upsilon \right).$$

(⇒) Conversely, assume the equality holds for the faithful Riečan state $\tau^{R}$. Let $\zeta_{i}=\left(\left(\left(\upsilon |\upsilon \right)|\xi_{i}\right)|\xi_{i}\right)|\left(\left(\left(\upsilon |\upsilon \right)|\xi_{i}\right)|\xi_{i}\right)$, which defines the infimum $\xi_{i}\wedge \upsilon$. Since $\zeta_{i}\leq \xi_{i}$ for all *i*, the elements $\zeta_{i}$ are mutually orthogonal, and their Sheffer sum ${⨁}_{i=1}^{k}\zeta_{i}$ forms a well-defined element in *B*, denoted as ω. By the properties of the infimum over a partition, ω≤υ. Furthermore, due to the additivity of the state over orthogonal sums, $\tau^{R}\left(\omega \right)=\sum_{i=1}^{k}\tau^{R}\left(\zeta_{i}\right)$. Substituting the hypothesis into this equation yields $\tau^{R}\left(\omega \right)=\tau^{R}\left(\upsilon \right)$. Because $\tau^{R}$ is a faithful state and ω≤υ, the equality $\tau^{R}\left(\omega \right)=\tau^{R}\left(\upsilon \right)$ implies ω=υ. Therefore, ${⨁}_{i=1}^{k}\zeta_{i}=\upsilon$, which constitutes the distributive property over the given partition.    □

**Theorem** **2.** *Let $\Xi =(\xi_{1},\ldots ,\xi_{k})$ and $\Upsilon =(\upsilon_{1},\ldots ,\upsilon_{l})$ be partitions in a product Sheffer stroke basic algebra B=(B;∣) equipped with a Riečan state $\tau^{R}:B\to \left[0,1\right]$. If Ξ≤Υ, then*

(97) $$T_{\tau^{R}}^{\alpha }\left(\Xi \right)\leq T_{\tau^{R}}^{\alpha }\left(\Upsilon \right)$$

*for every entropic index α∈(0,1)∪(1,∞).*

**Proof.**  By Definition 9, the condition Ξ≤Υ implies the existence of a family $\left(J_{1},\ldots ,J_{k}\right)$ of non-empty, pairwise disjoint index subsets of {1,…,l} satisfying

(98) $$\overset{k}{\underset{i=1}{⋃}}J_{i}=\left\{1,\ldots ,l\right\}$$

such that each component $\xi_{i}\in \Xi$ can be written as

(99) $$\xi_{i}=\underset{j\in J_{i}}{⨁}\upsilon_{j}\;\mathrm{for}\;\mathrm{each}\;i\in \left\{1,\ldots ,k\right\}.$$

Since $\Upsilon =(\upsilon_{1},\ldots ,\upsilon_{l})$ is a partition in the sense of Definition 8, its elements are pairwise orthogonal; that is, $\upsilon_{j}|\upsilon_{j^{\prime }}=1$ for all $j\neq j^{\prime }$. Consequently, for each fixed index i∈{1,…,k}, the sub-family ${\left\{\upsilon_{j}\right\}}_{j\in J_{i}}$ is again pairwise orthogonal. By repeated application of the additivity axiom of the Riečan state $\tau^{R}$ to orthogonal elements, we obtain

(100) $$\tau^{R}\left(\xi_{i}\right)=\tau^{R}\left(\underset{j\in J_{i}}{⨁}\upsilon_{j}\right)=\sum_{j\in J_{i}}\tau^{R}\left(\upsilon_{j}\right)\;\mathrm{for}\;\mathrm{each}\;i\in \left\{1,\ldots ,k\right\}.$$

Because the index sets $J_{1},\ldots ,J_{k}$ partition {1,…,l}, every state weight $\tau^{R}\left(\upsilon_{j}\right)$ is counted exactly once in (100). We now distinguish two cases according to the value of α.

**Case** **1:** α>1.

For α>1, the function $f\left(x\right)=x^{\alpha }$ on [0,∞) satisfies

(101) $${\left(\sum_{r=1}^{m}a_{r}\right)}^{\alpha }\geq \sum_{r=1}^{m}a_{r}^{\alpha }\;\mathrm{for}\;\mathrm{all}\;a_{r}\geq 0.$$

Applying this to the non-negative numbers $\tau^{R}\left(\upsilon_{j}\right)$, $j\in J_{i}$, we obtain

(102) $${\left(\tau^{R}\left(\xi_{i}\right)\right)}^{\alpha }={\left(\sum_{j\in J_{i}}\tau^{R}\left(\upsilon_{j}\right)\right)}^{\alpha }\geq \sum_{j\in J_{i}}{\left(\tau^{R}\left(\upsilon_{j}\right)\right)}^{\alpha }\;\mathrm{for}\;\mathrm{each}\;i\in \left\{1,\ldots ,k\right\}.$$

Summing over all i∈{1,…,k} and using the fact that ${\left\{J_{i}\right\}}_{i=1}^{k}$ is a disjoint cover of {1,…,l}, we get

(103) $$\sum_{i=1}^{k}{\left(\tau^{R}\left(\xi_{i}\right)\right)}^{\alpha }\geq \sum_{i=1}^{k}\sum_{j\in J_{i}}{\left(\tau^{R}\left(\upsilon_{j}\right)\right)}^{\alpha }=\sum_{j=1}^{l}{\left(\tau^{R}\left(\upsilon_{j}\right)\right)}^{\alpha }.$$

Multiplying by −1 reverses the inequality, and adding 1 preserves it. Thus,

(104) $$1-\sum_{i=1}^{k}{\left(\tau^{R}\left(\xi_{i}\right)\right)}^{\alpha }\leq 1-\sum_{j=1}^{l}{\left(\tau^{R}\left(\upsilon_{j}\right)\right)}^{\alpha }.$$

Since $\frac{1}{\alpha -1}>0$, multiplying by this factor preserves the inequality and yields

(105) $$T_{\tau^{R}}^{\alpha }\left(\Xi \right)=\frac{1}{\alpha -1}\left(1-\sum_{i=1}^{k}{\left(\tau^{R}\left(\xi_{i}\right)\right)}^{\alpha }\right)\leq \frac{1}{\alpha -1}\left(1-\sum_{j=1}^{l}{\left(\tau^{R}\left(\upsilon_{j}\right)\right)}^{\alpha }\right)=T_{\tau^{R}}^{\alpha }\left(\Upsilon \right).$$

**Case** **2:** α∈(0,1).

For 0<α<1, the function $f\left(x\right)=x^{\alpha }$ on [0,∞) satisfies

(106) $${\left(\sum_{r=1}^{m}a_{r}\right)}^{\alpha }\leq \sum_{r=1}^{m}a_{r}^{\alpha }\;\mathrm{for}\;\mathrm{all}\;a_{r}\geq 0.$$

Applying this to (100), for each i∈{1,…,k} we obtain

(107) $${\left(\tau^{R}\left(\xi_{i}\right)\right)}^{\alpha }={\left(\sum_{j\in J_{i}}\tau^{R}\left(\upsilon_{j}\right)\right)}^{\alpha }\leq \sum_{j\in J_{i}}{\left(\tau^{R}\left(\upsilon_{j}\right)\right)}^{\alpha }.$$

Summing over all i∈{1,…,k} gives

(108) $$\sum_{i=1}^{k}{\left(\tau^{R}\left(\xi_{i}\right)\right)}^{\alpha }\leq \sum_{j=1}^{l}{\left(\tau^{R}\left(\upsilon_{j}\right)\right)}^{\alpha }.$$

Multiplying by −1 reverses the inequality, and adding 1 preserves it. Hence,

(109) $$1-\sum_{i=1}^{k}{\left(\tau^{R}\left(\xi_{i}\right)\right)}^{\alpha }\geq 1-\sum_{j=1}^{l}{\left(\tau^{R}\left(\upsilon_{j}\right)\right)}^{\alpha }.$$

Since $\frac{1}{\alpha -1}<0$, multiplying by this factor reverses the inequality and yields

(110) $$T_{\tau^{R}}^{\alpha }\left(\Xi \right)=\frac{1}{\alpha -1}\left(1-\sum_{i=1}^{k}{\left(\tau^{R}\left(\xi_{i}\right)\right)}^{\alpha }\right)\leq \frac{1}{\alpha -1}\left(1-\sum_{j=1}^{l}{\left(\tau^{R}\left(\upsilon_{j}\right)\right)}^{\alpha }\right)=T_{\tau^{R}}^{\alpha }\left(\Upsilon \right).$$

Therefore, in both cases,

(111) $$T_{\tau^{R}}^{\alpha }\left(\Xi \right)\leq T_{\tau^{R}}^{\alpha }\left(\Upsilon \right).$$

This completes the proof.    □

**Proposition** **7.** *Let $\Xi =(\xi_{1},\ldots ,\xi_{k})$ and $\Upsilon =(\upsilon_{1},\ldots ,\upsilon_{l})$ be partitions in a product Sheffer stroke basic algebra B=(B;∣), and let $\tau^{R}:B\to \left[0,1\right]$ be a Riečan state. For any entropic index α>1, the following inequality holds:*

(112) $$\sum_{j=1}^{l}{\left(\tau^{R}\left(\upsilon_{j}\right)\right)}^{\alpha }\sum_{i=1}^{k}\ell_{\alpha }\left(\tau^{R}\left(\xi_{i}/\upsilon_{j}\right)\right)\leq T_{\tau^{R}}^{\alpha }\left(\Xi \right),$$

*where $\ell_{\alpha }\left(x\right)=\frac{1}{\alpha -1}\left(x-x^{\alpha }\right)$ is the core entropic function.*

**Proof.**  For α>1, consider the core entropic function

(113) $$\ell_{\alpha }\left(x\right)=\frac{1}{\alpha -1}\left(x-x^{\alpha }\right),\;x\in \left[0,1\right].$$

Its second derivative is given by

(114) $$\ell_{\alpha }^{\prime \prime }\left(x\right)=-\alpha x^{\alpha -2}<0\;\mathrm{for}\;\mathrm{all}\;x\in \left(0,1\right].$$

Hence, $\ell_{\alpha }$ is strictly concave on (0,1], and by continuity, concave on [0,1].

Because Υ is a partition, the state values satisfy

(115) $$\tau^{R}\left(\upsilon_{j}\right)\geq 0\;\mathrm{and}\;\sum_{j=1}^{l}\tau^{R}\left(\upsilon_{j}\right)=1.$$

Thus, the numbers $\tau^{R}\left(\upsilon_{j}\right)$ form a normalized system of Jensen weights.

Applying Jensen’s inequality to the concave function $\ell_{\alpha }$ with weights $\tau^{R}\left(\upsilon_{j}\right)$, for each fixed i∈{1,…,k}, we obtain

(116) $$\sum_{j=1}^{l}\tau^{R}\left(\upsilon_{j}\right)\;\ell_{\alpha }\left(\tau^{R}\left(\xi_{i}/\upsilon_{j}\right)\right)\leq \ell_{\alpha }\left(\sum_{j=1}^{l}\tau^{R}\left(\upsilon_{j}\right)\tau^{R}\left(\xi_{i}/\upsilon_{j}\right)\right).$$

By the definition of the conditional state in the Sheffer stroke basic algebra,

(117) $$\tau^{R}\left(\upsilon_{j}\right)\tau^{R}\left(\xi_{i}/\upsilon_{j}\right)=\tau^{R}\left(\left(\upsilon_{j}|\xi_{i}\right)|\left(\upsilon_{j}|\xi_{i}\right)\right).$$

Substituting this identity into the argument of $\ell_{\alpha }$ yields

(118) $$\sum_{j=1}^{l}\tau^{R}\left(\upsilon_{j}\right)\;\ell_{\alpha }\left(\tau^{R}\left(\xi_{i}/\upsilon_{j}\right)\right)\leq \ell_{\alpha }\left(\sum_{j=1}^{l}\tau^{R}\left(\left(\upsilon_{j}|\xi_{i}\right)|\left(\upsilon_{j}|\xi_{i}\right)\right)\right).$$

By Proposition 6, the sum of these joint state realizations over the partition Υ equals the marginal state value $\tau^{R}\left(\xi_{i}\right)$. Therefore,

(119) $$\sum_{j=1}^{l}\tau^{R}\left(\upsilon_{j}\right)\;\ell_{\alpha }\left(\tau^{R}\left(\xi_{i}/\upsilon_{j}\right)\right)\leq \ell_{\alpha }\left(\tau^{R}\left(\xi_{i}\right)\right).$$

Summing over all i∈{1,…,k} gives

(120) $$\sum_{i=1}^{k}\sum_{j=1}^{l}\tau^{R}\left(\upsilon_{j}\right)\;\ell_{\alpha }\left(\tau^{R}\left(\xi_{i}/\upsilon_{j}\right)\right)\leq \sum_{i=1}^{k}\ell_{\alpha }\left(\tau^{R}\left(\xi_{i}\right)\right).$$

Reordering the summation on the left-hand side and using the definition of Tsallis entropy on the right-hand side, we obtain

(121) $$\sum_{j=1}^{l}\tau^{R}\left(\upsilon_{j}\right)\sum_{i=1}^{k}\ell_{\alpha }\left(\tau^{R}\left(\xi_{i}/\upsilon_{j}\right)\right)\leq T_{\tau^{R}}^{\alpha }\left(\Xi \right).$$

Since α>1 and $\tau^{R}\left(\upsilon_{j}\right)\in \left[0,1\right]$ for all j∈{1,…,l}, we have

(122) $${\left(\tau^{R}\left(\upsilon_{j}\right)\right)}^{\alpha }\leq \tau^{R}\left(\upsilon_{j}\right).$$

Moreover, $\ell_{\alpha }\left(x\right)\geq 0$ on [0,1]. Therefore,

(123) $${\left(\tau^{R}\left(\upsilon_{j}\right)\right)}^{\alpha }\sum_{i=1}^{k}\ell_{\alpha }\left(\tau^{R}\left(\xi_{i}/\upsilon_{j}\right)\right)\leq \tau^{R}\left(\upsilon_{j}\right)\sum_{i=1}^{k}\ell_{\alpha }\left(\tau^{R}\left(\xi_{i}/\upsilon_{j}\right)\right).$$

Summing over all j∈{1,…,l}, we obtain

(124) $$\sum_{j=1}^{l}{\left(\tau^{R}\left(\upsilon_{j}\right)\right)}^{\alpha }\sum_{i=1}^{k}\ell_{\alpha }\left(\tau^{R}\left(\xi_{i}/\upsilon_{j}\right)\right)\leq \sum_{j=1}^{l}\tau^{R}\left(\upsilon_{j}\right)\sum_{i=1}^{k}\ell_{\alpha }\left(\tau^{R}\left(\xi_{i}/\upsilon_{j}\right)\right).$$

Combining this with (121) yields

(125) $$\sum_{j=1}^{l}{\left(\tau^{R}\left(\upsilon_{j}\right)\right)}^{\alpha }\sum_{i=1}^{k}\ell_{\alpha }\left(\tau^{R}\left(\xi_{i}/\upsilon_{j}\right)\right)\leq T_{\tau^{R}}^{\alpha }\left(\Xi \right).$$

   □

**Theorem** **3.** *Let $\Xi =(\xi_{1},\ldots ,\xi_{k})$ and $\Upsilon =(\upsilon_{1},\ldots ,\upsilon_{l})$ be partitions in a product Sheffer stroke basic algebra B=(B;∣), and let $\tau^{R}:B\to \left[0,1\right]$ be a Riečan state. For any entropic index α>1, the joint Tsallis entropy satisfies the subadditivity property:*

(126) $$T_{\tau^{R}}^{\alpha }\left((\Xi |\Upsilon )|(\Xi |\Upsilon )\right)\leq T_{\tau^{R}}^{\alpha }\left(\Xi \right)+T_{\tau^{R}}^{\alpha }\left(\Upsilon \right).$$

**Proof.**  The proof expands the joint Tsallis entropy and rewrites each joint state value through the corresponding conditional state over Υ. After adding and subtracting the marginal α-power sum of Υ, the expression splits into the marginal Tsallis entropy of Υ plus a conditional contribution, which is bounded above by $T_{\tau^{R}}^{\alpha }\left(\Xi \right)$ via Proposition 7.

By the definition of joint Tsallis entropy in B=(B;∣), we have:

(127) $$T_{\tau^{R}}^{\alpha }\left((\Xi |\Upsilon )|(\Xi |\Upsilon )\right)=\frac{1}{\alpha -1}\left(1-\sum_{i=1}^{k}\sum_{j=1}^{l}{\left[\tau^{R}\left(\left(\xi_{i}|\upsilon_{j}\right)|\left(\xi_{i}|\upsilon_{j}\right)\right)\right]}^{\alpha }\right).$$

Applying the algebraic definition of the conditional state, we substitute the joint realization $\tau^{R}\left(\left(\xi_{i}|\upsilon_{j}\right)|\left(\xi_{i}|\upsilon_{j}\right)\right)=\tau^{R}\left(\upsilon_{j}\right)\tau^{R}\left(\xi_{i}/\upsilon_{j}\right)$ into the entropy formulation:

(128) $$T_{\tau^{R}}^{\alpha }\left((\Xi |\Upsilon )|(\Xi |\Upsilon )\right)=\frac{1}{\alpha -1}\left(1-\sum_{j=1}^{l}\tau^{R}{\left(\upsilon_{j}\right)}^{\alpha }\sum_{i=1}^{k}\tau^{R}{\left(\xi_{i}/\upsilon_{j}\right)}^{\alpha }\right).$$

We strategically add and subtract the marginal term $\sum_{j=1}^{l}\tau^{R}{\left(\upsilon_{j}\right)}^{\alpha }$ within the primary brackets:

(129) $$T_{\tau^{R}}^{\alpha }\left((\Xi |\Upsilon )|(\Xi |\Upsilon )\right)=\frac{1}{\alpha -1}\left(1-\sum_{j=1}^{l}\tau^{R}{\left(\upsilon_{j}\right)}^{\alpha }+\sum_{j=1}^{l}\tau^{R}{\left(\upsilon_{j}\right)}^{\alpha }-\sum_{j=1}^{l}\tau^{R}{\left(\upsilon_{j}\right)}^{\alpha }\sum_{i=1}^{k}\tau^{R}{\left(\xi_{i}/\upsilon_{j}\right)}^{\alpha }\right).$$

By Definition 13, the first two terms evaluate directly to the marginal Tsallis entropy $T_{\tau^{R}}^{\alpha }\left(\Upsilon \right)$. Factoring the common term $\tau^{R}{\left(\upsilon_{j}\right)}^{\alpha }$ from the remaining components yields

(130) $$T_{\tau^{R}}^{\alpha }\left((\Xi |\Upsilon )|(\Xi |\Upsilon )\right)=T_{\tau^{R}}^{\alpha }\left(\Upsilon \right)+\frac{1}{\alpha -1}\sum_{j=1}^{l}\tau^{R}{\left(\upsilon_{j}\right)}^{\alpha }\left(1-\sum_{i=1}^{k}\tau^{R}{\left(\xi_{i}/\upsilon_{j}\right)}^{\alpha }\right).$$

By Proposition 6, the conditional states over a partition sum to unity, $\sum_{i=1}^{k}\tau^{R}\left(\xi_{i}/\upsilon_{j}\right)=1$. Substituting this unity condition into the inner bracket allows us to express it via the core entropic function $\ell_{\alpha }\left(x\right)=\frac{1}{\alpha -1}\left(x-x^{\alpha }\right)$:

(131) $$\begin{array}{cc} T_{\tau^{R}}^{\alpha }\left((\Xi |\Upsilon )|(\Xi |\Upsilon )\right) & =T_{\tau^{R}}^{\alpha }\left(\Upsilon \right)+\sum_{j=1}^{l}\tau^{R}{\left(\upsilon_{j}\right)}^{\alpha }\left(\sum_{i=1}^{k}\frac{\tau^{R}\left(\xi_{i}/\upsilon_{j}\right)-\tau^{R}{\left(\xi_{i}/\upsilon_{j}\right)}^{\alpha }}{\alpha -1}\right)\\ \; & =T_{\tau^{R}}^{\alpha }\left(\Upsilon \right)+\sum_{j=1}^{l}\tau^{R}{\left(\upsilon_{j}\right)}^{\alpha }\sum_{i=1}^{k}\ell_{\alpha }\left(\tau^{R}\left(\xi_{i}/\upsilon_{j}\right)\right). \end{array}$$

This equation represents the exact algebraic chain rule for Tsallis entropy. By Proposition 7, the double summation term on the right-hand side is bounded above by $T_{\tau^{R}}^{\alpha }\left(\Xi \right)$. Substituting this theoretical upper bound into the equality yields the required inequality:

(132) $$T_{\tau^{R}}^{\alpha }\left((\Xi |\Upsilon )|(\Xi |\Upsilon )\right)\leq T_{\tau^{R}}^{\alpha }\left(\Upsilon \right)+T_{\tau^{R}}^{\alpha }\left(\Xi \right).$$

This completes the proof of subadditivity.    □

To demonstrate that the Tsallis entropy within a product Sheffer stroke basic algebra satisfies pseudo-additivity and concavity, we first formalize an explicit notion of statistical independence within this framework. In ordinary Boolean probability spaces, independence is expressed by the factorization of joint probabilities. In quantum-logical or non-classical algebraic settings, however, such a condition requires a more careful interpretation because the relevant algebraic contexts may be non-distributive, noncommuting in a concrete representation, or not jointly representable by a single classical event space. Therefore, in the present framework, statistical independence is introduced as a state-dependent factorization property on the Sheffer stroke joint refinement, rather than as an assumption of classical compatibility.

**Definition** **14.** *Let $\Xi =(\xi_{1},\ldots ,\xi_{k})$ and $\Upsilon =(\upsilon_{1},\ldots ,\upsilon_{l})$ be partitions in a product Sheffer stroke basic algebra B=(B;∣), and let $\tau^{R}:B\to \left[0,1\right]$ be a Riečan state. The partitions Ξ and Υ are said to be statistically independent with respect to $\tau^{R}$ if, for all i∈{1,…,k} and j∈{1,…,l}, the joint state evaluation factors multiplicatively:*

(133) $$\tau^{R}\left(\left(\xi_{i}|\upsilon_{j}\right)|\left(\xi_{i}|\upsilon_{j}\right)\right)=\tau^{R}\left(\xi_{i}\right)\tau^{R}\left(\upsilon_{j}\right).$$

*Equivalently, the state distribution induced on the joint partition (Ξ∣Υ)∣(Ξ∣Υ) factorizes pointwise into the product of the marginal state distributions of Ξ and Υ.*

**Remark** **6.** *It is important to clarify how Definition 14 relates to the underlying non-distributive structure of the basic algebra. In classical probability theory over Boolean algebras, statistical independence is formulated within a distributive event structure admitting a classical joint probability distribution for compatible events. By contrast, in a product Sheffer stroke basic algebra, the relevant logical structure is in general non-distributive, so the existence of a global Boolean event space or a classical Kolmogorovian joint model cannot be assumed a priori. Consequently, the notion of independence introduced above is strictly a state-dependent algebraic property. It does not assert that the partitions Ξ and Υ generate a distributive subalgebra, nor does it imply that the associated observables are jointly measurable in the classical sense. Rather, it serves as an internal factorization rule for the Riečan state $\tau^{R}$ on the joint partition elements, allowing the entropic formalism to encode factorized marginal behavior within a non-distributive logical background. The quantum-logical interpretation of this state-dependent factorization is clarified in Remark 7.*

**Remark** **7.** *In the quantum-logical interpretation, the statistical independence condition in Definition 14 should be understood as a condition on the probability weights assigned by the Riečan state to the algebraic joint refinement. More precisely, if the algebraic elements $\xi_{i}$ and $\upsilon_{j}$ are viewed as representatives of quantum propositions, then the factorization condition*

(134) $$\tau^{R}\left(\left(\xi_{i}|\upsilon_{j}\right)|\left(\xi_{i}|\upsilon_{j}\right)\right)=\tau^{R}\left(\xi_{i}\right)\tau^{R}\left(\upsilon_{j}\right)$$

*means that the state value assigned to the Sheffer stroke joint component is completely determined by the product of the two marginal state values.*

*In a classical Boolean event algebra, this is the standard definition of probabilistic independence. In a quantum-logical setting, however, it is a restrictive state-dependent property. It may hold when the two measurement contexts are compatible and generate a common Boolean substructure, provided that the chosen state assigns factorized probabilities on that substructure. It may also hold for special state assignments for which the algebraic joint evaluations reduce to products of the corresponding marginal values. Typical examples include compatible coarse-grained measurements or preparations whose outcome statistics are uncorrelated with respect to the joint refinement.*

*Conversely, this factorization condition is not expected to hold in general. It fails when the two contexts are statistically correlated under the chosen state. It may also fail for incompatible quantum propositions. In Hilbert-space realizations, noncommuting projections do not generally determine a classical joint probability model for simultaneous outcomes. In finite algebraic realizations, the obstruction may appear through constrained or null components of the Sheffer stroke joint refinement. In particular, one may have*

(135) $$\left(\xi_{i}|\upsilon_{j}\right)|\left(\xi_{i}|\upsilon_{j}\right)=0$$

*for some pair (i,j), even though both marginal state values $\tau^{R}\left(\xi_{i}\right)$ and $\tau^{R}\left(\upsilon_{j}\right)$ are nonzero. In such a case,*

(136) $$\tau^{R}\left(\left(\xi_{i}|\upsilon_{j}\right)|\left(\xi_{i}|\upsilon_{j}\right)\right)\neq \tau^{R}\left(\xi_{i}\right)\tau^{R}\left(\upsilon_{j}\right),$$

*and the factorization condition fails.*

*Thus, statistical independence in this framework should not be interpreted as a generic feature of quantum measurement contexts. It is a strict algebraic factorization condition imposed on the Riečan-state statistics of the Sheffer stroke joint refinement. Its failure indicates that the joint state values cannot be reconstructed from the marginal partitions alone. This deviation from factorization is precisely the algebraic quantity recorded by the pseudo-additive residual introduced later in Definition 18.*

**Theorem** **4.** *Let $\Xi =(\xi_{1},\ldots ,\xi_{k})$ and $\Upsilon =(\upsilon_{1},\ldots ,\upsilon_{l})$ be partitions in a product Sheffer stroke basic algebra B=(B;∣) that are statistically independent with respect to the Riečan state $\tau^{R}$ in the sense of Definition 14. Then, the Tsallis entropy satisfies the pseudo-additivity property:*

(137) $$T_{\tau^{R}}^{\alpha }\left((\Xi |\Upsilon )|(\Xi |\Upsilon )\right)=T_{\tau^{R}}^{\alpha }\left(\Xi \right)+T_{\tau^{R}}^{\alpha }\left(\Upsilon \right)+\left(1-\alpha \right)T_{\tau^{R}}^{\alpha }\left(\Xi \right)T_{\tau^{R}}^{\alpha }\left(\Upsilon \right).$$

**Proof.**  The proof uses the statistical independence condition to factor the double sum of joint α-powers into the product of the two marginal α-power sums. These marginal sums are then rewritten in terms of the corresponding Tsallis entropies, and a direct algebraic simplification yields the pseudo-additive identity.

By the definition of the Tsallis entropy, we have

(138) $$T_{\tau^{R}}^{\alpha }\left((\Xi |\Upsilon )|(\Xi |\Upsilon )\right)=\frac{1}{\alpha -1}\left(1-\sum_{i=1}^{k}\sum_{j=1}^{l}\tau^{R}{\left(\left(\xi_{i}|\upsilon_{j}\right)|\left(\xi_{i}|\upsilon_{j}\right)\right)}^{\alpha }\right).$$

Using the multiplicative independence condition from Definition 14, we obtain

(139) $$\begin{array}{cc} T_{\tau^{R}}^{\alpha }\left((\Xi |\Upsilon )|(\Xi |\Upsilon )\right)\; & =\frac{1}{\alpha -1}\left(1-\left(\sum_{i=1}^{k}\tau^{R}{\left(\xi_{i}\right)}^{\alpha }\right)\left(\sum_{j=1}^{l}\tau^{R}{\left(\upsilon_{j}\right)}^{\alpha }\right)\right)\\ \; & =\frac{1}{\alpha -1}\left(1-\left[1-\left(\alpha -1\right)T_{\tau^{R}}^{\alpha }\left(\Xi \right)\right]\left[1-\left(\alpha -1\right)T_{\tau^{R}}^{\alpha }\left(\Upsilon \right)\right]\right)\\ \; & =T_{\tau^{R}}^{\alpha }\left(\Xi \right)+T_{\tau^{R}}^{\alpha }\left(\Upsilon \right)-\left(\alpha -1\right)T_{\tau^{R}}^{\alpha }\left(\Xi \right)T_{\tau^{R}}^{\alpha }\left(\Upsilon \right). \end{array}$$

Since 1−α=−(α−1), this is equivalent to

(140) $$T_{\tau^{R}}^{\alpha }\left((\Xi |\Upsilon )|(\Xi |\Upsilon )\right)=T_{\tau^{R}}^{\alpha }\left(\Xi \right)+T_{\tau^{R}}^{\alpha }\left(\Upsilon \right)+\left(1-\alpha \right)T_{\tau^{R}}^{\alpha }\left(\Xi \right)T_{\tau^{R}}^{\alpha }\left(\Upsilon \right).$$

This completes the proof.    □

**Proposition** **8.** *Let $\tau_{1}^{R}$ and $\tau_{2}^{R}$ be Riečan states on a product Sheffer stroke basic algebra B=(B;∣). For any λ∈[0,1], the mapping $\tau_{\lambda }^{R}=\lambda \tau_{1}^{R}+\left(1-\lambda \right)\tau_{2}^{R}$ is a Riečan state on B.*

**Proof.**  This follows from the linearity of the state conditions $\left(\tau_{SH}^{R}1\right)$ and $\left(\tau_{SH}^{R}2\right)$.    □

**Remark** **8.** *It is useful to distinguish the entropy of a single admissible partition from the behavior of joint refinement state values associated with two partitions. A single partition of mutually orthogonal elements gives a normalized family of state values, so its marginal entropy has the same formal expression as the corresponding classical entropy of a finite probability vector. The specific contribution of the present framework appears when the Sheffer stroke joint components of two partitions, such as Ξ and Υ, are evaluated internally in the product Sheffer stroke algebra. In this setting, subadditivity bounds, conditional chain-type identities, and the pseudo-additive residual are formulated at the level of admissible Sheffer stroke joint refinements and Riečan-state values. These results should be understood as algebraic non-factorization statements within the present framework, rather than as operational contextuality claims.*

**Theorem** **5.** *Let $\Xi =(\xi_{1},\ldots ,\xi_{k})$ be a partition in a product Sheffer stroke basic algebra B=(B;∣). The Tsallis entropy $T_{\tau^{R}}^{\alpha }\left(\Xi \right)$ is a concave function with respect to the Riečan state operator. For any λ∈[0,1] and Riečan states $\tau_{1}^{R},\tau_{2}^{R}$ on B:*

(141) $$\lambda T_{\tau_{1}^{R}}^{\alpha }\left(\Xi \right)+\left(1-\lambda \right)T_{\tau_{2}^{R}}^{\alpha }\left(\Xi \right)\leq T_{\lambda \tau_{1}^{R}+\left(1-\lambda \right)\tau_{2}^{R}}^{\alpha }\left(\Xi \right).$$

**Proof.**  Let $\ell_{\alpha }\left(x\right)=\frac{1}{\alpha -1}\left(x-x^{\alpha }\right)$ and denote the convex combination of the states as $\tau_{\lambda }^{R}=\lambda \tau_{1}^{R}+\left(1-\lambda \right)\tau_{2}^{R}$. The Tsallis entropy is expressed as $T_{\tau^{R}}^{\alpha }\left(\Xi \right)=\sum_{i=1}^{k}\ell_{\alpha }\left(\tau^{R}\left(\xi_{i}\right)\right)$.

For every α∈(0,1)∪(1,∞), the second derivative evaluates to

(142) $$\ell_{\alpha }^{\prime \prime }\left(x\right)=-\alpha x^{\alpha -2}<0\;\mathrm{for}\;\mathrm{all}\;x\in \left(0,1\right].$$

Hence, the function $\ell_{\alpha }$ is strictly concave on (0,1], and by continuity, it is concave on [0,1].

By the definition of the convex combination of states, the evaluation on any partition element is $\tau_{\lambda }^{R}\left(\xi_{i}\right)=\lambda \tau_{1}^{R}\left(\xi_{i}\right)+\left(1-\lambda \right)\tau_{2}^{R}\left(\xi_{i}\right)$. Applying the definition of concavity for $\ell_{\alpha }$ pointwise to each individual element $\xi_{i}\in \Xi$, we obtain

(143) $$\ell_{\alpha }\left(\tau_{\lambda }^{R}\left(\xi_{i}\right)\right)=\ell_{\alpha }\left(\lambda \tau_{1}^{R}\left(\xi_{i}\right)+\left(1-\lambda \right)\tau_{2}^{R}\left(\xi_{i}\right)\right)\geq \lambda \ell_{\alpha }\left(\tau_{1}^{R}\left(\xi_{i}\right)\right)+\left(1-\lambda \right)\ell_{\alpha }\left(\tau_{2}^{R}\left(\xi_{i}\right)\right).$$

Summing this inequality over all partition indices i∈{1,…,k} yields

(144) $$\sum_{i=1}^{k}\ell_{\alpha }\left(\tau_{\lambda }^{R}\left(\xi_{i}\right)\right)\geq \lambda \sum_{i=1}^{k}\ell_{\alpha }\left(\tau_{1}^{R}\left(\xi_{i}\right)\right)+\left(1-\lambda \right)\sum_{i=1}^{k}\ell_{\alpha }\left(\tau_{2}^{R}\left(\xi_{i}\right)\right).$$

Substituting the definition of the Tsallis entropy into both sides of this summation establishes the required concavity:

(145) $$T_{\lambda \tau_{1}^{R}+\left(1-\lambda \right)\tau_{2}^{R}}^{\alpha }\left(\Xi \right)\geq \lambda T_{\tau_{1}^{R}}^{\alpha }\left(\Xi \right)+\left(1-\lambda \right)T_{\tau_{2}^{R}}^{\alpha }\left(\Xi \right).$$

   □

**Example** **3.** *To numerically validate the derived entropy functionals, we construct a finite product Sheffer stroke basic algebra M=(M;∣) of eight elements. While isomorphic to a classical Boolean algebra, this structure provides a transparent computational test-bed to verify subadditivity, state concavity, and the pseudo-additive residual under non-factorizing statistical conditions.*

**Figure 2:**
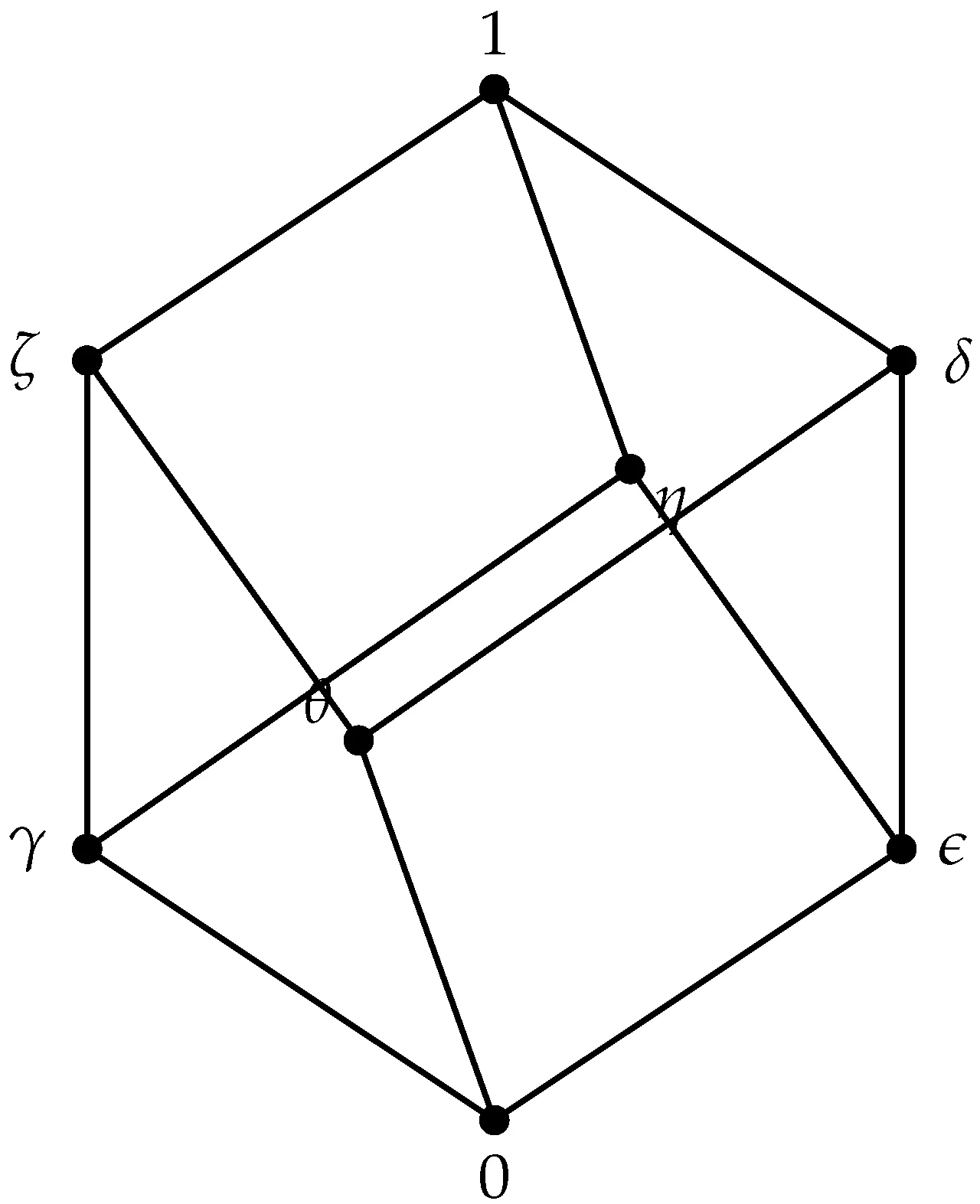
Hasse Diagram of the algebra M.

*Let M=(M;∣) be defined by the Hasse diagram in Figure 2 and the operational structure in Table 3. We denote the internal elements as*

M={0,γ,δ,ϵ,ζ,η,θ,1}.

*Solving the linear equations imposed by the Riečan orthogonal additivity axiom $\left(\tau_{SH}^{R}2\right)$ over M (for all x,y∈M such that x∣y=1), we construct two valid Riečan state operators $\tau_{1}^{R},\tau_{2}^{R}:M\to \left[0,1\right]$:*

*x*
0

γ



δ



ϵ



ζ



η



θ

1

$\tau_{1}^{R}\left(x\right)$

0

1/4



3/4



1/2



1/2



3/4



1/4

1

$\tau_{2}^{R}\left(x\right)$

0

1/6



5/6



1/2



1/2



2/3



1/3

1

*1*. *Verification of Subadditivity*

*Let the entropic index be α=2, and define the valid partitions Ξ=(γ,δ) and Υ=(ϵ,ζ). Under the state $\tau_{1}^{R}$, the marginal entropies evaluate to*

$$T_{\tau_{1}^{R}}^{2}\left(\Xi \right)=1-\left[\tau_{1}^{R}{\left(\gamma \right)}^{2}+\tau_{1}^{R}{\left(\delta \right)}^{2}\right]=1-\left(1/16+9/16\right)=0.375,$$

$$T_{\tau_{1}^{R}}^{2}\left(\Upsilon \right)=1-\left[\tau_{1}^{R}{\left(\epsilon \right)}^{2}+\tau_{1}^{R}{\left(\zeta \right)}^{2}\right]=1-\left(1/4+1/4\right)=0.5.$$

*The raw joint family generated by the derived products $\left(\xi_{i}|\upsilon_{j}\right)|\left(\xi_{i}|\upsilon_{j}\right)$ is (0,γ,ϵ,θ). Omitting the zero entry yields the associated joint partition (γ,ϵ,θ). The joint entropy evaluates to*

$$T_{\tau_{1}^{R}}^{2}\left((\Xi |\Upsilon )|(\Xi |\Upsilon )\right)=1-\left[{\left(1/4\right)}^{2}+{\left(1/2\right)}^{2}+{\left(1/4\right)}^{2}\right]=0.625.$$

*Since 0.625≤0.375+0.5=0.875, the subadditivity property established in Theorem 3 is explicitly satisfied.*

*2*. *Deviation from the Factorized Pseudo-Additive Model*

*Under the assumption of statistical independence, the predicted pseudo-additive joint entropy (Theorem 4) would evaluate to*

$$T_{\tau_{1}^{R}}^{2}\left(\Xi \right)+T_{\tau_{1}^{R}}^{2}\left(\Upsilon \right)+\left(1-\alpha \right)T_{\tau_{1}^{R}}^{2}\left(\Xi \right)T_{\tau_{1}^{R}}^{2}\left(\Upsilon \right)=0.375+0.5-\left(0.375\times 0.5\right)=0.6875.$$

*However, the internal factorization condition fails. For instance, $\tau_{1}^{R}\left((\gamma |\epsilon )|(\gamma |\epsilon )\right)=\tau_{1}^{R}\left(0\right)=0$, whereas the marginal product is $\tau_{1}^{R}\left(\gamma \right)\tau_{1}^{R}\left(\epsilon \right)=\left(1/4\right)\left(1/2\right)=0.125\neq 0$.*

*Because the state-induced joint distribution violates factorization, the actual joint entropy (0.625) deviates from the predicted value (0.6875). The resulting pseudo-additive residual (Definition 18) is*

$$\Delta_{\tau_{1}^{R}}^{2}\left(\Xi ,\Upsilon \right)=0.625-0.6875=-0.0625.$$

*This nonzero residual isolates the entropic deviation caused by the non-factorizing joint statistics.*

*3*. *Concavity with Respect to States*

*Evaluating the concavity inequality for Ξ=(γ,δ) under the convex combination $\tau_{\lambda }^{R}=0.5\tau_{1}^{R}+0.5\tau_{2}^{R}$ yields the combined state evaluations:*

$$\tau_{\lambda }^{R}\left(\gamma \right)=0.5\left(1/4\right)+0.5\left(1/6\right)=\frac{5}{24},\;\tau_{\lambda }^{R}\left(\delta \right)=0.5\left(3/4\right)+0.5\left(5/6\right)=\frac{19}{24}.$$

*The marginal entropy under $\tau_{2}^{R}$ is $T_{\tau_{2}^{R}}^{2}\left(\Xi \right)=1-\left(1/36+25/36\right)=10/36$. The linear combination of the individual entropies is*

$$0.5T_{\tau_{1}^{R}}^{2}\left(\Xi \right)+0.5T_{\tau_{2}^{R}}^{2}\left(\Xi \right)=0.5\left(\frac{6}{16}\right)+0.5\left(\frac{10}{36}\right)=\frac{94}{288}.$$

*Conversely, the entropy evaluated directly on the combined state is*

$$T_{\tau_{\lambda }^{R}}^{2}\left(\Xi \right)=1-\left[{\left(\frac{5}{24}\right)}^{2}+{\left(\frac{19}{24}\right)}^{2}\right]=1-\frac{386}{576}=\frac{95}{288}.$$

*Since 94/288≤95/288, the concavity inequality $\lambda T_{\tau_{1}^{R}}^{\alpha }\left(\Xi \right)+\left(1-\lambda \right)T_{\tau_{2}^{R}}^{\alpha }\left(\Xi \right)\leq T_{\tau_{\lambda }^{R}}^{\alpha }\left(\Xi \right)$ is confirmed.*

**Definition** **15.** *Let M=(M;∣) be a product Sheffer stroke basic algebra and $\tau^{R}:M\to \left[0,1\right]$ be a Riečan state on M. Let $\Xi =(\xi_{1},\ldots ,\xi_{k})$ and $\Upsilon =(\upsilon_{1},\ldots ,\upsilon_{l})$ be partitions in M. The conditional Tsallis entropy of order α, where α∈(0,1)∪(1,∞), of Ξ given Υ is defined as*

(146) $$T_{\tau^{R}}^{\alpha }\left(\Xi /\Upsilon \right)=\frac{1}{\alpha -1}\left(\sum_{j=1}^{l}\tau^{R}{\left(\upsilon_{j}\right)}^{\alpha }-\sum_{i=1}^{k}\sum_{j=1}^{l}\tau^{R}{\left(\left(\xi_{i}|\upsilon_{j}\right)|\left(\xi_{i}|\upsilon_{j}\right)\right)}^{\alpha }\right).$$

**Remark** **9.** *Fixing the entropic index at α=2 yields the conditional logical entropy of Ξ given Υ. At α=1, the value of $T_{\tau^{R}}^{\alpha }\left(\Xi /\Upsilon \right)$ assumes the indeterminate form 0/0. Applying L’Hôpital’s rule demonstrates that as α→1, the conditional Tsallis entropy converges to the conditional Shannon entropy $H_{\tau^{R}}\left(\Xi /\Upsilon \right)$ formulated via natural logarithms. Note that this convergence to the Shannon entropy expressed in nats is mathematically consistent with the standard parametric formulation of Tsallis statistics.*

Algorithm 4 calculates the Tsallis entropy and conditional Tsallis entropy for partitions in a product Sheffer stroke basic algebra. It takes a product Sheffer stroke basic algebra M, two partitions Ξ and Υ, a Riečan state operator $\tau^{R}$, and the entropic parameter α as inputs. It outputs the unconditional Tsallis entropy of Ξ and the conditional Tsallis entropy of Ξ given Υ.    
**Algorithm 4:** Calculation of Tsallis Entropy and Conditional Tsallis Entropy via Sheffer Stroke**Input**: Product Sheffer stroke basic algebra M=(M;∣), partition $\Xi =(\xi_{1},\ldots ,\xi_{k})$, partition $\Upsilon =(\upsilon_{1},\ldots ,\upsilon_{l})$, Riečan state operator $\tau^{R}:M\to \left[0,1\right]$, parameter α∈(0,1)∪(1,∞)**Output**: Tsallis entropy of Ξ, $T_{\tau^{R}}^{\alpha }\left(\Xi \right)$; Conditional Tsallis entropy of Ξ given Υ, $T_{\tau^{R}}^{\alpha }\left(\Xi /\Upsilon \right)$
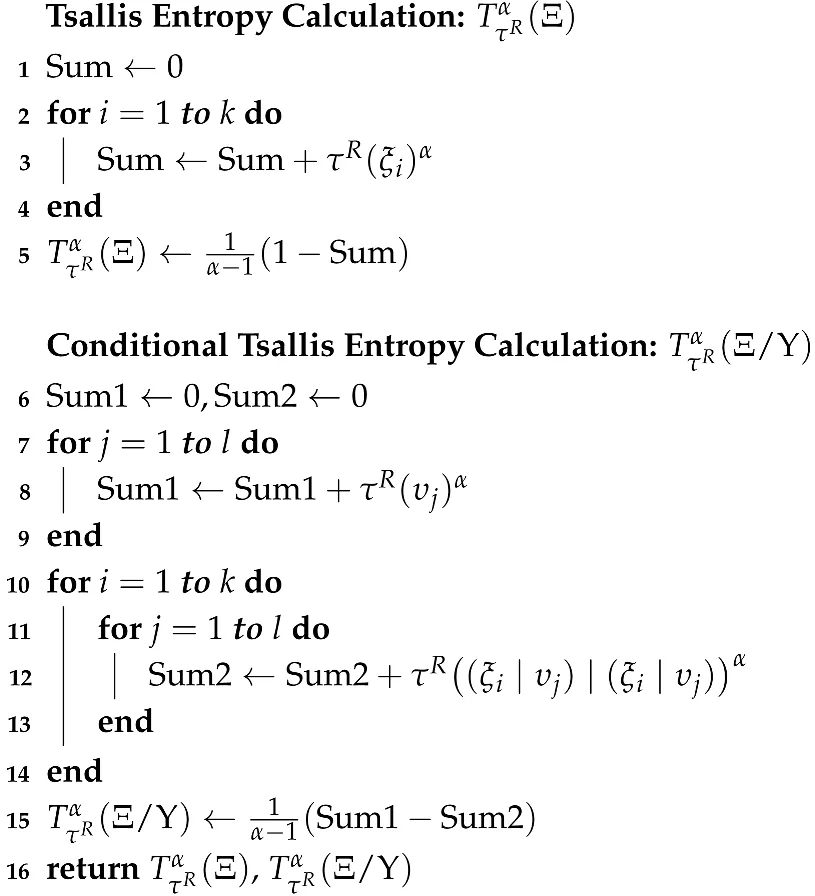


The computational procedure consists of two phases. The first phase computes the marginal Tsallis entropy of Ξ. It initializes a sum variable to zero and accumulates the α-power of the state values for each element $\xi_{i}$. The algorithm then applies the scaling factor to compute $T_{\tau^{R}}^{\alpha }\left(\Xi \right)$.

The second phase calculates the conditional Tsallis entropy $T_{\tau^{R}}^{\alpha }\left(\Xi /\Upsilon \right)$. It utilizes two accumulating variables. It computes the marginal state sum for the elements of Υ. A nested iteration then evaluates the joint elements of Ξ and Υ using the Sheffer stroke operation, calculating the state of $\left(\xi_{i}|\upsilon_{j}\right)|\left(\xi_{i}|\upsilon_{j}\right)$ for each pair. The conditional entropy is derived by computing the difference between these sums and applying the scaling factor.

Algorithm 4 implements the computation of the Tsallis and conditional Tsallis entropies without relying on external product operators. This ensures that these non-additive entropy measures can be calculated strictly within models of product Sheffer stroke basic algebras. The derived values provide measures of unconditional and conditional uncertainty for entropy-based analyses in structural logic frameworks.

A detailed computational complexity and scalability analysis of Algorithms 1–4 is provided in Appendix A.

**Theorem** **6.** *Let $\Xi =(\xi_{1},\ldots ,\xi_{k})$ and $\Upsilon =(\upsilon_{1},\ldots ,\upsilon_{l})$ be partitions in a product Sheffer stroke basic algebra B=(B;∣) equipped with a Riečan state $\tau^{R}:B\to \left[0,1\right]$. For the associated admissible Sheffer stroke joint partition satisfying the marginalization identities*

(147) $$\sum_{i=1}^{k}\tau^{R}\left(\left(\xi_{i}|\upsilon_{j}\right)|\left(\xi_{i}|\upsilon_{j}\right)\right)=\tau^{R}\left(\upsilon_{j}\right),\;j=1,\ldots ,l,$$

*the Tsallis entropy satisfies the chain rule:*

(148) $$T_{\tau^{R}}^{\alpha }\left((\Xi |\Upsilon )|(\Xi |\Upsilon )\right)=T_{\tau^{R}}^{\alpha }\left(\Xi /\Upsilon \right)+T_{\tau^{R}}^{\alpha }\left(\Upsilon \right),$$

*where (Ξ∣Υ)∣(Ξ∣Υ) denotes the admissible componentwise Sheffer stroke joint construction with components $\left(\xi_{i}|\upsilon_{j}\right)|\left(\xi_{i}|\upsilon_{j}\right)$.*

**Proof.**  By the definition of the joint Tsallis entropy for the partition (Ξ∣Υ)∣(Ξ∣Υ), we have:

(149) $$T_{\tau^{R}}^{\alpha }\left((\Xi |\Upsilon )|(\Xi |\Upsilon )\right)=\frac{1}{\alpha -1}\left(1-\sum_{i=1}^{k}\sum_{j=1}^{l}\tau^{R}{\left(\left(\xi_{i}|\upsilon_{j}\right)|\left(\xi_{i}|\upsilon_{j}\right)\right)}^{\alpha }\right).$$

Introducing the terms $\sum_{j=1}^{l}\tau^{R}{\left(\upsilon_{j}\right)}^{\alpha }-\sum_{j=1}^{l}\tau^{R}{\left(\upsilon_{j}\right)}^{\alpha }$ into the parenthesis yields

(150) $$T_{\tau^{R}}^{\alpha }\left((\Xi |\Upsilon )|(\Xi |\Upsilon )\right)=\frac{1}{\alpha -1}\left(1-\sum_{j=1}^{l}\tau^{R}{\left(\upsilon_{j}\right)}^{\alpha }+\sum_{j=1}^{l}\tau^{R}{\left(\upsilon_{j}\right)}^{\alpha }-\sum_{i=1}^{k}\sum_{j=1}^{l}\tau^{R}{\left(\left(\xi_{i}|\upsilon_{j}\right)|\left(\xi_{i}|\upsilon_{j}\right)\right)}^{\alpha }\right).$$

Splitting the expression yields

(151) $$\begin{array}{cc} T_{\tau^{R}}^{\alpha }\left((\Xi |\Upsilon )|(\Xi |\Upsilon )\right) & =\frac{1}{\alpha -1}\left(1-\sum_{j=1}^{l}\tau^{R}{\left(\upsilon_{j}\right)}^{\alpha }\right)\\ \; & \;+\frac{1}{\alpha -1}\left(\sum_{j=1}^{l}\tau^{R}{\left(\upsilon_{j}\right)}^{\alpha }-\sum_{i=1}^{k}\sum_{j=1}^{l}\tau^{R}{\left(\left(\xi_{i}|\upsilon_{j}\right)|\left(\xi_{i}|\upsilon_{j}\right)\right)}^{\alpha }\right). \end{array}$$

The first term corresponds to the marginal Tsallis entropy $T_{\tau^{R}}^{\alpha }\left(\Upsilon \right)$, and the second term corresponds to the conditional Tsallis entropy $T_{\tau^{R}}^{\alpha }\left(\Xi /\Upsilon \right)$ established in Definition 15. Therefore,

(152) $$T_{\tau^{R}}^{\alpha }\left((\Xi |\Upsilon )|(\Xi |\Upsilon )\right)=T_{\tau^{R}}^{\alpha }\left(\Upsilon \right)+T_{\tau^{R}}^{\alpha }\left(\Xi /\Upsilon \right).$$

This completes the proof.    □

**Example** **4.** *To validate the Chain Rule formulated in Theorem 6, we recall the product Sheffer stroke basic algebra M=(M;∣), the entropic index α=2, the partitions Ξ=(γ,δ) and Υ=(ϵ,ζ), and the Riečan state operator $\tau_{1}^{R}$ from Example 3.*

*The marginal state values for Υ are $\tau_{1}^{R}\left(\epsilon \right)=1/2$ and $\tau_{1}^{R}\left(\zeta \right)=1/2$. Consequently, the marginal Tsallis entropy of Υ evaluates to*

$$T_{\tau_{1}^{R}}^{2}\left(\Upsilon \right)=1-\left[\tau_{1}^{R}{\left(\epsilon \right)}^{2}+\tau_{1}^{R}{\left(\zeta \right)}^{2}\right]=1-\left(1/4+1/4\right)=0.5.$$

*Next, we calculate the conditional Tsallis entropy $T_{\tau_{1}^{R}}^{2}\left(\Xi /\Upsilon \right)$. The raw Sheffer stroke joint family derived via $\left(\xi_{i}|\upsilon_{j}\right)|\left(\xi_{i}|\upsilon_{j}\right)$ is (0,γ,ϵ,θ); after omitting the zero component, the associated admissible joint partition is (γ,ϵ,θ). Evaluating the sum of the squared state probabilities for these joint elements yields*

$$\sum_{i=1}^{2}\sum_{j=1}^{2}\tau_{1}^{R}{\left(\left(\xi_{i}|\upsilon_{j}\right)|\left(\xi_{i}|\upsilon_{j}\right)\right)}^{2}=0^{2}+{\left(1/4\right)}^{2}+{\left(1/2\right)}^{2}+{\left(1/4\right)}^{2}=\frac{6}{16}=0.375.$$

*Applying the conditional Tsallis entropy formula, we obtain*

$$T_{\tau_{1}^{R}}^{2}\left(\Xi /\Upsilon \right)=\left(\sum_{j=1}^{2}\tau_{1}^{R}{\left(\upsilon_{j}\right)}^{2}\right)-0.375=\left(1/4+1/4\right)-0.375=0.5-0.375=0.125.$$

*Finally, the joint Tsallis entropy for the combined partition (Ξ∣Υ)∣(Ξ∣Υ) was evaluated in Example 3 as*

$$T_{\tau_{1}^{R}}^{2}\left((\Xi |\Upsilon )|(\Xi |\Upsilon )\right)=1-0.375=0.625.$$

*Substituting these values into the chain rule identity from Theorem 6:*

$$\begin{array}{cc} T_{\tau_{1}^{R}}^{2}\left((\Xi |\Upsilon )|(\Xi |\Upsilon )\right) & =T_{\tau_{1}^{R}}^{2}\left(\Xi /\Upsilon \right)+T_{\tau_{1}^{R}}^{2}\left(\Upsilon \right)\\ 0.625 & =0.125+0.5 \end{array}$$

*This numerical calculation verifies the Tsallis entropy Chain Rule, demonstrating its consistency within the structure of the Sheffer stroke basic algebra.*

**Theorem** **7.** *Let $\Xi =(\xi_{1},\ldots ,\xi_{k})$ and $\Upsilon =(\upsilon_{1},\ldots ,\upsilon_{l})$ be partitions in a product Sheffer stroke basic algebra B=(B;∣), and let $\tau^{R}:B\to \left[0,1\right]$ be a Riečan state operator. The limiting behavior of the conditional Tsallis entropy as α→1 converges to the conditional Shannon entropy, expressed as*

(153) $$\lim_{\alpha \to 1}T_{\tau^{R}}^{\alpha }\left(\Xi /\Upsilon \right)=-\sum_{i=1}^{k}\sum_{j=1}^{l}\tau^{R}\left(\left(\xi_{i}|\upsilon_{j}\right)|\left(\xi_{i}|\upsilon_{j}\right)\right)\ln\frac{\tau^{R}\left(\left(\xi_{i}|\upsilon_{j}\right)|\left(\xi_{i}|\upsilon_{j}\right)\right)}{\tau^{R}\left(\upsilon_{j}\right)}.$$

**Proof.**  For any entropic index α∈(0,1)∪(1,∞), the conditional Tsallis entropy is defined over the structure of B as

(154) $$T_{\tau^{R}}^{\alpha }\left(\Xi /\Upsilon \right)=\frac{1}{\alpha -1}\left(\sum_{j=1}^{l}\tau^{R}{\left(\upsilon_{j}\right)}^{\alpha }-\sum_{i=1}^{k}\sum_{j=1}^{l}\tau^{R}{\left(\left(\xi_{i}|\upsilon_{j}\right)|\left(\xi_{i}|\upsilon_{j}\right)\right)}^{\alpha }\right)=\frac{f(\alpha )}{g(\alpha )},$$

where the functions *f* and *g* are defined on the domain (0,∞) by

(155) $$f\left(\alpha \right)=\sum_{j=1}^{l}\tau^{R}{\left(\upsilon_{j}\right)}^{\alpha }-\sum_{i=1}^{k}\sum_{j=1}^{l}\tau^{R}{\left(\left(\xi_{i}|\upsilon_{j}\right)|\left(\xi_{i}|\upsilon_{j}\right)\right)}^{\alpha },\;g\left(\alpha \right)=\alpha -1.$$

Both *f* and *g* are continuous and differentiable with respect to α. Evaluating the limits as α→1, we obtain $\lim_{\alpha \to 1}g\left(\alpha \right)=0$. For the numerator f(α), substituting the limit yields

(156) $$\lim_{\alpha \to 1}f\left(\alpha \right)=\sum_{j=1}^{l}\tau^{R}\left(\upsilon_{j}\right)-\sum_{i=1}^{k}\sum_{j=1}^{l}\tau^{R}\left(\left(\xi_{i}|\upsilon_{j}\right)|\left(\xi_{i}|\upsilon_{j}\right)\right).$$

By the additivity property of Riečan states over valid partitions, the marginal state of any element $\upsilon_{j}$ equals the summation of the joint states over all $\xi_{i}\in \Xi$. Thus, the identity $\sum_{i=1}^{k}\tau^{R}\left(\left(\xi_{i}|\upsilon_{j}\right)|\left(\xi_{i}|\upsilon_{j}\right)\right)=\tau^{R}\left(\upsilon_{j}\right)$ holds globally. Consequently,

(157) $$\lim_{\alpha \to 1}f\left(\alpha \right)=\sum_{j=1}^{l}\tau^{R}\left(\upsilon_{j}\right)-\sum_{j=1}^{l}\tau^{R}\left(\upsilon_{j}\right)=1-1=0.$$

Since the limit assumes the indeterminate form 0/0, we apply L’Hôpital’s rule:

(158) $$\lim_{\alpha \to 1}T_{\tau^{R}}^{\alpha }\left(\Xi /\Upsilon \right)=\lim_{\alpha \to 1}\frac{f^{\prime }\left(\alpha \right)}{g^{\prime }\left(\alpha \right)}.$$

Taking the first derivative of the denominator yields $g^{\prime }\left(\alpha \right)=1$. Utilizing the exponential derivative rule, the derivative of f(α) is computed as

(159) $$f^{\prime }\left(\alpha \right)=\sum_{j=1}^{l}\tau^{R}{\left(\upsilon_{j}\right)}^{\alpha }\ln\tau^{R}\left(\upsilon_{j}\right)-\sum_{i=1}^{k}\sum_{j=1}^{l}\tau^{R}{\left(\left(\xi_{i}|\upsilon_{j}\right)|\left(\xi_{i}|\upsilon_{j}\right)\right)}^{\alpha }\ln\tau^{R}\left(\left(\xi_{i}|\upsilon_{j}\right)|\left(\xi_{i}|\upsilon_{j}\right)\right).$$

Evaluating this explicit derivative exactly at the limit α→1, we obtain

(160) $$\lim_{\alpha \to 1}f^{\prime }\left(\alpha \right)=\sum_{j=1}^{l}\tau^{R}\left(\upsilon_{j}\right)\ln\tau^{R}\left(\upsilon_{j}\right)-\sum_{i=1}^{k}\sum_{j=1}^{l}\tau^{R}\left(\left(\xi_{i}|\upsilon_{j}\right)|\left(\xi_{i}|\upsilon_{j}\right)\right)\ln\tau^{R}\left(\left(\xi_{i}|\upsilon_{j}\right)|\left(\xi_{i}|\upsilon_{j}\right)\right).$$

To algebraically merge these terms, we reapply the state marginalization property, expanding the marginal state in the first term as $\tau^{R}\left(\upsilon_{j}\right)=\sum_{i=1}^{k}\tau^{R}\left(\left(\xi_{i}|\upsilon_{j}\right)|\left(\xi_{i}|\upsilon_{j}\right)\right)$. Substituting this expansion back into the limit equation gives

(161) $$\begin{array}{cc} \lim_{\alpha \to 1}f^{\prime }\left(\alpha \right) & =\sum_{i=1}^{k}\sum_{j=1}^{l}\tau^{R}\left(\left(\xi_{i}|\upsilon_{j}\right)|\left(\xi_{i}|\upsilon_{j}\right)\right)\ln\tau^{R}\left(\upsilon_{j}\right)\\ \; & \;-\sum_{i=1}^{k}\sum_{j=1}^{l}\tau^{R}\left(\left(\xi_{i}|\upsilon_{j}\right)|\left(\xi_{i}|\upsilon_{j}\right)\right)\ln\tau^{R}\left(\left(\xi_{i}|\upsilon_{j}\right)|\left(\xi_{i}|\upsilon_{j}\right)\right). \end{array}$$

Factoring out the common joint probability term and applying the logarithmic quotient rule (lnA−lnB=−ln(B/A)), we obtain the final expression:

(162) $$\lim_{\alpha \to 1}T_{\tau^{R}}^{\alpha }\left(\Xi /\Upsilon \right)=-\sum_{i=1}^{k}\sum_{j=1}^{l}\tau^{R}\left(\left(\xi_{i}|\upsilon_{j}\right)|\left(\xi_{i}|\upsilon_{j}\right)\right)\ln\frac{\tau^{R}\left(\left(\xi_{i}|\upsilon_{j}\right)|\left(\xi_{i}|\upsilon_{j}\right)\right)}{\tau^{R}\left(\upsilon_{j}\right)}.$$

This completes the proof.    □

**Example** **5.** *To validate the convergence behavior formulated in Theorem 7, we recall the product Sheffer stroke basic algebra M=(M;∣), the Riečan state operator $\tau_{1}^{R}$, and the partitions Ξ=(γ,δ) and Υ=(ϵ,ζ) established in Example 3.*

*According to Theorem 7, as the entropic index α→1, the conditional Tsallis entropy $T_{\tau_{1}^{R}}^{\alpha }\left(\Xi /\Upsilon \right)$ converges to the conditional Shannon entropy expressed as*

$$\lim_{\alpha \to 1}T_{\tau_{1}^{R}}^{\alpha }\left(\Xi /\Upsilon \right)=-\sum_{i=1}^{2}\sum_{j=1}^{2}\tau_{1}^{R}\left(\left(\xi_{i}|\upsilon_{j}\right)|\left(\xi_{i}|\upsilon_{j}\right)\right)\ln\frac{\tau_{1}^{R}\left(\left(\xi_{i}|\upsilon_{j}\right)|\left(\xi_{i}|\upsilon_{j}\right)\right)}{\tau_{1}^{R}\left(\upsilon_{j}\right)}.$$

*To perform this calculation, the marginal state values for the conditioning partition Υ are recalled from Example 3:*

$$\tau_{1}^{R}\left(\epsilon \right)=1/2,\;\tau_{1}^{R}\left(\zeta \right)=1/2.$$

*Next, we evaluate the joint elements generated by the operation $\left(\xi_{i}|\upsilon_{j}\right)|\left(\xi_{i}|\upsilon_{j}\right)$ and their corresponding probabilistic states under $\tau_{1}^{R}$. Applying the convention that 0ln0=0, the four combinatorial pairs are analyzed as follows:*

***Case*** ***1:*** *For $\xi_{1}=\gamma$ and $\upsilon_{1}=\epsilon$: The joint element is (γ∣ϵ)∣(γ∣ϵ)=1∣1=0. The state evaluates to $\tau_{1}^{R}\left(0\right)=0$, meaning the entropic contribution is 0.*

***Case*** ***2:*** *For $\xi_{2}=\delta$ and $\upsilon_{1}=\epsilon$: The joint element is (δ∣ϵ)∣(δ∣ϵ)=ζ∣ζ=ϵ. The state evaluates to $\tau_{1}^{R}\left(\epsilon \right)=1/2$. The contribution is*

$$\tau_{1}^{R}\left(\epsilon \right)\ln\frac{\tau_{1}^{R}\left(\epsilon \right)}{\tau_{1}^{R}\left(\epsilon \right)}=\frac{1}{2}\ln\left(\frac{1/2}{1/2}\right)=\frac{1}{2}\ln\left(1\right)=0.$$

***Case*** ***3:*** *For $\xi_{1}=\gamma$ and $\upsilon_{2}=\zeta$: The joint element is (γ∣ζ)∣(γ∣ζ)=δ∣δ=γ. The state evaluates to $\tau_{1}^{R}\left(\gamma \right)=1/4$. The contribution is*

$$\tau_{1}^{R}\left(\gamma \right)\ln\frac{\tau_{1}^{R}\left(\gamma \right)}{\tau_{1}^{R}\left(\zeta \right)}=\frac{1}{4}\ln\left(\frac{1/4}{1/2}\right)=\frac{1}{4}\ln\left(\frac{1}{2}\right)=-\frac{1}{4}\ln\left(2\right).$$

***Case*** ***4:*** *For $\xi_{2}=\delta$ and $\upsilon_{2}=\zeta$: The joint element is (δ∣ζ)∣(δ∣ζ)=η∣η=θ. The state evaluates to $\tau_{1}^{R}\left(\theta \right)=1/4$. The contribution is*

$$\tau_{1}^{R}\left(\theta \right)\ln\frac{\tau_{1}^{R}\left(\theta \right)}{\tau_{1}^{R}\left(\zeta \right)}=\frac{1}{4}\ln\left(\frac{1/4}{1/2}\right)=\frac{1}{4}\ln\left(\frac{1}{2}\right)=-\frac{1}{4}\ln\left(2\right).$$

*Aggregating these contributions and applying the negative sign of the formulation yields*

$$\lim_{\alpha \to 1}T_{\tau_{1}^{R}}^{\alpha }\left(\Xi /\Upsilon \right)=-\left(0+0-\frac{1}{4}\ln\left(2\right)-\frac{1}{4}\ln\left(2\right)\right)=-\left(-\frac{1}{2}\ln\left(2\right)\right)=\frac{1}{2}\ln\left(2\right).$$

*This evaluation ($\frac{1}{2}\ln\left(2\right)\approx 0.34657$) confirms the convergence of the conditional Tsallis entropy to the conditional Shannon entropy limit within the algebraic structure of M.*

**Theorem** **8.** *Let $\Xi =(\xi_{1},\ldots ,\xi_{k})$ be a partition in a product Sheffer stroke basic algebra B=(B;∣), and let $\tau^{R}:B\to \left[0,1\right]$ be a Riečan state operator. The limiting behavior of the unconditional Tsallis entropy of order α as α→1 converges to the Shannon entropy, expressed as*

(163) $$\lim_{\alpha \to 1}T_{\tau^{R}}^{\alpha }\left(\Xi \right)=-\sum_{i=1}^{k}\tau^{R}\left(\xi_{i}\right)\ln\tau^{R}\left(\xi_{i}\right).$$

**Proof.**  For any entropic index α∈(0,1)∪(1,∞), the unconditional Tsallis entropy of the partition Ξ is defined over B as

(164) $$T_{\tau^{R}}^{\alpha }\left(\Xi \right)=\frac{1}{\alpha -1}\left(1-\sum_{i=1}^{k}\tau^{R}{\left(\xi_{i}\right)}^{\alpha }\right)=\frac{f(\alpha )}{g(\alpha )},$$

where the functions *f* and *g* are defined on the domain (0,∞) by

(165) $$f\left(\alpha \right)=1-\sum_{i=1}^{k}\tau^{R}{\left(\xi_{i}\right)}^{\alpha },\;g\left(\alpha \right)=\alpha -1.$$

Both *f* and *g* are continuous and differentiable with respect to α. Evaluating the limit of the denominator as α→1, we obtain

(166) $$\lim_{\alpha \to 1}g\left(\alpha \right)=\lim_{\alpha \to 1}\left(\alpha -1\right)=0.$$

For the numerator f(α), substituting the limit and utilizing the normalization property of the Riečan state operator $\tau^{R}$ over partitions ($\sum_{i=1}^{k}\tau^{R}\left(\xi_{i}\right)=1$), we obtain

(167) $$\lim_{\alpha \to 1}f\left(\alpha \right)=1-\sum_{i=1}^{k}\tau^{R}\left(\xi_{i}\right)=1-1=0.$$

Since the quotient assumes the indeterminate form 0/0 as α→1, we apply L’Hôpital’s rule:

(168) $$\lim_{\alpha \to 1}T_{\tau^{R}}^{\alpha }\left(\Xi \right)=\lim_{\alpha \to 1}\frac{f^{\prime }\left(\alpha \right)}{g^{\prime }\left(\alpha \right)}.$$

Differentiating the denominator with respect to α yields $g^{\prime }\left(\alpha \right)=1$. Utilizing the exponential derivative rule, the first derivative of the numerator f(α) is

(169) $$f^{\prime }\left(\alpha \right)=\frac{d}{d\alpha }\left(1-\sum_{i=1}^{k}\tau^{R}{\left(\xi_{i}\right)}^{\alpha }\right)=-\sum_{i=1}^{k}\tau^{R}{\left(\xi_{i}\right)}^{\alpha }\ln\tau^{R}\left(\xi_{i}\right).$$

Evaluating this derivative at the limit α→1, we obtain

(170) $$\lim_{\alpha \to 1}f^{\prime }\left(\alpha \right)=-\sum_{i=1}^{k}\tau^{R}{\left(\xi_{i}\right)}^{1}\ln\tau^{R}\left(\xi_{i}\right)=-\sum_{i=1}^{k}\tau^{R}\left(\xi_{i}\right)\ln\tau^{R}\left(\xi_{i}\right).$$

Combining these limits into the quotient yields

(171) $$\lim_{\alpha \to 1}T_{\tau^{R}}^{\alpha }\left(\Xi \right)=\frac{\lim_{\alpha \to 1}f^{\prime }\left(\alpha \right)}{\lim_{\alpha \to 1}g^{\prime }\left(\alpha \right)}=\frac{-\sum_{i=1}^{k}\tau^{R}\left(\xi_{i}\right)\ln\tau^{R}\left(\xi_{i}\right)}{1}=-\sum_{i=1}^{k}\tau^{R}\left(\xi_{i}\right)\ln\tau^{R}\left(\xi_{i}\right).$$

This completes the proof.    □

**Definition** **16.** *Let $\Xi =(\xi_{1},\ldots ,\xi_{k})$, $\Upsilon =(\upsilon_{1},\ldots ,\upsilon_{l})$, and $\Omega =(\omega_{1},\ldots ,\omega_{m})$ be partitions in a product Sheffer stroke basic algebra B=(B;∣), and let $\tau^{R}:B\to \left[0,1\right]$ be a Riečan state operator. Define the bipartite Sheffer stroke refinement elements by*

(172) $$\zeta_{ij}=\left(\xi_{i}|\upsilon_{j}\right)|\left(\xi_{i}|\upsilon_{j}\right),\;\eta_{ir}=\left(\xi_{i}|\omega_{r}\right)|\left(\xi_{i}|\omega_{r}\right),\;\theta_{jr}=\left(\upsilon_{j}|\omega_{r}\right)|\left(\upsilon_{j}|\omega_{r}\right).$$

*The ordered triple (Ξ,Υ,Ω) is said to satisfy the triple Sheffer stroke marginalization condition, relative to $\tau^{R}$, if the sequential triple components*

(173) $$w_{ijr}=\left(\zeta_{ij}|\omega_{r}\right)|\left(\zeta_{ij}|\omega_{r}\right)$$

*are well defined under the fixed recursive Sheffer sum convention and their Riečan-state evaluations satisfy the following marginalization identities:*

(174) $$\begin{array}{cc} \sum_{i=1}^{k}\tau^{R}\left(w_{ijr}\right)\; & =\tau^{R}\left(\theta_{jr}\right),\;1\leq j\leq l,\;1\leq r\leq m, \end{array}$$

(175) $$\begin{array}{cc} \sum_{j=1}^{l}\tau^{R}\left(w_{ijr}\right)\; & =\tau^{R}\left(\eta_{ir}\right),\;1\leq i\leq k,\;1\leq r\leq m, \end{array}$$

(176) $$\begin{array}{cc} \sum_{r=1}^{m}\tau^{R}\left(w_{ijr}\right)\; & =\tau^{R}\left(\zeta_{ij}\right),\;1\leq i\leq k,\;1\leq j\leq l. \end{array}$$

*Equivalently, the state weights of the sequential triple Sheffer stroke refinement marginalize to the state weights of the corresponding bipartite Sheffer stroke refinements.*

**Remark** **10.** *The condition in Definition 16 is not a purely formal consequence of writing down the triple terms $w_{ijr}$. In a non-distributive Sheffer stroke setting, the sequentially constructed triple components may fail to reproduce the state values of the corresponding bipartite refinement after summation over one index.*

*For instance, suppose that for some fixed indices i and r the bipartite component*

(177) $$\eta_{ir}=\left(\xi_{i}|\omega_{r}\right)|\left(\xi_{i}|\omega_{r}\right)$$

*has positive state value, $\tau^{R}\left(\eta_{ir}\right)>0$, while the sequential triple components associated with the intermediate refinement by Υ are constrained by the algebra so that*

(178) $$w_{ijr}=\left(\zeta_{ij}|\omega_{r}\right)|\left(\zeta_{ij}|\omega_{r}\right)=0\;\mathrm{for}\;\mathrm{all}\;j=1,\ldots ,l.$$

*Then*

(179) $$\sum_{j=1}^{l}\tau^{R}\left(w_{ijr}\right)=0\neq \tau^{R}\left(\eta_{ir}\right),$$

*and the marginalization identity (175) fails. More generally, failure occurs whenever the recursive Sheffer stroke triple refinement does not preserve the state weights of the bipartite refinement under summation over the intermediate index. In such cases, the cancellation step used in the conditional chain-type identity is not justified, and Theorem 9 does not apply.*

**Theorem** **9.** *Let $\Xi =(\xi_{1},\ldots ,\xi_{k})$ and $\Upsilon =(\upsilon_{1},\ldots ,\upsilon_{l})$ be partitions in a product Sheffer stroke basic algebra B=(B;∣) equipped with a Riečan state operator $\tau^{R}:B\to \left[0,1\right]$. For any entropic index α>1, the Tsallis entropy satisfies the following properties:*

(*i*) *Non-negativity:*
(180) $$T_{\tau^{R}}^{\alpha }\left(\Xi /\Upsilon \right)\geq 0.$$
(*ii*) *Conditional chain-type rule: if $\Omega =(\omega_{1},\ldots ,\omega_{m})$ is a partition such that (Ξ,Υ,Ω) satisfies the triple Sheffer stroke marginalization condition of Definition 16, then*
(181) $$T_{\tau^{R}}^{\alpha }\left(((\Xi |\Upsilon )|(\Xi |\Upsilon ))/\Omega \right)=T_{\tau^{R}}^{\alpha }\left(\Xi /\Omega \right)+T_{\tau^{R}}^{\alpha }\left(\Upsilon /((\Xi |\Omega )|(\Xi |\Omega ))\right).$$
(*iii*) *Unconditional chain rule:*
(182) $$T_{\tau^{R}}^{\alpha }\left((\Xi |\Upsilon )|(\Xi |\Upsilon )\right)=T_{\tau^{R}}^{\alpha }\left(\Xi \right)+T_{\tau^{R}}^{\alpha }\left(\Upsilon /\Xi \right).$$
(*iv*) *Conditioning reduces entropy:*
(183) $$T_{\tau^{R}}^{\alpha }\left(\Xi /\Upsilon \right)\leq T_{\tau^{R}}^{\alpha }\left(\Xi \right).$$
(*v*) *Subadditivity:*
(184) $$T_{\tau^{R}}^{\alpha }\left((\Xi |\Upsilon )|(\Xi |\Upsilon )\right)\leq T_{\tau^{R}}^{\alpha }\left(\Xi \right)+T_{\tau^{R}}^{\alpha }\left(\Upsilon \right).$$

**Proof.**  The proof treats the five assertions sequentially. Non-negativity follows from the monotonicity of the state values under joint refinement; the conditional chain-type rule is obtained by expanding the triple refinement and using the triple Sheffer stroke marginalization condition to cancel the intermediate bipartite terms; the unconditional chain rule follows by direct cancellation of marginal power sums; conditioning-reduces-entropy is derived from the previously established Tsallis subadditivity estimate; and the final subadditivity statement follows by combining the unconditional chain rule with the conditioning-reduces-entropy property.

(*i*) Let

(185) $$\zeta_{ij}=\left(\xi_{i}|\upsilon_{j}\right)|\left(\xi_{i}|\upsilon_{j}\right).$$

By the marginalization property of the Sheffer stroke joint refinement and the additivity of Riečan states over orthogonal components, we have

(186) $$\tau^{R}\left(\upsilon_{j}\right)=\sum_{i=1}^{k}\tau^{R}\left(\zeta_{ij}\right),\;j=1,\ldots ,l.$$

The conditional Tsallis entropy is therefore expressed as

(187) $$T_{\tau^{R}}^{\alpha }\left(\Xi /\Upsilon \right)=\frac{1}{\alpha -1}\left(\sum_{j=1}^{l}\tau^{R}{\left(\upsilon_{j}\right)}^{\alpha }-\sum_{i=1}^{k}\sum_{j=1}^{l}\tau^{R}{\left(\zeta_{ij}\right)}^{\alpha }\right).$$

Using

(188) $$\tau^{R}{\left(\upsilon_{j}\right)}^{\alpha }=\tau^{R}{\left(\upsilon_{j}\right)}^{\alpha -1}\sum_{i=1}^{k}\tau^{R}\left(\zeta_{ij}\right),$$

we obtain

(189) $$T_{\tau^{R}}^{\alpha }\left(\Xi /\Upsilon \right)=\frac{1}{\alpha -1}\sum_{i=1}^{k}\sum_{j=1}^{l}\left(\tau^{R}\left(\zeta_{ij}\right)\tau^{R}{\left(\upsilon_{j}\right)}^{\alpha -1}-\tau^{R}{\left(\zeta_{ij}\right)}^{\alpha }\right).$$

Since $\zeta_{ij}$ is a joint refinement component lying below $\upsilon_{j}$ in the state order, monotonicity gives

(190) $$\tau^{R}\left(\zeta_{ij}\right)\leq \tau^{R}\left(\upsilon_{j}\right).$$

For α>1, the function $t\mapsto t^{\alpha -1}$ is increasing on [0,1], and hence

(191) $$\tau^{R}{\left(\zeta_{ij}\right)}^{\alpha -1}\leq \tau^{R}{\left(\upsilon_{j}\right)}^{\alpha -1}.$$

Therefore, each summand satisfies

(192) $$\tau^{R}\left(\zeta_{ij}\right)\tau^{R}{\left(\upsilon_{j}\right)}^{\alpha -1}-\tau^{R}{\left(\zeta_{ij}\right)}^{\alpha }=\tau^{R}\left(\zeta_{ij}\right)\left(\tau^{R}{\left(\upsilon_{j}\right)}^{\alpha -1}-\tau^{R}{\left(\zeta_{ij}\right)}^{\alpha -1}\right)\geq 0.$$

Since $\frac{1}{\alpha -1}>0$, it follows that

(193) $$T_{\tau^{R}}^{\alpha }\left(\Xi /\Upsilon \right)\geq 0.$$

(*ii*) Let $\Omega =(\omega_{1},\ldots ,\omega_{m})$ be a partition such that (Ξ,Υ,Ω) satisfies the triple Sheffer stroke marginalization condition of Definition 16. Put

(194) $$\zeta_{ij}=\left(\xi_{i}|\upsilon_{j}\right)|\left(\xi_{i}|\upsilon_{j}\right),\;w_{ijr}=\left(\zeta_{ij}|\omega_{r}\right)|\left(\zeta_{ij}|\omega_{r}\right).$$

By Definition 16, the sequential triple refinement components $w_{ijr}$ are well defined under the fixed recursive Sheffer sum convention and their Riečan-state evaluations marginalize to the corresponding bipartite refinement weights. In particular,

(195) $$\sum_{j=1}^{l}\tau^{R}\left(w_{ijr}\right)=\tau^{R}\left(\left(\xi_{i}|\omega_{r}\right)|\left(\xi_{i}|\omega_{r}\right)\right),$$

which is precisely the marginalization identity needed for the cancellation of the intermediate bipartite terms below.

Define

(196) $$p\left(\xi_{i},\upsilon_{j},\omega_{r}\right)=\tau^{R}\left(w_{ijr}\right)=\tau^{R}\left(\left(\zeta_{ij}|\omega_{r}\right)|\left(\zeta_{ij}|\omega_{r}\right)\right).$$

Then, by expanding the definitions of the two conditional entropy terms on the right-hand side, we get

(197) $$\begin{array}{cc} \; & T_{\tau^{R}}^{\alpha }\left(\Xi /\Omega \right)+T_{\tau^{R}}^{\alpha }\left(\Upsilon /((\Xi |\Omega )|(\Xi |\Omega ))\right)\\ \; & \;=\frac{1}{\alpha -1}\left(\sum_{r=1}^{m}\tau^{R}{\left(\omega_{r}\right)}^{\alpha }-\sum_{i=1}^{k}\sum_{r=1}^{m}\tau^{R}{\left(\left(\xi_{i}|\omega_{r}\right)|\left(\xi_{i}|\omega_{r}\right)\right)}^{\alpha }\right)\\ \; & \;+\frac{1}{\alpha -1}\left(\sum_{i=1}^{k}\sum_{r=1}^{m}\tau^{R}{\left(\left(\xi_{i}|\omega_{r}\right)|\left(\xi_{i}|\omega_{r}\right)\right)}^{\alpha }-\sum_{i=1}^{k}\sum_{j=1}^{l}\sum_{r=1}^{m}p{\left(\xi_{i},\upsilon_{j},\omega_{r}\right)}^{\alpha }\right). \end{array}$$

The intermediate bipartite terms cancel, yielding

(198) $$\frac{1}{\alpha -1}\left(\sum_{r=1}^{m}\tau^{R}{\left(\omega_{r}\right)}^{\alpha }-\sum_{i=1}^{k}\sum_{j=1}^{l}\sum_{r=1}^{m}p{\left(\xi_{i},\upsilon_{j},\omega_{r}\right)}^{\alpha }\right).$$

By the definition of conditional Tsallis entropy for the triple refinement over Ω, this expression is exactly

(199) $$T_{\tau^{R}}^{\alpha }\left(((\Xi |\Upsilon )|(\Xi |\Upsilon ))/\Omega \right).$$

Hence

(200) $$T_{\tau^{R}}^{\alpha }\left(((\Xi |\Upsilon )|(\Xi |\Upsilon ))/\Omega \right)=T_{\tau^{R}}^{\alpha }\left(\Xi /\Omega \right)+T_{\tau^{R}}^{\alpha }\left(\Upsilon /((\Xi |\Omega )|(\Xi |\Omega ))\right).$$

(*iii*) Again let

(201) $$\zeta_{ij}=\left(\xi_{i}|\upsilon_{j}\right)|\left(\xi_{i}|\upsilon_{j}\right).$$

By the definition of Tsallis entropy on the Sheffer stroke joint refinement,

(202) $$T_{\tau^{R}}^{\alpha }\left((\Xi |\Upsilon )|(\Xi |\Upsilon )\right)=\frac{1}{\alpha -1}\left(1-\sum_{i=1}^{k}\sum_{j=1}^{l}\tau^{R}{\left(\zeta_{ij}\right)}^{\alpha }\right).$$

Moreover,

(203) $$T_{\tau^{R}}^{\alpha }\left(\Xi \right)=\frac{1}{\alpha -1}\left(1-\sum_{i=1}^{k}\tau^{R}{\left(\xi_{i}\right)}^{\alpha }\right),$$

and, by the definition of conditional Tsallis entropy,

(204) $$T_{\tau^{R}}^{\alpha }\left(\Upsilon /\Xi \right)=\frac{1}{\alpha -1}\left(\sum_{i=1}^{k}\tau^{R}{\left(\xi_{i}\right)}^{\alpha }-\sum_{i=1}^{k}\sum_{j=1}^{l}\tau^{R}{\left(\zeta_{ij}\right)}^{\alpha }\right).$$

Adding the last two identities gives

(205) $$\begin{array}{cc} T_{\tau^{R}}^{\alpha }\left(\Xi \right)+T_{\tau^{R}}^{\alpha }\left(\Upsilon /\Xi \right)\; & =\frac{1}{\alpha -1}\left(1-\sum_{i=1}^{k}\sum_{j=1}^{l}\tau^{R}{\left(\zeta_{ij}\right)}^{\alpha }\right)\\ \; & =T_{\tau^{R}}^{\alpha }\left((\Xi |\Upsilon )|(\Xi |\Upsilon )\right). \end{array}$$

Thus,

(206) $$T_{\tau^{R}}^{\alpha }\left((\Xi |\Upsilon )|(\Xi |\Upsilon )\right)=T_{\tau^{R}}^{\alpha }\left(\Xi \right)+T_{\tau^{R}}^{\alpha }\left(\Upsilon /\Xi \right).$$

(*iv*) By Theorem 3, the joint and marginal state values for α>1 satisfy

(207) $$\sum_{i=1}^{k}\sum_{j=1}^{l}\tau^{R}{\left(\zeta_{ij}\right)}^{\alpha }\geq \sum_{i=1}^{k}\tau^{R}{\left(\xi_{i}\right)}^{\alpha }+\sum_{j=1}^{l}\tau^{R}{\left(\upsilon_{j}\right)}^{\alpha }-1.$$

Multiplying by −1 and rearranging gives

(208) $$\sum_{j=1}^{l}\tau^{R}{\left(\upsilon_{j}\right)}^{\alpha }-\sum_{i=1}^{k}\sum_{j=1}^{l}\tau^{R}{\left(\zeta_{ij}\right)}^{\alpha }\leq 1-\sum_{i=1}^{k}\tau^{R}{\left(\xi_{i}\right)}^{\alpha }.$$

Since α>1, multiplying by the positive factor $\frac{1}{\alpha -1}$ preserves the inequality:

(209) $$\frac{1}{\alpha -1}\left(\sum_{j=1}^{l}\tau^{R}{\left(\upsilon_{j}\right)}^{\alpha }-\sum_{i=1}^{k}\sum_{j=1}^{l}\tau^{R}{\left(\zeta_{ij}\right)}^{\alpha }\right)\leq \frac{1}{\alpha -1}\left(1-\sum_{i=1}^{k}\tau^{R}{\left(\xi_{i}\right)}^{\alpha }\right).$$

The left-hand side is $T_{\tau^{R}}^{\alpha }\left(\Xi /\Upsilon \right)$ and the right-hand side is $T_{\tau^{R}}^{\alpha }\left(\Xi \right)$. Hence,

(210) $$T_{\tau^{R}}^{\alpha }\left(\Xi /\Upsilon \right)\leq T_{\tau^{R}}^{\alpha }\left(\Xi \right).$$

(*v*) By the unconditional chain rule established in (iii),

(211) $$T_{\tau^{R}}^{\alpha }\left((\Xi |\Upsilon )|(\Xi |\Upsilon )\right)=T_{\tau^{R}}^{\alpha }\left(\Xi \right)+T_{\tau^{R}}^{\alpha }\left(\Upsilon /\Xi \right).$$

Applying the conditioning-reduces-entropy property from (iv) to the pair (Υ,Ξ) gives

(212) $$T_{\tau^{R}}^{\alpha }\left(\Upsilon /\Xi \right)\leq T_{\tau^{R}}^{\alpha }\left(\Upsilon \right).$$

Therefore,

(213) $$T_{\tau^{R}}^{\alpha }\left((\Xi |\Upsilon )|(\Xi |\Upsilon )\right)\leq T_{\tau^{R}}^{\alpha }\left(\Xi \right)+T_{\tau^{R}}^{\alpha }\left(\Upsilon \right).$$

This proves the subadditivity property.    □

**Theorem** **10.** *Let $\Xi =(\xi_{1},\ldots ,\xi_{k})$ and $\Upsilon =(\upsilon_{1},\ldots ,\upsilon_{l})$ be partitions in a product Sheffer stroke basic algebra B=(B;∣). If Ξ and Υ are statistically independent with respect to the Riečan state $\tau^{R}$, meaning that the joint Riečan-state values satisfy $\tau^{R}\left(\left(\xi_{i}|\upsilon_{j}\right)|\left(\xi_{i}|\upsilon_{j}\right)\right)=\tau^{R}\left(\xi_{i}\right)\tau^{R}\left(\upsilon_{j}\right)$ for all i and j, then the conditional Tsallis entropy evaluates to*

(214) $$T_{\tau^{R}}^{\alpha }\left(\Xi /\Upsilon \right)=T_{\tau^{R}}^{\alpha }\left(\Xi \right)+\left(1-\alpha \right)T_{\tau^{R}}^{\alpha }\left(\Xi \right)T_{\tau^{R}}^{\alpha }\left(\Upsilon \right).$$

**Proof.**  The proof starts from the definition of conditional Tsallis entropy and uses statistical independence to factor the joint α-power sum into a product of the marginal α-power sums. These marginal sums are then rewritten in terms of the corresponding unconditional Tsallis entropies, yielding the stated pseudo-additive conditional identity by direct algebraic simplification.

By the definition established in Definition 15, the conditional Tsallis entropy is given by

(215) $$T_{\tau^{R}}^{\alpha }\left(\Xi /\Upsilon \right)=\frac{1}{\alpha -1}\left(\sum_{j=1}^{l}\tau^{R}{\left(\upsilon_{j}\right)}^{\alpha }-\sum_{i=1}^{k}\sum_{j=1}^{l}\tau^{R}{\left(\left(\xi_{i}|\upsilon_{j}\right)|\left(\xi_{i}|\upsilon_{j}\right)\right)}^{\alpha }\right).$$

Under the hypothesis of statistical independence, we substitute the multiplicative identity into the joint state term:

(216) $$T_{\tau^{R}}^{\alpha }\left(\Xi /\Upsilon \right)=\frac{1}{\alpha -1}\left(\sum_{j=1}^{l}\tau^{R}{\left(\upsilon_{j}\right)}^{\alpha }-\sum_{i=1}^{k}\sum_{j=1}^{l}{\left(\tau^{R}\left(\xi_{i}\right)\tau^{R}\left(\upsilon_{j}\right)\right)}^{\alpha }\right).$$

Factoring the double summation yields

(217) $$T_{\tau^{R}}^{\alpha }\left(\Xi /\Upsilon \right)=\frac{1}{\alpha -1}\left(\sum_{j=1}^{l}\tau^{R}{\left(\upsilon_{j}\right)}^{\alpha }-\left(\sum_{i=1}^{k}\tau^{R}{\left(\xi_{i}\right)}^{\alpha }\right)\left(\sum_{j=1}^{l}\tau^{R}{\left(\upsilon_{j}\right)}^{\alpha }\right)\right).$$

Extracting the common marginal summation term $\sum_{j=1}^{l}\tau^{R}{\left(\upsilon_{j}\right)}^{\alpha }$ gives

(218) $$T_{\tau^{R}}^{\alpha }\left(\Xi /\Upsilon \right)=\frac{1}{\alpha -1}\left(\sum_{j=1}^{l}\tau^{R}{\left(\upsilon_{j}\right)}^{\alpha }\right)\left(1-\sum_{i=1}^{k}\tau^{R}{\left(\xi_{i}\right)}^{\alpha }\right).$$

From the definition of the unconditional Tsallis entropy $T_{\tau^{R}}^{\alpha }\left(\Xi \right)$, we have $1-\sum_{i=1}^{k}\tau^{R}{\left(\xi_{i}\right)}^{\alpha }=\left(\alpha -1\right)T_{\tau^{R}}^{\alpha }\left(\Xi \right)$. Substituting this directly into the equation simplifies the expression to

(219) $$T_{\tau^{R}}^{\alpha }\left(\Xi /\Upsilon \right)=\left(\sum_{j=1}^{l}\tau^{R}{\left(\upsilon_{j}\right)}^{\alpha }\right)T_{\tau^{R}}^{\alpha }\left(\Xi \right).$$

Furthermore, rearranging the unconditional entropy definition for the partition Υ expresses the remaining sum as $\sum_{j=1}^{l}\tau^{R}{\left(\upsilon_{j}\right)}^{\alpha }=1-\left(\alpha -1\right)T_{\tau^{R}}^{\alpha }\left(\Upsilon \right)$. Substituting this identity completes the algebraic derivation:

(220) $$T_{\tau^{R}}^{\alpha }\left(\Xi /\Upsilon \right)=\left(1-\left(\alpha -1\right)T_{\tau^{R}}^{\alpha }\left(\Upsilon \right)\right)T_{\tau^{R}}^{\alpha }\left(\Xi \right)=T_{\tau^{R}}^{\alpha }\left(\Xi \right)-\left(\alpha -1\right)T_{\tau^{R}}^{\alpha }\left(\Xi \right)T_{\tau^{R}}^{\alpha }\left(\Upsilon \right).$$

Distributing the negative sign results in the pseudo-additive relation:

(221) $$T_{\tau^{R}}^{\alpha }\left(\Xi /\Upsilon \right)=T_{\tau^{R}}^{\alpha }\left(\Xi \right)+\left(1-\alpha \right)T_{\tau^{R}}^{\alpha }\left(\Xi \right)T_{\tau^{R}}^{\alpha }\left(\Upsilon \right).$$

This completes the proof.    □

**Remark** **11.** *Theorem 10 highlights a theoretical divergence between Tsallis and Shannon information structures within Sheffer stroke basic algebras. In classical Shannon entropy (the extensive limit where α→1), statistical independence implies $H_{\tau^{R}}\left(\Xi /\Upsilon \right)=H_{\tau^{R}}\left(\Xi \right)$, meaning that knowing Υ provides no additional information regarding Ξ. However, for Tsallis entropy, the non-extensivity parameter α>1 leaves a residual algebraic term, namely $\left(1-\alpha \right)T_{\tau^{R}}^{\alpha }\left(\Xi \right)T_{\tau^{R}}^{\alpha }\left(\Upsilon \right)$. This demonstrates that, for Tsallis entropy, the conditional expression retains a non-extensive pseudo-additive correction term even under the state factorization condition. This term should not be interpreted as a statistical correlation in the classical Shannon sense, but as a consequence of the non-additive Tsallis composition law.*

**Definition** **17.** *Let Ξ and Υ be partitions in a product Sheffer stroke basic algebra B=(B;∣) equipped with a Riečan state $\tau^{R}:B\to \left[0,1\right]$. For an entropic index α>1, the Tsallis Mutual Information, denoted by $I_{\tau^{R}}^{\alpha }\left(\Xi ;\Upsilon \right)$, is defined as the following entropy-balance functional:*

(222) $$I_{\tau^{R}}^{\alpha }\left(\Xi ;\Upsilon \right)=T_{\tau^{R}}^{\alpha }\left(\Xi \right)+T_{\tau^{R}}^{\alpha }\left(\Upsilon \right)-T_{\tau^{R}}^{\alpha }\left((\Xi |\Upsilon )|(\Xi |\Upsilon )\right),$$

*where the application of the Sheffer stroke to the partitions Ξ and Υ is understood componentwise, generating the associated admissible joint refinement components*

(223) $$\zeta_{ij}=\left(\xi_{i}|\upsilon_{j}\right)|\left(\xi_{i}|\upsilon_{j}\right).$$

**Definition** **18.** *Let Ξ and Υ be partitions in a product Sheffer stroke basic algebra B=(B;∣), and let $\tau^{R}:B\to \left[0,1\right]$ be a Riečan state. For α>1, the Tsallis pseudo-additive residual associated with the pair (Ξ,Υ) is defined by*

(224) $$\Delta_{\tau^{R}}^{\alpha }\left(\Xi ,\Upsilon \right)=T_{\tau^{R}}^{\alpha }\left((\Xi |\Upsilon )|(\Xi |\Upsilon )\right)-T_{\tau^{R}}^{\alpha }\left(\Xi \right)-T_{\tau^{R}}^{\alpha }\left(\Upsilon \right)-\left(1-\alpha \right)T_{\tau^{R}}^{\alpha }\left(\Xi \right)T_{\tau^{R}}^{\alpha }\left(\Upsilon \right).$$

*Equivalently, $\Delta_{\tau^{R}}^{\alpha }\left(\Xi ,\Upsilon \right)$ measures the deviation of the actual joint Tsallis entropy from the pseudo-additive value predicted by statistical factorization. Hence, $\Delta_{\tau^{R}}^{\alpha }\left(\Xi ,\Upsilon \right)=0$ whenever the joint state distribution factorizes as*

(225) $$\tau^{R}\left(\left(\xi_{i}|\upsilon_{j}\right)|\left(\xi_{i}|\upsilon_{j}\right)\right)=\tau^{R}\left(\xi_{i}\right)\tau^{R}\left(\upsilon_{j}\right)$$

*for all i and j. A nonzero residual detects a deviation from the factorized pseudo-additive Tsallis formula. However, the converse direction is not asserted: the scalar equality $\Delta_{\tau^{R}}^{\alpha }\left(\Xi ,\Upsilon \right)=0$ is not claimed, without additional identifiability assumptions, to give a complete componentwise characterization of statistical independence.*

**Proposition** **9.** *Let Ξ and Υ be partitions in a product Sheffer stroke basic algebra B=(B;∣) equipped with a Riečan state $\tau^{R}$. If *

(226) $$\Delta_{\tau^{R}}^{\alpha }\left(\Xi ,\Upsilon \right)\neq 0,$$

*then the state values induced on the associated Sheffer stroke joint refinement do not satisfy the pseudo-additive Tsallis formula predicted by the factorized model determined by the marginal partitions. Consequently, the residual $\Delta_{\tau^{R}}^{\alpha }\left(\Xi ,\Upsilon \right)$ provides a state-dependent algebraic indicator of deviation from factorized joint statistics within the present framework.*

**Proof.**  By Definition 18, the value $\Delta_{\tau^{R}}^{\alpha }\left(\Xi ,\Upsilon \right)$ is the difference between the actual joint Tsallis entropy and the pseudo-additive entropy obtained from the factorized state model. If the factorization condition

(227) $$\tau^{R}\left(\left(\xi_{i}|\upsilon_{j}\right)|\left(\xi_{i}|\upsilon_{j}\right)\right)=\tau^{R}\left(\xi_{i}\right)\tau^{R}\left(\upsilon_{j}\right)$$

holds for all joint components, Theorem 4 implies

(228) $$\Delta_{\tau^{R}}^{\alpha }\left(\Xi ,\Upsilon \right)=0.$$

Therefore, a nonzero residual rules out the factorized pseudo-additive description at the Tsallis entropy level. This conclusion is purely algebraic and state-dependent; it is not an operational contextuality statement.    □

**Theorem** **11.** *Let Ξ and Υ be partitions in a product Sheffer stroke basic algebra B=(B;∣) equipped with a Riečan state $\tau^{R}:B\to \left[0,1\right]$. For every entropic index α>1, the Tsallis mutual information $I_{\tau^{R}}^{\alpha }\left(\Xi ;\Upsilon \right)$ satisfies the following properties:*

(*i*) *Symmetry:*
$$I_{\tau^{R}}^{\alpha }\left(\Xi ;\Upsilon \right)=I_{\tau^{R}}^{\alpha }\left(\Upsilon ;\Xi \right).$$
(*ii*) *Entropy reduction:*
$$I_{\tau^{R}}^{\alpha }\left(\Xi ;\Upsilon \right)=T_{\tau^{R}}^{\alpha }\left(\Xi \right)-T_{\tau^{R}}^{\alpha }\left(\Xi /\Upsilon \right).$$
(*iii*) *Non-negativity:*
$$I_{\tau^{R}}^{\alpha }\left(\Xi ;\Upsilon \right)\geq 0.$$
(*iv*) *Independent case: If Ξ and Υ are statistically independent with respect to $\tau^{R}$ in the sense of Definition 14, then*
$$I_{\tau^{R}}^{\alpha }\left(\Xi ;\Upsilon \right)=\left(\alpha -1\right)T_{\tau^{R}}^{\alpha }\left(\Xi \right)T_{\tau^{R}}^{\alpha }\left(\Upsilon \right).$$

**Proof.**  The proof proceeds from the defining identity of Tsallis mutual information. Symmetry follows from the commutativity of the Sheffer stroke and permutation invariance of Tsallis entropy; the entropy reduction formula is obtained by substituting the Tsallis chain rule; non-negativity follows directly from Tsallis subadditivity; and the independent case is derived by inserting the conditional independence formula into the entropy reduction identity.

Let

$$\Xi =\left(\xi_{1},\ldots ,\xi_{k}\right),\;\Upsilon =\left(\upsilon_{1},\ldots ,\upsilon_{l}\right).$$

By Definition 17,

(229) $$I_{\tau^{R}}^{\alpha }\left(\Xi ;\Upsilon \right)=T_{\tau^{R}}^{\alpha }\left(\Xi \right)+T_{\tau^{R}}^{\alpha }\left(\Upsilon \right)-T_{\tau^{R}}^{\alpha }\left((\Xi |\Upsilon )|(\Xi |\Upsilon )\right).$$

(*i*) Since the Sheffer stroke operation is commutative, for every pair of indices (i,j) we have

$$\left(\xi_{i}|\upsilon_{j}\right)|\left(\xi_{i}|\upsilon_{j}\right)=\left(\upsilon_{j}|\xi_{i}\right)|\left(\upsilon_{j}|\xi_{i}\right).$$

Thus, the joint partitions generated by

(Ξ∣Υ)∣(Ξ∣Υ)and(Υ∣Ξ)∣(Υ∣Ξ)

coincide up to a permutation of their nonzero components. Since Tsallis entropy is invariant under permutations of partition components, it follows that

$$T_{\tau^{R}}^{\alpha }\left((\Xi |\Upsilon )|(\Xi |\Upsilon )\right)=T_{\tau^{R}}^{\alpha }\left((\Upsilon |\Xi )|(\Upsilon |\Xi )\right).$$

Combining this identity with (229) gives

$$I_{\tau^{R}}^{\alpha }\left(\Xi ;\Upsilon \right)=I_{\tau^{R}}^{\alpha }\left(\Upsilon ;\Xi \right).$$

(*ii*) By the chain rule established in Theorem 6,

(230) $$T_{\tau^{R}}^{\alpha }\left((\Xi |\Upsilon )|(\Xi |\Upsilon )\right)=T_{\tau^{R}}^{\alpha }\left(\Upsilon \right)+T_{\tau^{R}}^{\alpha }\left(\Xi /\Upsilon \right).$$

Substituting (230) into (229) yields

(231) $$\begin{array}{cc} I_{\tau^{R}}^{\alpha }\left(\Xi ;\Upsilon \right)\; & =T_{\tau^{R}}^{\alpha }\left(\Xi \right)+T_{\tau^{R}}^{\alpha }\left(\Upsilon \right)-\left[T_{\tau^{R}}^{\alpha }\left(\Upsilon \right)+T_{\tau^{R}}^{\alpha }\left(\Xi /\Upsilon \right)\right]\\ \; & =T_{\tau^{R}}^{\alpha }\left(\Xi \right)-T_{\tau^{R}}^{\alpha }\left(\Xi /\Upsilon \right). \end{array}$$

This proves the entropy reduction identity.

(*iii*) By the subadditivity of Tsallis entropy for joint Sheffer stroke refinements, established in Theorem 3, we have

$$T_{\tau^{R}}^{\alpha }\left((\Xi |\Upsilon )|(\Xi |\Upsilon )\right)\leq T_{\tau^{R}}^{\alpha }\left(\Xi \right)+T_{\tau^{R}}^{\alpha }\left(\Upsilon \right).$$

Substituting this inequality into Definition 17 immediately gives

$$I_{\tau^{R}}^{\alpha }\left(\Xi ;\Upsilon \right)\geq 0.$$

(*iv*) Assume that Ξ and Υ are statistically independent with respect to $\tau^{R}$. By Theorem 10,

(232) $$T_{\tau^{R}}^{\alpha }\left(\Xi /\Upsilon \right)=T_{\tau^{R}}^{\alpha }\left(\Xi \right)+\left(1-\alpha \right)T_{\tau^{R}}^{\alpha }\left(\Xi \right)T_{\tau^{R}}^{\alpha }\left(\Upsilon \right).$$

Using the entropy reduction identity proved in part *(ii)* and substituting (232), we obtain

(233) $$\begin{array}{cc} I_{\tau^{R}}^{\alpha }\left(\Xi ;\Upsilon \right)\; & =T_{\tau^{R}}^{\alpha }\left(\Xi \right)-T_{\tau^{R}}^{\alpha }\left(\Xi /\Upsilon \right)\\ \; & =T_{\tau^{R}}^{\alpha }\left(\Xi \right)-\left[T_{\tau^{R}}^{\alpha }\left(\Xi \right)+\left(1-\alpha \right)T_{\tau^{R}}^{\alpha }\left(\Xi \right)T_{\tau^{R}}^{\alpha }\left(\Upsilon \right)\right]\\ \; & =\left(\alpha -1\right)T_{\tau^{R}}^{\alpha }\left(\Xi \right)T_{\tau^{R}}^{\alpha }\left(\Upsilon \right). \end{array}$$

This completes the proof.    □

To illustrate how the abstract mathematical framework may be compared with standard quantum-logical terminology, we now present a finite-dimensional interpretive example. The example is intended only as an algebraic analogy and not as a representation theorem or a physical implementation model. The example below shows how partitions, Sheffer stroke operations, and state-based entropies can be interpreted as algebraic counterparts of measurement contexts for a single four-level quantum system, without requiring composite tensor-product spaces.

**Example** **6.** *As an interpretive analogy, consider the notation of a four-outcome finite-dimensional quantum system with orthonormal basis labels*

(234) |1〉,|2〉,|3〉,|4〉.

*In a concrete Hilbert-space model, a projective measurement in such a basis would yield four mutually exclusive outcomes. At an illustrative level, let*

(235) $$\Xi =(\xi_{1},\xi_{2},\xi_{3},\xi_{4})$$

*be a partition in a product Sheffer stroke basic algebra B=(B;∣), where the elements $\xi_{1},\xi_{2},\xi_{3},\xi_{4}$ are regarded as algebraic representatives of four elementary alternatives. In a concrete Hilbert-space projection model, these alternatives could correspond to the four basis outcomes.*

*The partition condition*

(236) $$\overset{4}{\underset{i=1}{⨁}}\xi_{i}=1$$

*expresses that the four algebraic alternatives form a complete finite context; in a concrete projection model, this would correspond to a complete projection-valued decomposition, often interpreted as a measurement context. In this interpretation, the greatest element 1 may be regarded as certainty, while the terms $\xi_{i}|\xi_{i}$ play the role of complement-like algebraic counterparts of the elementary outcomes within the Sheffer stroke formalism.*

*To illustrate the refinement mechanism, consider also the coarser partition*

(237) $$\Upsilon =(\upsilon_{1},\upsilon_{2}),$$

*where $\upsilon_{1}$ represents the coarse-grained alternative that the outcome lies in {|1〉,|2〉}, and $\upsilon_{2}$ represents the alternative that the outcome lies in {|3〉,|4〉}. Algebraically, this coarse graining may be expressed as*

(238) $$\upsilon_{1}=\xi_{1}⊕\xi_{2},\;\upsilon_{2}=\xi_{3}⊕\xi_{4}.$$

*In the algebraic language, the joint partition*

(239) (Ξ∣Υ)∣(Ξ∣Υ)

*is understood componentwise as having elements*

(240) $$\zeta_{ij}=\left(\xi_{i}|\upsilon_{j}\right)|\left(\xi_{i}|\upsilon_{j}\right),\;i=1,\ldots ,4,\;j=1,2.$$

*These elements represent algebraic joint refinement components associated with the partitions Ξ and Υ. They should not be interpreted as operational joint measurement events unless an additional representation in a concrete measurement model is supplied. Since the fine-grained partition Ξ refines the coarser context Υ, the componentwise joint refinement terms yield the elements of Ξ together with null components; after omitting the null components, the resulting partition coincides with Ξ. More explicitly, at the level of the algebraic illustration, the non-null components are*

(241) $$\zeta_{11}=\xi_{1},\;\zeta_{21}=\xi_{2},\;\zeta_{32}=\xi_{3},\;\zeta_{42}=\xi_{4},$$

*while the remaining components are null. This illustrates how the derived term*

(242) (x∣y)∣(x∣y)

*acts as an internal joint refinement term within the Sheffer stroke basic algebra.*

*Let $\tau^{R}:B\to \left[0,1\right]$ be a Riečan state. In this interpretation, we assign*

(243) $$\tau^{R}\left(\xi_{1}\right)=p_{1},\;\tau^{R}\left(\xi_{2}\right)=p_{2},\;\tau^{R}\left(\xi_{3}\right)=p_{3},\;\tau^{R}\left(\xi_{4}\right)=p_{4},$$

*where*

(244) $$p_{1}+p_{2}+p_{3}+p_{4}=1.$$

*These values are probability-type weights assigned to the four algebraic alternatives. In a concrete Hilbert-space projection model, such weights would correspond to probabilities of measurement outcomes, but no such representation is assumed here. The Shannon entropy of the partition Ξ is then*

(245) $$H_{\tau^{R}}\left(\Xi \right)=-\sum_{i=1}^{4}p_{i}\log p_{i},$$

*the logical entropy is*

(246) $$H_{\tau^{R}}^{l}\left(\Xi \right)=\sum_{i=1}^{4}\left(p_{i}-p_{i}^{2}\right)=1-\sum_{i=1}^{4}p_{i}^{2},$$

*and the Tsallis entropy of order α∈(0,1)∪(1,∞) is*

(247) $$T_{\tau^{R}}^{\alpha }\left(\Xi \right)=\frac{1}{\alpha -1}\left(1-\sum_{i=1}^{4}p_{i}^{\alpha }\right).$$

*In the uniform case*

(248) $$p_{1}=p_{2}=p_{3}=p_{4}=\frac{1}{4},$$

*one obtains*

(249) $$H_{\tau^{R}}\left(\Xi \right)=\log4,\;H_{\tau^{R}}^{l}\left(\Xi \right)=\frac{3}{4},\;T_{\tau^{R}}^{\alpha }\left(\Xi \right)=\frac{1}{\alpha -1}\left(1-4^{1-\alpha }\right).$$

*Hence, within this algebraic interpretation, product Sheffer stroke basic algebras supply an abstract event-like structure in which partitions describe finite contexts for state-based entropy calculations, the derived terms*

(250) (x∣y)∣(x∣y)

*encode algebraic joint refinement components, and the Riečan state supplies the probability-type weights entering the entropy functionals studied in this paper.*

To verify that the proposed entropy constructions are not restricted to small illustrative algebras, larger-scale numerical simulations on finite state-induced Sheffer stroke joint refinements are provided in Appendix B.

For clarity, Table 4 summarizes the three entropy functionals considered in this paper. The comparison highlights their algebraic definitions, principal properties, advantages, and limitations within the setting of product Sheffer stroke basic algebras equipped with Riečan states.

As shown in Table 4, Shannon entropy serves as the extensive reference case, while logical entropy appears as the quadratic member of the Tsallis family. The Tsallis entropy therefore provides the most flexible framework among the three since the entropic index α allows one to tune the sensitivity of the entropy functional. In the present algebraic setting, this flexibility is particularly important because the pseudo-additive residual associated with Tsallis entropy records deviations from the factorized Tsallis pseudo-additive model at the level of admissible Sheffer stroke joint state values.

### Algebraic Quantum-Logical Interpretation and Its Scope

The algebraic structures developed in this study can be compared, at an interpretive level, with certain forms of non-classical logical terminology used in quantum theory. Orthomodular lattices provide the standard lattice-theoretic formalism for quantum propositions, whereas product Sheffer stroke basic algebras form a distinct algebraic setting based on a commutative primitive stroke operation. The purpose of the following discussion is therefore only to clarify possible analogies and differences, not to assert a general inclusion or representation theorem.

To address this point explicitly, we describe an interpretive quantum-logical analogy for the Sheffer stroke basic algebra B=(B;∣). The purpose of this analogy is not to claim that every product Sheffer stroke basic algebra is canonically representable as a Hilbert-space projection lattice, but to indicate how some of the algebraic ingredients may be compared with standard quantum-logical terminology under additional representation assumptions.

It is important to explicitly delineate the algebraic scope of this comparison. In orthomodular structures, operations such as the Sasaki projection or the Sasaki arrow are often used to describe non-classical implication-like or projection-like behavior. These operations are generally non-commutative. Therefore, they cannot be identified with the Sheffer stroke operation used in the present paper since the Sheffer stroke operation in a Sheffer stroke basic algebra is required to satisfy the commutativity condition

(251) x∣y=y∣x.

Consequently, product Sheffer stroke basic algebras should not be described as universally containing all orthomodular lattices as a subclass. The present framework is instead treated as a distinct algebraic setting for studying entropy on finite non-distributive structures equipped with a commutative primitive stroke operation and Riečan states.

Accordingly, any connection between the present framework and standard quantum-logical models should be understood as interpretive and representation-theoretic rather than as an established general inclusion theorem. Establishing precise representation results between product Sheffer stroke basic algebras and orthomodular lattices, orthomodular posets, orthoalgebras, or Hilbert-space projection lattices requires additional assumptions and is left as a direction for future work.

For clarity, we briefly situate the present structure relative to standard quantum-logical posets. Orthomodular lattices are bounded orthocomplemented lattices satisfying the orthomodular law, with total lattice meet and join operations. Orthomodular posets weaken this setting: joins are required for orthogonal elements, but arbitrary binary joins or meets need not exist. Orthoalgebras provide an even more partial framework based on a partially defined orthogonal sum operation. By contrast, a product Sheffer stroke basic algebra is formulated through a total commutative primitive operation ∣, together with derived recursive sums and additional product/admissibility assumptions used for entropy constructions. Therefore, none of the standard quantum-logical structures is claimed here to be automatically a subclass of the present framework. Any representation of an orthomodular lattice, orthomodular poset, orthoalgebra, or projection lattice by a product Sheffer stroke basic algebra would require additional compatibility conditions relating ∣ to orthocomplementation, orthogonality, admissible sums, order structure, and state additivity.

- **Algebraic elements as abstract propositions.** An element x∈B is treated as an abstract algebraic proposition. In a concrete Hilbert-space projection model, such an element may be associated with a projection operator $P_{x}$ on a Hilbert space H. Under this interpretation, the greatest element 1 corresponds to the identity projection *I*, while the least element 0 corresponds to the zero projection.
- **Sheffer stroke as a commutative primitive connective.** The binary operation x∣y is the commutative primitive connective of the algebra. At the level of an illustrative projection-lattice correspondence, it may be compared with the complement of a meet:
  (252) $$x|y\;⇝\;{\left(P_{x}\wedge P_{y}\right)}^{⊥}.$$
  This expression is symmetric in $P_{x}$ and $P_{y}$ and therefore respects the commutativity required of the Sheffer stroke. In particular, the unary term x∣x formally corresponds to the orthocomplement of *x*, namely
  (253) $$x|x\;⇝\;P_{x}^{⊥}=I-P_{x}.$$
  This correspondence is used only as an interpretive guide and not as a general representation theorem for all product Sheffer stroke basic algebras.
- **Riečan states as probability evaluations.** A state $\tau^{R}$ on B plays the role of a probability assignment on propositions. In Hilbert-space realizations generated by a density operator ρ, this evaluation is represented in Born form by
  (254) $$\tau^{R}\left(x\right)=\mathrm{Tr}\left(\rho P_{x}\right).$$
  Thus, the algebraic state supplies the probabilistic input for the entropic quantities studied in this paper.
- **Partitions as finite algebraic contexts.** A partition $\Xi =(\xi_{1},\ldots ,\xi_{k})$ satisfying
  (255) $$\overset{k}{\underset{i=1}{⨁}}\xi_{i}=1$$
  is treated as a finite algebraic context. In the projection picture, this corresponds to a complete set of pairwise orthogonal projections, equivalently a projection-valued decomposition of the identity, satisfying
  (256) $$\sum_{i=1}^{k}P_{\xi_{i}}=I.$$
  Hence, partitions in the algebraic sense may be compared with observable decompositions or context-dependent outcome sets only when an additional concrete representation is supplied.

Under this analogy, the finite algebra studied in Example 3 should be understood only as a finite algebraic illustration of partition-based entropy calculations in a non-distributive algebraic language. In that interpretation, the partitions Ξ=(γ,δ) and Υ=(ϵ,ζ) represent two distinct algebraic contexts. This does not imply that the example itself constitutes a concrete Hilbert-space measurement model, an orthomodular lattice, or a complete quantum-logical realization. The Riečan state $\tau_{1}^{R}$ then provides the associated probability-type assignment, for example

(257) $$\tau_{1}^{R}\left(\epsilon \right)=\frac{1}{2},\;\tau_{1}^{R}\left(\gamma \right)=\frac{1}{4}.$$

This interpretation becomes particularly relevant when the pseudo-additivity of Tsallis entropy is examined for two partitions. Recall that, in the present algebraic setting, the Tsallis mutual information is defined by

(258) $$I_{\tau_{1}^{R}}^{\alpha }\left(\Xi ;\Upsilon \right)=T_{\tau_{1}^{R}}^{\alpha }\left(\Xi \right)+T_{\tau_{1}^{R}}^{\alpha }\left(\Upsilon \right)-T_{\tau_{1}^{R}}^{\alpha }\left(\left(\Xi |\Upsilon \right)|\left(\Xi |\Upsilon \right)\right).$$

Assume now the algebraic factorization condition used throughout the paper, namely

(259) $$\tau_{1}^{R}\left(\left(\xi_{i}|\upsilon_{j}\right)|\left(\xi_{i}|\upsilon_{j}\right)\right)=\tau_{1}^{R}\left(\xi_{i}\right)\tau_{1}^{R}\left(\upsilon_{j}\right).$$

We stress that this is an internal algebraic independence assumption formulated at the level of the state on the basic algebra. It does not assert the existence of a classical Kolmogorovian event space, nor does it imply that the corresponding contexts arise from jointly measurable physical observables. Rather, it specifies when the state values of the Sheffer stroke joint refinement factorize into the marginal state values of the two partitions.

Under this factorization, the joint Tsallis entropy satisfies

(260) $$\begin{array}{cc} T_{\tau_{1}^{R}}^{\alpha }\left(\left(\Xi |\Upsilon \right)|\left(\Xi |\Upsilon \right)\right)\; & =\frac{1}{\alpha -1}\left(1-\sum_{i=1}^{k}\sum_{j=1}^{l}{\left[\tau_{1}^{R}\left(\xi_{i}\right)\tau_{1}^{R}\left(\upsilon_{j}\right)\right]}^{\alpha }\right)\\ \; & =\frac{1}{\alpha -1}\left(1-\left(\sum_{i=1}^{k}\tau_{1}^{R}{\left(\xi_{i}\right)}^{\alpha }\right)\left(\sum_{j=1}^{l}\tau_{1}^{R}{\left(\upsilon_{j}\right)}^{\alpha }\right)\right). \end{array}$$

Using

(261) $$\sum_{i=1}^{k}\tau_{1}^{R}{\left(\xi_{i}\right)}^{\alpha }=1-\left(\alpha -1\right)T_{\tau_{1}^{R}}^{\alpha }\left(\Xi \right),\;\sum_{j=1}^{l}\tau_{1}^{R}{\left(\upsilon_{j}\right)}^{\alpha }=1-\left(\alpha -1\right)T_{\tau_{1}^{R}}^{\alpha }\left(\Upsilon \right),$$

we obtain

(262) $$\begin{array}{cc} T_{\tau_{1}^{R}}^{\alpha }\left(\left(\Xi |\Upsilon \right)|\left(\Xi |\Upsilon \right)\right)\; & =\frac{1}{\alpha -1}\left(1-\left[1-\left(\alpha -1\right)T_{\tau_{1}^{R}}^{\alpha }\left(\Xi \right)\right]\left[1-\left(\alpha -1\right)T_{\tau_{1}^{R}}^{\alpha }\left(\Upsilon \right)\right]\right)\\ \; & =T_{\tau_{1}^{R}}^{\alpha }\left(\Xi \right)+T_{\tau_{1}^{R}}^{\alpha }\left(\Upsilon \right)-\left(\alpha -1\right)T_{\tau_{1}^{R}}^{\alpha }\left(\Xi \right)\;T_{\tau_{1}^{R}}^{\alpha }\left(\Upsilon \right). \end{array}$$

Substituting this into the definition of mutual information yields

(263) $$I_{\tau_{1}^{R}}^{\alpha }\left(\Xi ;\Upsilon \right)=\left(\alpha -1\right)\;T_{\tau_{1}^{R}}^{\alpha }\left(\Xi \right)\;T_{\tau_{1}^{R}}^{\alpha }\left(\Upsilon \right).$$

This identity explains the effect of the pseudo-additive composition rule under state factorization. In the Shannon limit α→1, the additional Tsallis term disappears, and the mutual information of factorized partitions vanishes. For α>1, the term

(264) $$\left(\alpha -1\right)\;T_{\tau_{1}^{R}}^{\alpha }\left(\Xi \right)\;T_{\tau_{1}^{R}}^{\alpha }\left(\Upsilon \right)$$

may remain nonzero whenever both marginal Tsallis entropies are nonzero. This nonzero value is a consequence of the non-extensive Tsallis composition law, not by itself evidence of operational contextuality or entanglement.

Finally, we emphasize the precise scope of the pseudo-additive residual. The residual $\Delta_{\tau^{R}}^{\alpha }$ introduced in this paper is not a Kochen–Specker contextuality witness and is not an entanglement measure. A genuine operational contextuality witness requires a specified measurement scenario, a compatibility or exclusivity structure, an associated class of noncontextual hidden-variable or ontological models, and a noncontextuality inequality or bound. Depending on the framework, one also needs operational consistency assumptions such as no-disturbance, marginal selectivity, or consistent connectedness. The present residual does not construct such a scenario, does not derive a noncontextual bound, and does not test no-disturbance or marginal-selectivity conditions. It only quantifies, at the Tsallis pseudo-additive entropy level, deviations of the Riečan-state values on the Sheffer stroke joint refinement from the factorized model determined by the corresponding marginal state values. Therefore, it should be understood strictly as a state-dependent algebraic indicator of non-factorizing joint statistics within the present product Sheffer stroke framework.

## 5. Conclusions

This study developed a state-based entropy framework for finite partitions in product Sheffer stroke basic algebras. The main objective was to formulate information-theoretic quantities directly in terms of the Sheffer stroke operation and Riečan-state evaluations, rather than importing a separate Boolean or product-based refinement mechanism. To this end, recursive finite Sheffer sums, admissible partitions, refinement relations, and admissible Sheffer stroke joint refinement constructions were formalized within the algebraic structure. Using Riečan states as probability-type evaluations, Shannon, logical, and Tsallis entropy functionals were introduced for finite partitions.

The principal mathematical contribution is the construction and analysis of the Tsallis entropy functional in this product Sheffer stroke setting. Several basic properties were established, including non-negativity, upper bounds, state concavity, monotonicity under refinement, and subadditivity for α>1. Conditional Tsallis entropy was also formulated, and chain-type identities were obtained under the stated joint refinement marginalization assumptions. The limiting behavior as α→1 was shown, via L’Hôpital’s rule, to recover the corresponding Shannon entropy expressions. In addition, the case α=2 connects the Tsallis formulation with the logical entropy considered earlier in the paper. These results indicate that the proposed framework is consistent with the standard entropy hierarchy while remaining internal to the Sheffer stroke algebraic language.

The paper also examined the pseudo-additive behavior of Tsallis entropy under a state-dependent statistical independence condition. In this setting, independence was understood as pointwise factorization of the Riečan-state values on the Sheffer stroke joint refinement. Under this assumption, the expected Tsallis pseudo-additive identity was recovered. When the factorization condition fails, the associated pseudo-additive residual records the deviation of the actual joint Tsallis entropy from the factorized model determined by the marginal state distributions. Thus, a nonzero residual provides a state-dependent algebraic indicator of deviation from the factorized Tsallis pseudo-additive model.

A possible prospective use of this framework lies in the analysis of generalized logic-circuit designs or finite measurement-like algebraic models whose admissible events are generated from a single primitive connective. For instance, in a Sheffer stroke-based logic circuit, the components of a partition may represent mutually exclusive output classes or admissible algebraic outcome classes generated by repeated NAND-type compositions. Given a Riečan state evaluation on these components, the marginal Tsallis entropies measure the uncertainty associated with individual blocks of the circuit, while the joint Sheffer stroke refinement measures the uncertainty of their combined output behavior. The pseudo-additive residual can then serve as a design-level diagnostic quantity: it vanishes under the stated joint state factorization condition, and a nonzero value signals a deviation from the factorized design model at the Tsallis pseudo-additive entropy level. Similarly, in a finite measurement-like setting, two partitions may represent two compatible families of coarse-grained outcomes, allowing the residual to compare the actually assigned joint refinement weights with those predicted by a factorized state model. This prospective interpretation is intended strictly as a design-level algebraic diagnostic; it does not by itself define a physical implementation, a complete circuit architecture, an entanglement measure, or an operational contextuality witness.

The examples and numerical simulations illustrate how the definitions behave in finite settings and how the pseudo-additive residual vanishes in the factorized case while becoming nonzero for non-factorizing state-induced joint refinements. These computations are intended as consistency checks of the entropy formulas and their scalability at the state-evaluation level, rather than as an exhaustive classification of finite Sheffer stroke basic algebras. The computational complexity analysis shows that marginal entropy calculations scale linearly with the number of partition components, while joint and conditional entropy computations scale with the number of joint refinement components.

Several directions remain open for future work. One natural extension is to study countable partitions in suitable σ-complete Sheffer stroke basic algebras, where additional convergence and measure-theoretic issues must be addressed carefully. Another direction is to formulate relative entropy or divergence-type quantities for pairs of Riečan states within the same algebraic setting. It would also be useful to investigate more concrete representation results connecting product Sheffer stroke basic algebras with known quantum-logical structures, such as orthomodular lattices, orthomodular posets, orthoalgebras, or projection-based models, in order to clarify which additional assumptions would be required for any operational interpretation of the algebraic residual. A further applied direction is to construct explicit finite Sheffer stroke circuit models or finite measurement-like algebraic protocols in which the proposed entropies and residuals can be computed from prescribed or algorithmically assigned state values.

In addition, recent developments on hybrid $\Delta_{h}$ Legendre–Laguerre polynomials and their operational properties [44] suggest another possible direction for extending the present framework. In particular, the use of finite-difference structures, generating functions, monomiality principles, and symmetric identities in such polynomial systems may provide useful tools for constructing parameter-dependent algebraic models in connection with Sheffer stroke partitions. Although the present paper does not develop a polynomial representation of the proposed entropy functionals, these techniques indicate potential avenues for future work, including the study of entropy-like quantities on finite-difference polynomial families, operational representations of partition refinements, and computational models combining generalized polynomial bases with state-based information measures.

## Figures and Tables

**Table 1:** Technical comparison between the MV-algebraic Tsallis entropy frameworks and the present product Sheffer stroke basic algebra approach.

Aspect	Product MV-Algebras [36]	MV-Algebras [37]	Present Sheffer Stroke Framework
Primitive structure	MV-algebra enriched with an additional product operation.	MV-algebra with the standard MV-operations; refinements are handled through the Riesz refinement mechanism.	Product Sheffer stroke basic algebra formulated with the Sheffer stroke as the primitive algebraic operation.
Partition mechanism	A finite tuple, not necessarily with distinct entries, whose MV-sum is the unit.	A finite sequence, not necessarily with distinct entries, whose sum is equal to 1.	A finite mutually orthogonal nonzero family of pairwise distinct elements whose recursive Sheffer sum is 1.
Joint construction	The joint partition is defined by product components $x_{i}\cdot y_{j}$.	A common refinement is obtained through the Riesz refinement mechanism.	Bipartite joint components are generated natively by $\zeta_{ij}=\left(\xi_{i}|\upsilon_{j}\right)|\left(\xi_{i}|\upsilon_{j}\right)$ and, after null entries are omitted, are proved to satisfy orthogonality and completeness, hence forming bipartite joint partitions; whereas sequential triple refinements require explicit marginalization conditions.
Main entropy results	Tsallis entropy, conditional Tsallis entropy, subadditivity for α>1, pseudo-additivity under an independence/factorization condition, and dynamical-system entropy.	Tsallis entropy of partitions and MV-dynamical systems; submeasure property and invariance under isomorphisms.	Sheffer stroke analogues of the corresponding entropy properties are established under the stated orthogonal distributivity, Riečan-state additivity, and, for conditional chain rules, triple marginalization conditions.
What is gained	The product operation gives a direct product-type joint construction.	The Riesz refinement machinery provides common refinements of partitions.	Stroke-based minimalism: partition and joint refinement components are generated through ∣, while entropy values are evaluated through the associated Riečan state.
What is lost or restricted	The framework requires an additional product operation in the signature.	The framework relies on the Riesz refinement machinery for common refinements.	Recursive Sheffer sums require a fixed evaluation convention; neither an associative product operation nor an unconditional Riesz refinement-based triple joint construction is assumed.
New contribution here	–	–	Stroke-native partitions and joint refinements; entropy constructions based on state values of Sheffer-generated components; pseudo-additive residual recording deviations from factorized joint state statistics at the Riečan-state level.

**Table 2:** ∣-operation on *M*.

∣	0	β	γ	δ	ε	1
0	1	1	1	1	1	1
β	1	δ	1	1	δ	δ
γ	1	1	ε	ε	1	ε
δ	1	1	ε	β	1	β
ε	1	δ	1	1	γ	γ
1	1	δ	ε	β	γ	0

**Table 3:** Cayley table of the ∣-operation on M.

∣	0	γ	δ	ϵ	ζ	η	θ	1
0	1	1	1	1	1	1	1	1
γ	1	δ	1	1	δ	δ	1	δ
δ	1	1	γ	ζ	η	ζ	η	γ
ϵ	1	1	ζ	ζ	1	ζ	1	ζ
ζ	1	δ	η	1	ϵ	δ	η	ϵ
η	1	δ	ζ	ζ	δ	θ	1	θ
θ	1	1	η	1	η	1	η	η
1	1	δ	γ	ζ	ϵ	θ	η	0

**Table 4:** Comparison of Shannon, logical, and Tsallis entropy functionals on finite partitions of product Sheffer stroke basic algebras equipped with Riečan states.

Entropy Type	Definition on Partition Ξ	Key Properties in This Paper	Advantages	Limitations
**Shannon**	$H_{\tau^{R}}\left(\Xi \right)=-\sum_{i=1}^{k}\tau^{R}\left(\xi_{i}\right)\log\tau^{R}\left(\xi_{i}\right)$	Baseline extensive entropy; admits conditional form $H_{\tau^{R}}\left(\Xi /\Upsilon \right)$; satisfies subadditivity for joint Sheffer stroke refinements; recovered as the limit of Tsallis entropy as α→1.	Provides the classical information-theoretic benchmark; has a clear probabilistic interpretation; useful for comparison with standard Kolmogorov–Shannon uncertainty.	Strictly extensive; less sensitive to non-extensive or correlation-driven effects; does not by itself encode the pseudo-additive residual generated by the Tsallis framework.
**Logical**	$H_{\tau^{R}}^{l}\left(\Xi \right)=\sum_{i=1}^{k}\left(\tau^{R}\left(\xi_{i}\right)-\tau^{R}{\left(\xi_{i}\right)}^{2}\right)=1-\sum_{i=1}^{k}\tau^{R}{\left(\xi_{i}\right)}^{2}$	Obtained as the special case $T_{\tau^{R}}^{2}\left(\Xi \right)$ of Tsallis entropy; satisfies logical subadditivity; admits a conditional logical form based on squared marginal and joint state values.	Computationally simple; avoids logarithms; directly measures distinction or diversity among partition components; especially convenient for finite algebraic examples.	Corresponds to a fixed quadratic case; lacks the tunable sensitivity of Tsallis entropy; less flexible for modeling varying degrees of non-extensivity.
**Tsallis**	$T_{\tau^{R}}^{\alpha }\left(\Xi \right)=\frac{1}{\alpha -1}\left(1-\sum_{i=1}^{k}\tau^{R}{\left(\xi_{i}\right)}^{\alpha }\right)$ α∈(0,1)∪(1,∞)	Parametric non-extensive entropy; satisfies bounds, state concavity, monotonicity under refinement, conditional chain-type identities under joint refinement marginalization assumptions, subadditivity for α>1, and pseudo-additivity under statistical independence.	Provides a tunable family of entropy measures; captures non-extensive behavior; includes logical entropy at α=2 and Shannon entropy in the limiting case α→1; enables the pseudo-additive residual as an indicator of non-factorizing joint statistics.	Depends on the choice of the index α; some results require α>1; the pseudo-additive residual is state-dependent and should not be interpreted as a complete operational contextuality or entanglement measure.

## Data Availability

The original contributions presented in this study are included in the article. Further inquiries can be directed to the corresponding author.

## Appendix A. Computational Complexity and Scalability of Algorithms

In this appendix, we analyze the computational complexity and scalability of Algorithms 1–4. This appendix is included to clarify the algorithmic cost of the entropy computations without interrupting the main mathematical development of the paper.

Let $\Xi =(\xi_{1},\ldots ,\xi_{k})$ and $\Upsilon =(\upsilon_{1},\ldots ,\upsilon_{l})$ be finite partitions in a product Sheffer stroke basic algebra B=(B;∣). We assume a standard unit-cost computational model in which a single evaluation of the Sheffer stroke operation ∣ and a single evaluation of the Riečan state $\tau^{R}$ require constant time. Under this assumption, the dominant cost is determined by the number of marginal or joint partition components that must be evaluated. If either the Sheffer stroke operation or the state evaluation is implemented by a non-constant-time subroutine, the complexities reported below should be multiplied by the corresponding evaluation cost.

**Table A1:** Computational complexity of Algorithms 1–4 under the unit-cost model for ∣ and $\tau^{R}$.

Algorithm	Computed Quantities	Time Complexity	Auxiliary Space
Algorithm 1	$H_{\tau^{R}}\left(\Xi \right)$ and $H_{\tau^{R}}\left(\Xi /\Upsilon \right)$	O(kl)	O(1)
Algorithm 2	$H_{\tau^{R}}^{l}\left(\Xi \right)$ and $H_{\tau^{R}}^{l}\left(\Xi /\Upsilon \right)$	O(kl)	O(1)
Algorithm 3	$T_{\tau^{R}}^{\alpha }\left(\Xi \right)$	O(k)	O(1)
Algorithm 4	$T_{\tau^{R}}^{\alpha }\left(\Xi \right)$ and $T_{\tau^{R}}^{\alpha }\left(\Xi /\Upsilon \right)$	O(kl)	O(1)

Algorithm 1 computes the marginal Shannon entropy of Ξ through a single loop over *k* elements and therefore requires O(k) operations for the marginal part. Its conditional entropy component evaluates the joint terms

(A1) $$\left(\xi_{i}|\upsilon_{j}\right)|\left(\xi_{i}|\upsilon_{j}\right),\;i=1,\ldots ,k,\;j=1,\ldots ,l,$$

which requires a nested loop over kl pairs. Hence, the total running time of Algorithm 1 is O(kl). Since only scalar accumulators are needed, and the full joint partition does not need to be stored explicitly, the auxiliary space complexity is O(1).

Algorithm 2 has the same asymptotic complexity. The marginal logical entropy is computed in O(k) time, while the conditional logical entropy requires evaluating the squared state values of all kl joint components. Therefore, its total time complexity is O(kl), with O(1) auxiliary space when joint components are computed on demand.

Algorithm 3 computes only the marginal Tsallis entropy of a single partition Ξ. It performs one pass over the *k* elements of Ξ and accumulates the powers $\tau^{R}{\left(\xi_{i}\right)}^{\alpha }$. Thus, its time complexity is O(k) and its auxiliary space complexity is O(1).

Algorithm 4 computes both the marginal Tsallis entropy and the conditional Tsallis entropy. The marginal part requires O(k) operations, while the conditional part evaluates all joint components associated with Ξ and Υ. Consequently, the dominant term is O(kl), and the total time complexity is O(kl). As in the previous algorithms, the auxiliary space complexity remains O(1) if the joint values are computed and accumulated without storing the entire joint partition. If the complete joint refinement is stored explicitly rather than evaluated on demand, the space complexity increases to O(kl).

From the scalability viewpoint, the marginal entropy computations scale linearly with the number of elements in the input partition. In contrast, conditional and joint entropy computations scale linearly with the number of joint refinement components. For two partitions of sizes *k* and *l*, this number is kl. If the partitions have comparable size, say k≈l≈n, then the joint entropy computations scale quadratically as $O(n^{2})$.

More generally, if *s* partitions of approximately equal size *n* are jointly refined, the number of possible joint components may grow as $O(n^{s})$. Thus, the main scalability limitation is the combinatorial growth of joint refinements, rather than the evaluation of the entropy functions themselves. This behavior is analogous to the standard computational cost encountered in multi-variable entropy calculations.

In practical implementations, the effective running time can be reduced when the joint partition contains null components. If these null components are omitted, the complexity becomes proportional to the number of non-null joint entries, denoted by $N_{\mathrm{nz}}$, rather than the full product kl. In such sparse cases, the conditional entropy algorithms scale as $O(N_{\mathrm{nz}})$. Moreover, because each joint component can be evaluated independently, the nested-loop computations appearing in Algorithms 1, 3, and 4 are naturally parallelizable. Therefore, the proposed algorithms are scalable for moderate finite partitions and remain computationally feasible for larger sparse or parallelized settings.

## Appendix B. Larger-Scale Numerical Simulations

In addition to the small finite examples discussed in the main text, we performed larger-scale numerical simulations to examine the behavior of the proposed entropy and residual formulas on prescribed state-induced joint arrays. These simulations are not obtained by constructing or enumerating complete finite product Sheffer stroke basic algebra operation tables. Instead, they are carried out at the level of Riečan-state values assigned to finite joint arrays satisfying the required marginal constraints. Accordingly, the simulations should be interpreted as numerical tests of the state-induced entropy layer of the theory, rather than as a construction, classification, or verification of large finite Sheffer stroke basic algebras.

Let

$$\Xi =\left(\xi_{1},\ldots ,\xi_{k}\right),\;\Upsilon =\left(\upsilon_{1},\ldots ,\upsilon_{l}\right)$$

be two finite partitions. We denote the marginal Riečan-state weights by

$$p_{i}=\tau^{R}\left(\xi_{i}\right),\;q_{j}=\tau^{R}\left(\upsilon_{j}\right),$$

where

$$\sum_{i=1}^{k}p_{i}=1,\;\sum_{j=1}^{l}q_{j}=1.$$

The state values assigned to the Sheffer stroke joint refinement are represented by a joint array

$$r_{ij}=\tau^{R}\left(\left(\xi_{i}|\upsilon_{j}\right)|\left(\xi_{i}|\upsilon_{j}\right)\right),\;\sum_{i=1}^{k}\sum_{j=1}^{l}r_{ij}=1.$$

The statistically independent case is represented by the factorized refinement

$$r_{ij}^{\left(0\right)}=p_{i}q_{j}.$$

To generate non-factorizing refinements while preserving the same marginal state weights, we construct a second joint array

$$c_{ij}\geq 0$$

satisfying

$$\sum_{j=1}^{l}c_{ij}=p_{i},\;\sum_{i=1}^{k}c_{ij}=q_{j}.$$

Then, for a parameter 0≤λ≤1, we define

$$r_{ij}^{\left(\lambda \right)}=\left(1-\lambda \right)p_{i}q_{j}+\lambda c_{ij}.$$

Since both $p_{i}q_{j}$ and $c_{ij}$ have the same marginals *p* and *q*, the perturbed refinement also satisfies

$$\sum_{j=1}^{l}r_{ij}^{\left(\lambda \right)}=p_{i},\;\sum_{i=1}^{k}r_{ij}^{\left(\lambda \right)}=q_{j}.$$

Therefore, the parameter λ controls the deviation from statistical independence without changing the marginal state distributions. Sparse or constrained non-factorizing couplings are obtained by imposing a support mask on $c_{ij}$ and then applying iterative proportional fitting to match the prescribed marginals. For 0<λ<1, the resulting refinement combines the full factorized component with the constrained coupling; for λ=1, the joint refinement coincides with the constrained coupling itself. This construction models finite state-induced Sheffer stroke joint refinements in which some joint refinement components may be structurally suppressed while the prescribed marginal Riečan-state values remain fixed.

For each simulated refinement, we compute the Tsallis entropy of the marginals and of the joint refinement. For α>0, α≠1, we use

$$T_{\alpha }\left(p\right)=\frac{1}{\alpha -1}\left(1-\sum_{i=1}^{k}p_{i}^{\alpha }\right),$$

$$T_{\alpha }\left(q\right)=\frac{1}{\alpha -1}\left(1-\sum_{j=1}^{l}q_{j}^{\alpha }\right),$$

and

$$T_{\alpha }\left(R^{\left(\lambda \right)}\right)=\frac{1}{\alpha -1}\left(1-\sum_{i=1}^{k}\sum_{j=1}^{l}{\left(r_{ij}^{\left(\lambda \right)}\right)}^{\alpha }\right).$$

The pseudo-additive residual is then computed as

$$\Delta_{\alpha }^{\left(\lambda \right)}=T_{\alpha }\left(R^{\left(\lambda \right)}\right)-\left[T_{\alpha }\left(p\right)+T_{\alpha }\left(q\right)+\left(1-\alpha \right)T_{\alpha }\left(p\right)T_{\alpha }\left(q\right)\right].$$

In the factorized case λ=0, the residual vanishes up to floating-point precision:

$$\Delta_{\alpha }^{\left(0\right)}\approx 0.$$

For λ>0, the residual is generally nonzero, because the joint refinement no longer factorizes pointwise as $r_{ij}=p_{i}q_{j}$, even though the marginal state weights remain unchanged. Hence, in this simulation protocol, the pseudo-additive residual isolates the deviation from statistical independence rather than a change in the marginal distributions.

The following Python code was used to generate the numerical results. A fixed random seed was used for reproducibility. The reported computational time refers to the entropy and residual evaluation step, not to the random generation of the input arrays. In the numerical simulations, a standard regularization constant ($10^{-12}$) is applied to masked elements to ensure the numerical stability and strict convergence of the Iterative Proportional Fitting (IPF) algorithm.

**Listing A1.** Python code for larger-scale numerical simulations of state-induced Sheffer stroke joint refinements.

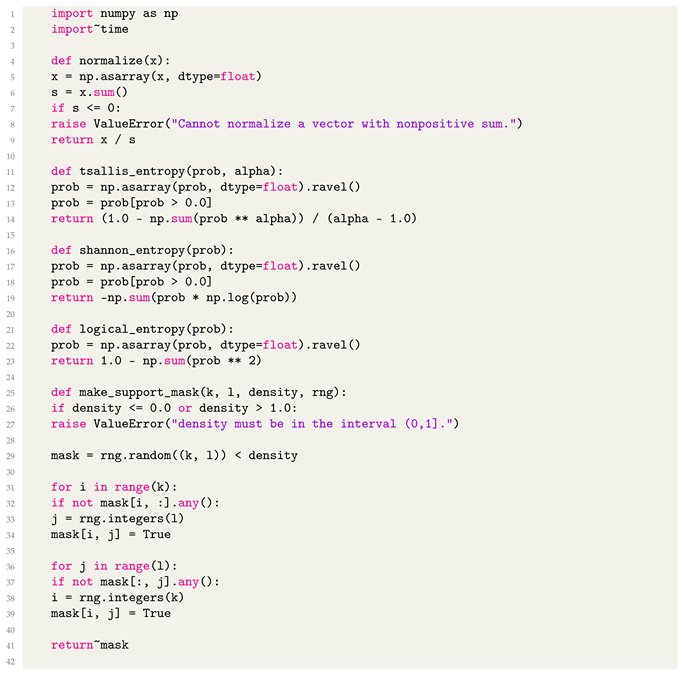

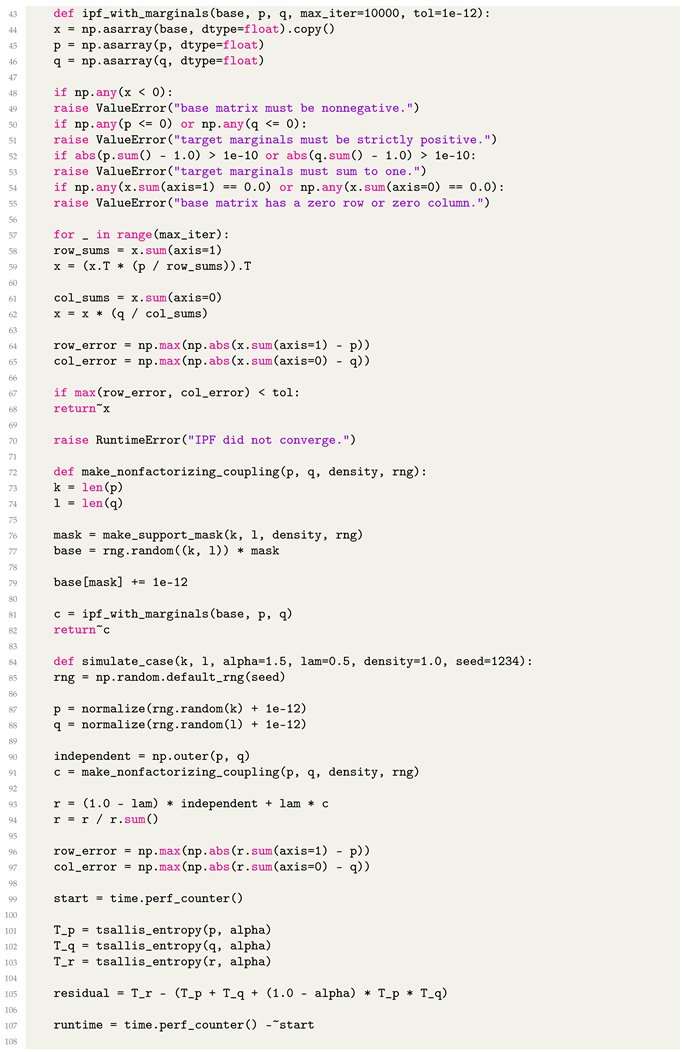

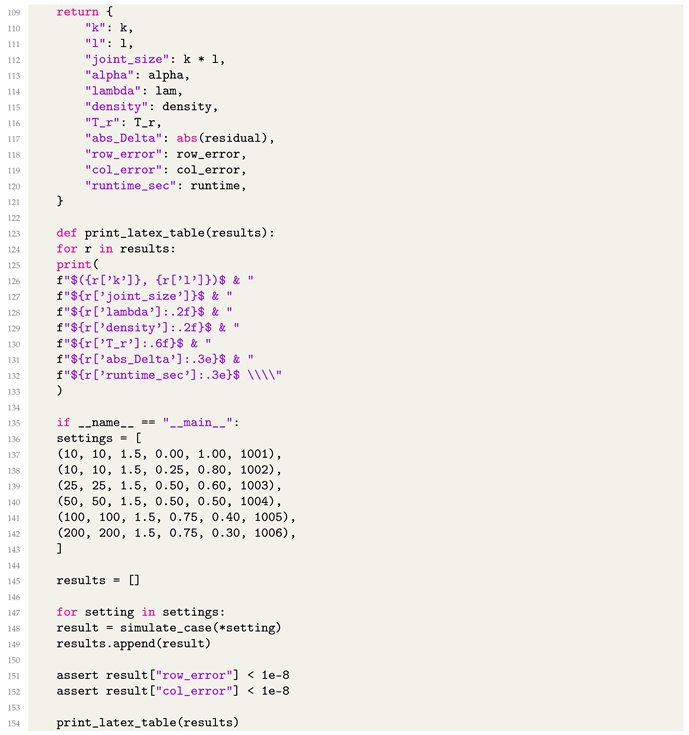

**Table A2:** Larger-scale numerical simulations for finite state-induced Sheffer stroke joint refinements with α=1.5. The parameter λ controls deviation from factorization while preserving the prescribed marginals. The residual vanishes up to floating-point precision in the factorized case and becomes nonzero for non-factorizing refinements.

(k,l)	kl	λ	Density	$T_{\alpha }\left(R\right)$	$|\Delta_{\alpha }|$	Time (s)
(10,10)	100	0.00	1.00	1.691287	$2.220\times 10^{-16}$	$2.700\times 10^{-5}$
(10,10)	100	0.25	0.80	1.716958	$3.826\times 10^{-3}$	$1.979\times 10^{-5}$
(25,25)	625	0.50	0.60	1.888702	$1.197\times 10^{-2}$	$2.788\times 10^{-5}$
(50,50)	2500	0.50	0.50	1.941825	$7.564\times 10^{-3}$	$5.729\times 10^{-5}$
(100,100)	10,000	0.75	0.40	1.962717	$1.169\times 10^{-2}$	$1.661\times 10^{-4}$
(200,200)	40,000	0.75	0.30	1.978909	$8.083\times 10^{-3}$	$5.822\times 10^{-4}$

The simulations show the expected behavior. In the factorized case λ=0, the pseudo-additive residual is zero up to floating-point precision, as indicated by the value $2.220\times 10^{-16}$. For λ>0, the residual becomes nonzero while the prescribed marginal state distributions remain fixed. This confirms that, in the simulated cases, a nonzero residual reflects deviation from the factorized pseudo-additive model while the marginal distributions are kept fixed. Moreover, as the number of joint entries grows from 100 to 40,000, the entropy evaluation time remains consistent with the expected linear dependence on the number of joint refinement components, in agreement with the complexity analysis in Appendix A. These numerical results demonstrate that the entropy and residual formulas can be evaluated efficiently on larger finite state-induced joint arrays satisfying prescribed marginal constraints.
